# Trends in socioeconomic inequalities in obesity among Korean adolescents: the Korea Youth Risk Behavior Web-based Survey (KYRBS) 2006 to 2020

**DOI:** 10.4178/epih.e2023033

**Published:** 2023-03-07

**Authors:** Eunji Kim, Ga Bin Lee, Dong Keon Yon, Hyeon Chang Kim

**Affiliations:** 1 Department of Preventive Medicine, Yonsei University College of Medicine, Seoul, Korea; 2 Center for Digital Health, Medical Science Research Institute, Kyung Hee University College of Medicine, Seoul, Korea; 3 Institute for Innovation in Digital Healthcare, Yonsei University College of Medicine, Seoul, Korea

**Keywords:** Adolescent, Body mass index, Pediatric obesity, Socioeconomic disparities in health

## Abstract

**OBJECTIVES:**

This study investigated recent trends in the prevalence of obesity among Korean adolescents and explored socioeconomic disparities in obesity.

**METHODS:**

This study used annual self-reported data on height, weight, and socioeconomic information from the Korea Youth Risk Behavior Web-based Survey from 2006 to 2020. With a 95.8% response rate, the sample consisted of 818,210 adolescents. Obesity prevalence was calculated according to 4 socioeconomic indicators (household income, father’s educational attainment, mother’s educational attainment, and urbanicity). Socioeconomic inequality was quantified using the relative index of inequality (RII).

**RESULTS:**

The overall prevalence of obesity increased, doubling from 5.9% in 2006 to 11.7% in 2020. Boys and high school students showed a higher prevalence. The RIIs in household income and parental educational attainments significantly increased with time, indicating a growing inequality in obesity. Socioeconomic disadvantages had a greater influence on obesity among girls. The most recent RII values for boys were 1.25 for income, 1.79 for the father’s education, and 1.45 for the mother’s education, whereas the corresponding values for girls were 2.49, 3.17, and 2.62, respectively.

**CONCLUSIONS:**

These findings highlight growing inequalities in adolescent obesity according to household income and parental educational attainments, especially for girls and middle schoolers.

## GRAPHICAL ABSTRACT

[Fig f3-epih-45-e2023033]

## INTRODUCTION

Obesity in childhood and adolescence is associated with various comorbidities such as hypertension, dyslipidemia, asthma, type 2 diabetes, polycystic ovary syndrome, and other physiological symptoms and disorders [1]. Furthermore, it increases the risk of obesity and related morbidities in adulthood, including cardiovascular disease [2]. In this context, the escalating prevalence of childhood obesity has raised global concerns. Since the early 2000s, many studies have reported a plateau of childhood obesity in high-income countries, even though the prevalence remains high [3].

However, this stabilization of the prevalence does not seem to be experienced by all socioeconomic groups and regions. Previous studies have demonstrated that socioeconomic status and urbanicity are associated with childhood obesity [4,5]. A few studies have highlighted secular changes in socioeconomic gradients; in many developed countries, such as the United Kingdom, the United States, and Australia, a decline in obesity among socioeconomically advantaged children has masked an increase in marginalized children, leading to stagnation of the overall prevalence [6-8].

In Korea, despite some achievements in risk modification for non-communicable diseases, the national prevalence of obesity has continued to rise; the proportion of adults with a body mass index (BMI) of 25 kg/m^2^ or higher increased from 29.7% in 2009 to 38.4% in 2020, and that of adults with a BMI of 30 kg/m^2^ or higher doubled to 8.2% [9,10]. Meanwhile, childhood obesity has not been extensively investigated since obesity at a young age was often minimized. The burden of childhood obesity has increased over time in Korea. The obesity rate among children aged 6-18 years increased from 1.7% to 11.1% for boys and from 2.6% to 8.9% for girls from 1979 to 2005 [11]. Based on the slowing rate of increase around 2005, a decrease in the obesity rate or stabilization was anticipated [12]. However, a recent study showed that the prevalence of pediatric obesity in Korea continued to increase, reaching 15% in 2017 [13].

Additionally, although considerable research has identified associations between socioeconomic factors and obesity, studies on trends in socioeconomic disparities in adolescent obesity are scarce in Korea. This study aimed to investigate recent trends in the prevalence of obesity and socioeconomic disparities in obesity among Korean adolescents from 2006 to 2020.

## MATERIALS AND METHODS

### Study population

This study used annual data from the Korea Youth Risk Behavior Web-based Survey (KYRBS) from 2006 to 2020. The KYRBS is a nationwide cross-sectional survey that assesses adolescents’ health status and health-risk behaviors [14]. The Korea Disease Control and Prevention Agency (KDCA) and the Ministry of Education have been conducting the survey annually since 2005 [14]. The survey employs a stratified multistage cluster sampling design to obtain a nationally representative sample of middle school and high school students in Korea [15]. All students of each selected classroom participated anonymously by completing a self-administered web-based questionnaire in each sampled school [15]. Detailed information on the survey has been published in separate articles [15].

We analyzed all survey data collected so far, except for those in the first year of the KYRBS (2005), as it did not provide age information in months. During the study period from 2006 to 2020, an annual average of 68,000 students participated from 800 schools (400 middle and 400 high schools) by sampling 2% of total middle school and high school students and 15% of total schools in Korea. The response rate was approximately 95.8% (90.9-97.7%) [14]. We excluded participants aged less than 144 months or more than 227 months and those with missing data for sex, age, weight, height, or any socioeconomic indicator. After excluding 5,618 individuals (0.7% of the total survey respondents), the analytic sample for 15 years consisted of 818,210 adolescents aged between 12 years and 18 years.

### Measurements

Students’ height and weight were self-reported in the survey, from which BMI was calculated as weight divided by height squared (kg/m^2^). Body size and growth in childhood substantially change with age; therefore, the definition of adolescent obesity requires taking into consideration age and sex [16,17]. In this study, adolescent obesity was defined as a sex-specific BMI-for-age (in months) of the 95th percentile or greater based on the 2017 Korean National Growth Charts (KNGC). The KNGC has been nationally used as a Korean standard to evaluate children’s growth and health [16,18]. It was developed using advanced statistical methods used in the Centers for Disease Control and Prevention (CDC) Growth Charts and the World Health Organization Growth Reference (WHO-GR) [16].

We used 4 indicators of socioeconomic position (SEP) to investigate socioeconomic inequalities in adolescent obesity: household income, father’s educational attainment, mother’s educational attainment, and urbanicity. Household income was subjectively measured on a five-point scale (highest, mid-high, middle, mid-low, and lowest) in the KYRBS. We merged and categorized them into three groups: high (highest and mid-high), middle, or low (mid-low and lowest). Parental education attainments were self-reported as basic education or less (middle school—compulsory education of 9 years and below), upper secondary education (high school), and tertiary education or above (college, university, or above). Urbanicity was determined as the location of the sampled schools. From the survey design process, the KDCA identified middle and high schools in each administrative district across the country and classified them into three groups (metropolitan cities, other cities, and rural areas) based on the size of the population and urbanization of the district [15].

### Statistical analysis

The sampling weights provided by the KDCA were applied in our analyses to extrapolate the results to the general youth population in Korea. The general characteristics of the study population were presented as weighted means with standard deviations or weighted percentages with 95% confidence intervals (CIs). We calculated the overall prevalence of obesity in the total study population and subgroups by sex or by stage of school, and then the prevalence in each subgroup according to socioeconomic status. Trend analyses were conducted for general characteristics, anthropometric measures, and overall prevalence using a linear regression model with the survey year variable.

Socioeconomic inequalities in adolescent obesity were quantified using the prevalence ratio (PR), relative index of inequality (RII), prevalence difference (PD), and slope index of inequality (SII). The PR of each socioeconomic subgroup compared to the reference was calculated from SAS PROC GENMOD’s log-binomial regression analyses (version 9.4; SAS Institute Inc., Cary, NC, USA) [19]. To summarize and compare relative health inequalities between populations with different proportions of socioeconomic subgroups, the RII was also employed [20]. The RII represents the ratio of the health status of those at the highest SEP to that of those at the lowest SEP of the socioeconomic distribution [20]. Categorical socioeconomic variables were ordered and rescaled to reflect a continuous range. Each SEP subgroup was assigned as the midpoint of its relative frequency range and cumulatively ranked from 0 (hypothetically most advantaged) to 1 (hypothetically most disadvantaged). The relative rank variable and obesity status were included as independent and dependent variables, respectively, in log-binomial regression models, and the RII based on the PR was obtained. An RII of 1.0 denotes no inequality [21]. Values more than 1.0 indicate that worse outcomes are concentrated in the disadvantaged, whereas those less than 1.0 indicate a concentration of adverse outcomes in the advantaged [21]. The further the value from 1.0, the greater the magnitude of inequality [21]. RIIs for each of the 4 socioeconomic indicators were calculated for each year. Time trends in socioeconomic inequalities were determined by estimating the p-value for an interaction term between the relative rank variable and the survey year variable in the models.

We additionally examined the RII based on the odds ratio (OR) and the SII based on the PD, using results obtained from surveylogistic regression models and linear regression using SAS PROC GENMOD with the link= identity option, respectively [19]. The SII based on the PD represents absolute inequalities by summarizing differences in obesity prevalence according to a hierarchical socioeconomic ranking [20,22].

### Ethics statement

The KYRBS was officially approved by the Institutional Review Board of the KDCA. All study participants anonymously completed the survey, and the KDCA provided de-identified raw data after registration.

## RESULTS

Table 1 shows the distribution of the total study population according to sex, stage of school, and each of the 4 socioeconomic indicators. The proportion of students in affluent households or with highly educated parents increased over time, while the proportion of those with the lowest economic status or with the least educated parents decreased. The number of participants in metropolitan and rural areas decreased during the study period (Supplementary Material 1).

**Figure 1.**
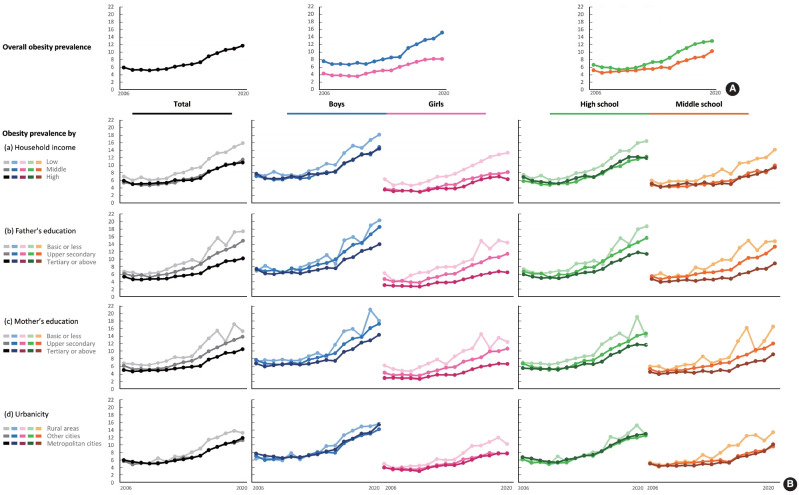
Trends in obesity prevalence among the Korean adolescents from 2006 to 2020. (A) Overall prevalence of the total study population, boys and girls, and middle- and high school students. (B) Obesity prevalence of the total and subgroups according to each of four socioeconomic factors.

Table 2 presents the self-reported anthropometric measures and the overall prevalence of obesity defined using various growth references. Self-reported height, weight, and calculated BMI steadily increased. Based on the 2017 KNGC, which was used as the main reference in our study, the overall prevalence of obesity remained stable (approximately 5%) in the late 2000s, but increased to 11.7% by 2020 in the total study population. The values in all subgroups also increased (Figure 1A and Table 2). This increasing trend in prevalence was consistent whether we defined obesity as a BMI ≥ 95th percentile (using the 2017 KNGC or the WHO-GR criteria) or a BMI> median+2 standard deviations based on the WHO-GR [17]. According to all definitions, boys had a greater prevalence of obesity than girls. The prevalence of obesity in high school students was slightly higher than that of middle school students based on the 2017 KNGC; however, the opposite order was observed when WHO-GR was applied (Supplementary Material 2).

Figure 1B shows the prevalence of obesity according to socioeconomic status in the total sample and the subgroups (boys and girls, high and middle school students). Overall, a similar trend was observed for all 4 socioeconomic indicators. At the beginning of the study period, there was no significant difference in prevalence among the 3 SEP groups. However, this discrepancy became more prominent over time. The group with the lowest household income had a higher prevalence of obesity than the middle-income and highest-income groups. Pronounced gradual differences were observed between the 3 SEP groups, divided by fathers’ and mothers’ education levels. The absolute figures of prevalence were higher in boys and high school students than in girls and middle school students. The recent disparity between the lowest SEP group and other groups was more significant among girls and middle school students (Supplementary Material 3).

**Figure 2.**
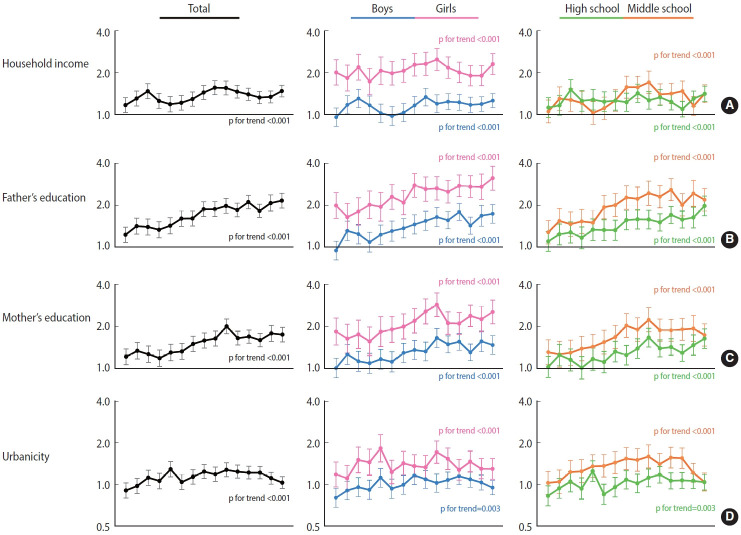
The trends of relative index of inequality based on prevalence ratio according to (A) household income, (B) father’s educational attainment, (C) mother’s educational attainment, and (D) urbanicity of school from 2006 to 2020. The closed vertical lines represent the 95% confidence intervals.

Figure 2 shows time trends in socioeconomic inequalities using PR-based RIIs. Overall, socioeconomic inequalities in adolescent obesity were present and increased during the study period. Although all socioeconomic inequalities were on the rise, a greater disparity in obesity was observed according to the fathers’ and mothers’ educational attainments. The most recent RIIs were 1.50 (95% CI, 1.48 to 1.52) for household economic status, 2.18 (95% CI, 2.14 to 2.22) for the father’s education level, and 1.75 (95% CI, 1.72 to 1.78) for the mother’s education level (Supplementary Materials 4 and 5). Girls showed higher values: 2.49 (95% CI, 2.42 to 2.55) for income, 3.17 (95% CI, 3.08 to 3.26) for paternal education, and 2.62 (95% CI, 2.55 to 2.70) for maternal education compared to boys (1.25; 95% CI, 1.23 to 1.27, 1.79; 95% CI, 1.75 to 1.83, 1.45; 95% CI, 1.41 to 1.48, respectively). Similarly, the recent RIIs of middle school students were higher than those of high school students for most socioeconomic indicators. The disparity in obesity was relatively less affected by urbanicity than other factors; the RIIs for the total study population were near 1, although its trend was on the rise. When divided into subgroups, rural disadvantages were more pronounced in girls and middle school students. Boys and high school students showed an urban disadvantage at the beginning of the study.

Across all socioeconomic factors and subgroups, these increasing trends of inequality were consistently observed when we calculated RIIs based on ORs (Supplementary Materials 6 and 7). We also identified an increasing trend in the SII for all socioeconomic indicators studied. In particular, absolute inequalities in fathers’ and mothers’ educational attainments showed the greatest values (Supplementary Material 8).

## DISCUSSION

Using 15-year trend analyses of a nationwide representative survey of Korean adolescents, this study showed that both the prevalence of adolescent obesity and socioeconomic inequalities in adolescent obesity increased between 2006 and 2020. A greater disparity was observed between students with highly educated parents and those with less educated parents, which significantly widened. In subgroup analyses, all 4 types of socioeconomic inequalities were found in all sex and school-stage subgroups. However, their magnitudes were greater in girls and middle school students. Notably, the absolute prevalence rates were higher in boys and high school students.

Korea has a relatively well-established health system and intends to respond to the rapid epidemiological transition of obesity [23]. Despite academic and political efforts, obesity is increasing not only in adults but also in children, as observed in the current study [9]. Given that adolescent obesity seems to have stabilized in many developed countries, the increasing prevalence in Korea is notable [3]. This would threaten the current health of Korean youths and aggravate the burden on health care in the future. Meanwhile, the high obesity rates among children appear to be valid, even after considering the likelihood of short stature. Although high BMI due to low height was not expected to be as prevalent in Korea as in low-income or middle-income countries, it could be misinterpreted as excess body weight. We additionally analyzed the prevalence of stunting, which was defined as height-for-age less than 2 standard deviations from the median based on the 2017 KNGC. A negative correlation was observed between SEP and stunting, although the prevalence was low (Supplementary Material 9). When calculating cases of a high BMI due to stunting, the absolute number was extremely low (Supplementary Material 10). Hence, the interpretation seems valid that short stature was unlikely to lead to a high obesity rate among Korean youth, and the prevalence of excess body weight increased.

Moreover, the growing inequality of adolescent obesity is alarming; the increase in obesity risk among socioeconomically deprived children was greater than the rise among the advantaged. One study in Australia reported RIIs of 1.16 for boys and 1.15 for girls in annual BMI growth according to adolescents’ SEP defined with a composite variable [24]. Another study using the combined data from 11 European cohorts demonstrated an RII of 1.58 (95% CI, 1.34 to 1.85) for overweight and 2.61 (95% CI, 2.10 to 3.23) for obesity according to maternal education [25]. Recognizing limitations in the global comparison of socioeconomic inequality due to sample characteristics and study designs, Korea’s high inequality presented as RIIs highlights the need to closely monitor the social gradient in adolescent obesity.

Previous studies have found that household economic status has a substantial impact on obesity in the youth. Nevertheless, the direction of inequality differs according to the economic level of the country or racial and cultural backgrounds [26,27]. Obesity is concentrated among the advantaged in developing countries, but among the disadvantaged in high-income countries [26]. A study in the United States demonstrated a protective effect of high family income against obesity in White, but not in Black children [27]. This suggests that various factors are associated with household economic level and the discriminated risk of adolescent obesity: social perceptions of obesity, affordability of a healthy diet, accessibility to healthcare, health literacy, physical activity, and so forth [28].

In our study, the influence of inequality according to parents’ educational attainments was greater than that of the other indicators, and the increasing trend was more evident. A negative correlation between parental educational attainments and childhood obesity has also been observed in other countries. One study pointed out that the stabilized prevalence of obesity masked the increasing disparity according to parents’ education levels [6]. Since 2002, youth who had highly educated parents showed a decreasing trend in the prevalence of obesity, whereas obesity in those having parents with low educational attainment continued to increase. The escalating trend in inequality among Korean youth differs from that of other countries: the prevalence increased in all socioeconomic groups in Korea, but with a more substantial magnitude in the socioeconomically disadvantaged.

Compared to other socioeconomic factors, the effect of urbanicity on adolescent obesity was fairly small. Among boys and high schoolers, the recent RIIs were around the value of 1, indicating no significant inequality. Only a slight rural disadvantage was observed among female students and middle school students. Previous research on urban-rural disparities has shown various results by country: Obesity was concentrated in urban areas in low-income and middle-income countries, but in rural areas in high-income countries [26]. Moreover, other studies reported no differences in obesity prevalence between rural and urban areas in 10 European countries [29]. Another study provided possible explanations for our findings: Rural disadvantages may be due to high prevalence of energy-dense dietary intake, sedentary lifestyle, and physical inactivity among girls and middle school students in rural areas [5,30]. The lack of urban-rural differences, however, in boys and high school students could imply that the possible mechanisms linking urbanicity and adolescent obesity are sexspecific in Korea. Further research is required to comprehend the sex-specific risk factors associated with regional disparities in obesity prevalence.

Across all socioeconomic factors, our findings illustrated that social gradients had a greater influence on female adolescents. Sex differences in socioeconomic inequality of obesity were globally reported in adult populations, but not observed in children in most countries [9,12]. In Korea, a higher risk of obesity was demonstrated in both female adults and adolescents with a lower socioeconomic background [31]. Few studies have elucidated the direct cause of the higher vulnerability of female students to socioeconomic inequality, although it is known that female students engage in less physical activity and more sedentary behavior than male students [32]. A possible explanation is that low SEP has a greater impact on health behaviors in females, or that it mediates between obesity and women’s biological factors. For instance, girls at low SEP are likely to have an earlier onset of menarche, which may increase the risk of obesity via its hormonal and metabolic effects [33].

Similarly, middle school students had a greater vulnerability to social gradients compared to high school students. It could be, at least partly, explained by younger children being more dependent on their nurturing environment. Young adolescents are more prone to poorer health behaviors when they receive less parental supervision and guidance, which often occurs in dual-income families. Studies on the association between maternal work hours and childhood obesity have found that children whose mothers work are more likely to be sedentary and consume low-quality food [25,34]. Prior research also reported a negative correlation between the father’s education level and the offspring’s obesity, although the correlation weakened as the offspring became older [35].

Our study has some strengths: the KYRBS is conducted annually nationwide with a high response rate–approximately 95.8%, and it includes various socioeconomic information. This allowed us to obtain a representative sample of Korean adolescents and to analyze the trend of obesity according to socioeconomic status. To our knowledge, this is the first study to highlight secular trends in socioeconomic inequalities in obesity among Korean adolescents using recent multiyear data. The limitations of this study should also be considered. First, height and weight were self-reported and were not measured. Previous studies on the accuracy of self-reported height and weight in assessing obesity pointed out biased BMI values as a concern: specifically, adolescents underreport weight and overreport height, which results in an underestimation of their BMI [36]. This implies that the actual prevalence of obesity may have been higher than that observed in our study. We additionally analyzed the prevalence of adolescent obesity using measured data from the Korea National Health and Nutrition Examination Survey from 2007 to 2020. These figures were higher than the prevalence from self-reported data (Supplementary Material 11). Nonetheless, the increasing trend in prevalence persisted in our additional analysis. Moreover, a previous study measured the height and weight of the 2008 KYRBS participants, and compared them with self-reported data. It found that the sensitivity of obesity was 69% and the specificity was 100%, while the kappa value was 0.79 (95% CI, 0.70 to 0.88) [37]. As the KYRBS collected self-reported data on all occasions, the trend of obesity is reliable while recognizing the chance of underestimation. Second, although the data were collected via national sampling, the results may not be extrapolated to the general population of Korean adolescents. We could not include youths outside of school in this study, and they are likely socioeconomically marginalized. Last, we used a Korean growth reference. This might hinder comparison at the global level. In the 2017 KNGC, the BMI values at the 95th percentile during the transition from adolescence to adulthood converged at approximately 25 kg/m^2^, whereas the corresponding values from the WHO and CDC met at around 30 kg/m^2^. Nonetheless, there has been debate regarding the use of a BMI cut-off of 30 kg/m^2^ to define obesity in the Asian population [38]. Using the higher cut-off may hinder screening for obesity in Asian populations and underestimate their related health risks [39]. In this context, it seems appropriate to use the Korean growth standard to evaluate the growth and development of adolescents.

This study illustrated increasing trends in the prevalence of obesity and growing socioeconomic inequalities in obesity among Korean youth. The widening socioeconomic gap resulted from the greater increase in obesity among the low-SEP group than among the high-SEP group. To tackle both the increasing prevalence and widening inequality, relevant policies and interventions should target disadvantaged girls and middle school students as well as boys and high school students.

## Figures and Tables

**Figure f3-epih-45-e2023033:**
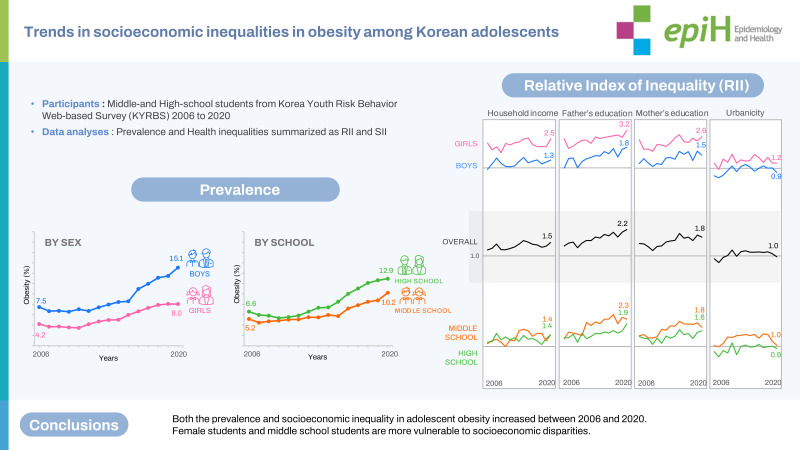


**Table 1.** General characteristics of the study population from 2006 to 2020

Characteristics			Year^1^															Annual change	p for trend^2^
			2006	2007	2008	2009	2010	2011	2012	2013	2014	2015	2016	2017	2018	2019	2020		
Total (n)			57,511	60,817	61,804	60,363	57,749	61,732	59,724	56,849	55,934	52,166	50,310	47,241	45,291	47,095	43,624		
Age, mean (yr)			15.07	15.12	15.12	15.21	15.24	15.28	15.08	15.11	15.15	15.22	15.25	15.26	15.29	15.19	15.31	-0.001	0.021
Subgroups																			
	Sex																		
		Boys	51.2	51.5	51.9	52.0	52.0	51.6	51.6	51.4	50.9	51.1	51.3	51.2	51.1	52.1	51.6	0.000	0.646
		Girls	48.8	48.5	48.1	48.0	48.0	48.4	48.4	48.6	49.1	48.9	48.7	48.8	48.9	47.9	48.4	0.000	0.646
	Stage																		
		High school	49.2	51.1	51.3	52.7	53.3	53.7	54.5	55.4	55.6	56.5	58.3	58.1	56.9	54.7	53.6	0.001	<0.001
		Middle school	50.8	48.9	48.7	47.3	46.7	46.3	45.5	44.6	44.4	43.5	41.7	41.9	43.1	45.3	46.4	-0.001	<0.001
Socioeconomic factors																			
	Household income																		
		High	32.6	29.9	30.4	30.0	31.8	32.3	32.4	34.3	35.9	38.8	39.7	43.0	44.1	41.1	42.0	0.012	<0.001
		Middle	47.5	47.2	47.1	47.1	46.4	46.4	46.3	46.5	47.2	45.5	46.0	44.0	44.1	47.2	46.1	-0.002	<0.001
		Low	19.9	22.9	22.5	23.0	21.8	21.3	21.4	19.2	16.9	15.6	14.3	12.9	11.8	11.7	11.8	-0.010	<0.001
	Father's education																		
		Tertiary or above	45.7	47.2	49.3	51.0	53.3	54.8	55.1	56.7	60.3	63.0	63.8	66.3	68.7	69.9	71.7	0.011	<0.001
		Upper secondary	45.6	45.3	43.7	42.5	40.8	40.2	40.4	39.4	36.4	34.2	33.8	31.5	29.6	28.3	26.7	-0.018	<0.001
		Basic or less	8.7	7.5	7.0	6.5	5.9	5.0	4.5	3.9	3.3	2.8	2.4	2.1	1.7	1.8	1.6	-0.007	<0.001
	Mother's education																		
		Tertiary or above	30.3	31.1	34.3	36.8	39.2	41.6	43.1	45.9	50.5	54.4	56.4	59.8	63.5	65.8	67.8	0.021	<0.001
		Upper secondary	59.2	60.0	58.3	56.6	54.8	53.3	52.4	50.4	46.6	43.1	41.6	38.4	35.1	32.8	31.1	-0.026	<0.001
		Basic or less	10.5	8.9	7.4	6.6	6.0	5.1	4.5	3.8	2.8	2.4	2.1	1.8	1.5	1.4	1.1	-0.008	<0.001
	Urbanicity																		
		Metropolitan cities	47.2	46.6	55.2	54.7	45.7	45.3	44.8	44.7	43.9	44.3	43.7	43.9	43.7	43.2	42.9	-0.005	<0.001
		Other cities	45.2	47.1	39.6	40.2	48.1	48.7	49.3	48.6	49.9	49.8	50.8	50.3	50.7	51.5	51.5	0.010	<0.001
		Rural areas	7.6	6.3	5.2	5.1	6.2	5.9	6.0	6.7	6.2	5.9	5.4	5.8	5.6	5.3	5.5	-0.006	<0.001

Values are presented as %.

1 The corresponding standard deviation and 95% confidence interval are presented in the supplementary document.

2 The p-value for trend was tested for each row of the table.

**Table 2.** Self-reported anthropometric measures and overall prevalence of obesity defined by various growth references

Variabes				Year															Annual change	p for trend
				2006	2007	2008	2009	2010	2011	2012	2013	2014	2015	2016	2017	2018	2019	2020		
Height (cm)																				
	Total			165.1	165.4	165.4	165.7	165.6	165.7	165.1	165.2	165.3	165.6	165.7	165.9	166.1	166.0	166.6	0.057	<0.001
	Sex																			
		Boys		169.8	170.1	170.1	170.6	170.6	170.8	170.0	170.2	170.4	170.7	170.9	171.1	171.3	171.1	171.8	0.090	<0.001
		Girls		160.2	160.4	160.3	160.3	160.3	160.2	160.0	160.0	160.0	160.3	160.3	160.4	160.5	160.5	161.0	0.024	<0.001
	Stage																			
		High school		167.6	167.8	167.8	168.0	168.0	167.9	167.5	167.5	167.5	167.5	167.6	167.8	167.9	168.1	168.5	0.026	<0.001
		Middle school		162.7	162.8	162.9	163.0	162.9	163.1	162.3	162.4	162.6	163.2	163.1	163.3	163.6	163.5	164.4	0.082	<0.001
Weight (kg)																				
	Total			56.4	56.5	56.4	56.6	56.6	56.8	56.6	57.0	57.1	57.7	58.4	58.8	59.3	59.3	60.1	0.243	<0.001
	Sex																			
		Boys		60.8	60.8	60.8	61.2	61.4	61.3	60.9	61.3	61.7	62.2	63.3	64.1	64.7	64.7	66.1	0.327	<0.001
		Girls		51.9	51.8	51.7	51.6	51.5	52.0	52.0	52.5	52.4	53.0	53.3	53.4	53.6	53.5	53.6	0.156	<0.001
	Stage																			
		High school		59.8	59.6	59.4	59.5	59.5	59.6	59.5	59.9	60.1	60.5	61.2	61.7	62.2	62.5	62.9	0.250	<0.001
		Middle school		53.2	53.1	53.3	53.3	53.3	53.5	53.1	53.4	53.5	54.0	54.5	54.9	55.4	55.4	56.9	0.223	<0.001
BMI (kg/m^2^)																				
	Total			20.6	20.5	20.5	20.5	20.5	20.6	20.6	20.8	20.8	20.9	21.1	21.3	21.4	21.4	21.5	0.072	<0.001
	Sex																			
		Boys		21.0	20.9	20.9	20.9	21.0	20.9	21.0	21.1	21.1	21.2	21.6	21.8	21.9	22.0	22.3	0.089	<0.001
		Girls		20.2	20.1	20.1	20.0	20.0	20.2	20.3	20.5	20.4	20.6	20.7	20.7	20.8	20.7	20.6	0.054	<0.001
	Stage																			
		High school		21.2	21.1	21.0	21.0	21.0	21.1	21.1	21.3	21.3	21.5	21.7	21.8	21.9	22.0	22.0	0.080	<0.001
		Middle school		20.0	20.0	20.0	19.9	20.0	20.0	20.1	20.1	20.1	20.2	20.4	20.5	20.6	20.6	20.9	0.061	<0.001
Overall obesity (%)																				
	BMI ≥95th percentile (2017 KNGC)																			
		Total		5.9	5.2	5.3	5.1	5.3	5.5	6.1	6.5	6.8	7.3	8.8	9.7	10.6	10.9	11.7	0.005	<0.001
		Sex																		
			Boys	7.5	6.7	6.7	6.6	7.0	6.7	7.4	7.9	8.4	8.6	10.9	12.0	13.1	13.5	15.1	0.006	<0.001
			Girls	4.2	3.7	3.7	3.5	3.4	4.2	4.7	5.0	5.0	5.9	6.6	7.3	7.9	8.1	8.0	0.004	<0.001
		Stage																		
			High school	6.6	6.0	5.8	5.4	5.6	5.8	6.6	7.3	7.4	8.5	10.0	11.0	12.1	12.6	12.9	0.006	<0.001
			Middle school	5.2	4.5	4.7	4.9	5.1	5.1	5.5	5.5	5.9	5.7	7.2	7.8	8.5	8.8	10.2	0.004	<0.001
	BMI ≥95th percentile (WHO-GR)																			
		Total		6.3	5.8	5.9	5.6	5.9	5.8	6.5	7.0	7.1	7.5	8.9	9.6	10.6	11.2	12.2	0.005	<0.001
		Sex																		
			Boys	10.3	9.6	9.6	9.3	9.8	9.3	10.4	11.1	11.4	11.5	13.8	14.7	16.4	17.0	18.9	0.007	<0.001
			Girls	2.1	1.7	1.9	1.7	1.6	2.1	2.5	2.7	2.6	3.3	3.7	4.2	4.5	4.9	5.1	0.003	<0.001
		Stage																		
			High school	4.5	4.2	4.0	3.8	3.9	4.0	4.6	5.3	5.3	6.1	7.6	8.3	9.1	9.8	9.9	0.005	<0.001
			Middle school	8.0	7.4	7.9	7.7	8.1	7.9	8.9	9.2	9.3	9.2	10.7	11.4	12.6	12.9	14.9	0.005	<0.001
	BMI>median+2SD (WHO-GR)																			
		Total		2.7	2.4	2.6	2.4	2.6	2.4	2.9	3.2	3.4	3.5	4.5	4.9	5.6	6.1	6.7	0.003	<0.001
		Sex																		
			Boys	5.1	4.5	4.7	4.4	4.8	4.2	5.1	5.5	6.1	6.0	7.8	8.3	9.5	10.0	11.1	0.005	<0.001
			Girls	0.3	0.2	0.2	0.2	0.2	0.3	0.4	0.6	0.6	0.8	1.1	1.3	1.5	1.8	2.0	0.001	<0.001
		Stage																		
			High school	1.7	1.7	1.6	1.3	1.5	1.3	1.7	2.2	2.4	2.7	3.7	4.0	4.7	5.3	5.1	0.003	<0.001
			Middle school	3.7	3.2	3.6	3.6	3.9	3.6	4.2	4.3	4.7	4.5	5.7	6.2	6.7	7.0	8.5	0.003	<0.001

BMI, body mass index; 2017 KNGC, the 2017 Korean National Growth Charts; WHO-GR, the World Health Organization Growth Reference; SD, standard deviations.

## SUPPLEMENTARY MATERIALS

Supplementary Material 1. General characteristics with standard deviation for weighted mean and 95% confidence intervals for weighted percentage

Supplementary Material 2. Self-reported anthropometric measures and overall prevalence of obesity with standard deviation for weighted mean and 95% confidence intervals for weighted percentage

Supplementary Material 3. Obesity prevalence by socioeconomic status from 2006 to 2020 (among the total study group)

Supplementary Material 4. Prevalence ratio and 95% confidence intervals by socioeconomic status from 2006 to 2020 (among the total study group)

Supplementary Material 5. Relative Index of Inequality (RII) based on the prevalence ratio from 2006 to 2020

Supplementary Material 6. Odds ratio and 95% confidence intervals by socioeconomic status from 2006 to 2020 (among the total study group)

Supplementary Material 7. Relative Index of Inequality (RII) based on the odds ratio from 2006 to 2020

Supplementary Material 8. Slope Index of Inequality (SII) based on the prevalence difference from 2006 to 2020

Supplementary Material 9. Prevalence of stunting according to socioeconomic status

Supplementary Material 10. Prevalence of the coexistence of stunting and obesity according to socioeconomic status

Supplementary Material 11. Prevalence of adolescent obesity based on physical measurement data in KNHANES
