# Supplementary material for: Trends in socioeconomic inequalities in obesity among Korean adolescents: the Korea Youth Risk Behavior Web-based Survey (KYRBS) 2006 to 2020
Source: Epidemiol Health. 2023 Mar 7;45:e2023033. doi: 10.4178/epih.e2023033 (PMC10586920; doi:10.4178/epih.e2023033)
Supplement: Supplementary Material 1. — General characteristics with standard deviation for weighted mean and 95% confidence intervals for weighted percentage [file epih-45-e2023033-Supplementary-1.docx]

| **Supplementary Material 1. General characteristics with standard deviation for weighted mean and 95% confidence intervals for weighted percentage** | | | | | | | | | | | | | | | | |
| --- | --- | --- | --- | --- | --- | --- | --- | --- | --- | --- | --- | --- | --- | --- | --- | --- |
|  | | **Year** | | | | | | | | | | | | | | |
|  | | **2006** | **2007** | **2008** | **2009** | **2010** | **2011** | **2012** | **2013** | **2014** | **2015** | **2016** | **2017** | **2018** | **2019** | **2020** |
| N = | | 57,511 | 60,817 | 61,804 | 60,363 | 57,749 | 61,732 | 59,724 | 56,849 | 55,934 | 52,166 | 50,310 | 47,241 | 45,291 | 47,095 | 43,624 |
| Age, mean (year) | | 15.07±0.03 | 15.12±0.03 | 15.12±0.02 | 15.21±0.03 | 15.24±0.03 | 15.28±0.02 | 15.08±0.02 | 15.11±0.02 | 15.15±0.02 | 15.22±0.02 | 15.25±0.02 | 15.26±0.02 | 15.29±0.02 | 15.19±0.02 | 15.31±0.02 |
| **Subgroups** | |  |  |  |  |  |  |  |  |  |  |  |  |  |  |  |
| Sex (%) | |  |  |  |  |  |  |  |  |  |  |  |  |  |  |  |
|  | Boys | 51.2  (48.2-54.2) | 51.5  (48.4-54.6) | 51.9  (49-54.8) | 52  (48.9-55) | 52  (48.7-55.4) | 51.6  (49-54.2) | 51.6  (48.7-54.5) | 51.4  (48.5-54.4) | 50.9  (48.1-53.7) | 51.1  (48.1-54) | 51.3  (48.3-54.2) | 51.2  (48.3-54.1) | 51.1  (48.3-53.9) | 52.1  (49.4-54.8) | 51.6  (49.1-54.2) |
|  | Girls | 48.8  (45.8-51.8) | 48.5  (45.4-51.6) | 48.1  (45.2-51) | 48  (45-51.1) | 48  (44.6-51.3) | 48.4  (45.8-51) | 48.4  (45.5-51.3) | 48.6  (45.6-51.5) | 49.1  (46.3-51.9) | 48.9  (46-51.9) | 48.7  (45.8-51.7) | 48.8  (45.9-51.7) | 48.9  (46.1-51.7) | 47.9  (45.2-50.6) | 48.4  (45.8-50.9) |
| Stage (%) | |  |  |  |  |  |  |  |  |  |  |  |  |  |  |  |
|  | High school | 49.2  (47.4-51) | 51.1  (49.2-52.9) | 51.3  (49.7-52.9) | 52.7  (50.9-54.5) | 53.3  (51.4-55.2) | 53.7  (52.3-55.1) | 54.5  (53-56.1) | 55.4  (53.8-57.1) | 55.6  (54-57.1) | 56.5  (54.9-58.2) | 58.3  (56.7-59.8) | 58.1  (56.4-59.8) | 56.9  (55.2-58.6) | 54.7  (53-56.3) | 53.6  (51.9-55.3) |
|  | Middle school | 50.8  (49-52.6) | 48.9  (47.1-50.8) | 48.7  (47.1-50.3) | 47.3  (45.5-49.1) | 46.7  (44.8-48.6) | 46.3  (44.9-47.7) | 45.5  (43.9-47) | 44.6  (42.9-46.2) | 44.4  (42.9-46) | 43.5  (41.8-45.1) | 41.7  (40.2-43.3) | 41.9  (40.2-43.6) | 43.1  (41.4-44.8) | 45.3  (43.7-47) | 46.4  (44.7-48.1) |
| **Socioeconomic factors** | |  |  |  |  |  |  |  |  |  |  |  |  |  |  |  |
| Household income (%) | |  |  |  |  |  |  |  |  |  |  |  |  |  |  |  |
|  | High | 32.6  (31.7-33.4) | 29.9  (29.1-30.8) | 30.4  (29.7-31.2) | 30.0  (29.1-30.9) | 31.8  (30.9-32.7) | 32.3  (31.5-33.1) | 32.4  (31.6-33.1) | 34.3  (33.5-35.1) | 35.9  (35.1-36.7) | 38.8  (38.0-39.6) | 39.7  (38.9-40.5) | 43.0  (42.1-43.9) | 44.1  (43.3-44.9) | 41.1  (40.3-41.9) | 42.0  (41.2-42.9) |
|  | Middle | 47.5  (46.8-48.2) | 47.2  (46.6-47.7) | 47.1  (46.5-47.6) | 47.1  (46.4-47.7) | 46.4  (45.7-47) | 46.4  (45.9-47.0) | 46.3  (45.7-46.8) | 46.5  (45.9-47.0) | 47.2  (46.6-47.8) | 45.5  (44.9-46.1) | 46  (45.4-46.6) | 44.0  (43.3-44.7) | 44.1  (43.5-44.7) | 47.2  (46.6-47.8) | 46.1  (45.4-46.8) |
|  | Low | 19.9  (19.3-20.5) | 22.9  (22.2-23.5) | 22.5  (21.9-23.1) | 23.0  (22.3-23.6) | 21.8  (21.2-22.5) | 21.3  (20.7-21.8) | 21.4  (20.8-21.9) | 19.2  (18.7-19.8) | 16.9  (16.4-17.4) | 15.6  (15.2-16.1) | 14.3  (13.9-14.7) | 12.9  (12.5-13.4) | 11.8  (11.4-12.2) | 11.7  (11.3-12.1) | 11.8  (11.4-12.2) |
| Father's education (%) | |  |  |  |  |  |  |  |  |  |  |  |  |  |  |  |
|  | Tertiary or above | 45.7  (44.2-47.2) | 47.2  (45.8-48.6) | 49.3  (48-50.6) | 51.0  (49.7-52.3) | 53.3  (52-54.7) | 54.8  (53.7-56) | 55.1  (54-56.2) | 56.7  (55.6-57.8) | 60.3  (59.3-61.4) | 63.0  (61.9-64.1) | 63.8  (62.7-64.8) | 66.3  (65.2-67.4) | 68.7  (67.7-69.7) | 69.9  (68.9-71.0) | 71.7  (70.7-72.8) |
|  | Upper secondary | 45.6  (44.4-46.9) | 45.3  (44.1-46.5) | 43.7  (42.6-44.7) | 42.5  (41.3-43.7) | 40.8  (39.6-42) | 40.2  (39.2-41.2) | 40.4  (39.4-41.4) | 39.4  (38.4-40.4) | 36.4  (35.4-37.4) | 34.2  (33.2-35.2) | 33.8  (32.8-34.8) | 31.5  (30.5-32.6) | 29.6  (28.6-30.6) | 28.3  (27.3-29.3) | 26.7  (25.7-27.7) |
|  | Basic or less | 8.7  (8.2-9.1) | 7.5  (7.1-7.9) | 7.0  (6.7-7.4) | 6.5  (6.2-6.8) | 5.9  (5.5-6.2) | 5.0  (4.7-5.2) | 4.5  (4.2-4.7) | 3.9  (3.7-4.2) | 3.3  (3.1-3.4) | 2.8  (2.6-3.0) | 2.4  (2.3-2.6) | 2.1  (2.0-2.3) | 1.7  (1.6-1.8) | 1.8  (1.6-1.9) | 1.6  (1.4-1.8) |
| Mother's education (%) | |  |  |  |  |  |  |  |  |  |  |  |  |  |  |  |
|  | Tertiary or above | 30.3  (29.0-31.6) | 31.1  (29.8-32.4) | 34.3  (33.0-35.5) | 36.8  (35.5-38.2) | 39.2  (37.7-40.6) | 41.6  (40.4-42.7) | 43.1  (42.0-44.2) | 45.9  (44.8-47.0) | 50.5  (49.5-51.6) | 54.4  (53.3-55.6) | 56.4  (55.3-57.5) | 59.8  (58.6-60.9) | 63.5  (62.4-64.5) | 65.8  (64.7-66.9) | 67.8  (66.7-68.9) |
|  | Upper secondary | 59.2  (58.1-60.3) | 60.0  (58.9-61.1) | 58.3  (57.3-59.3) | 56.6  (55.4-57.8) | 54.8  (53.5-56.1) | 53.3  (52.3-54.4) | 52.4  (51.4-53.4) | 50.4  (49.3-51.4) | 46.6  (45.6-47.6) | 43.1  (42.1-44.2) | 41.6  (40.5-42.6) | 38.4  (37.3-39.5) | 35.1  (34.1-36.1) | 32.8  (31.7-33.8) | 31.1  (30.0-32.1) |
|  | Basic or less | 10.5  (10.0-11.0) | 8.9  (8.5-9.3) | 7.4  (7.1-7.8) | 6.6  (6.2-6.9) | 6.0  (5.7-6.3) | 5.1  (4.8-5.3) | 4.5  (4.3-4.8) | 3.8  (3.6-4.0) | 2.8  (2.7-3.0) | 2.4  (2.3-2.6) | 2.1  (1.9-2.2) | 1.8  (1.6-1.9) | 1.5  (1.3-1.6) | 1.4  (1.3-1.6) | 1.1  (1.0-1.3) |
| Urbanicity (%) | |  |  |  |  |  |  |  |  |  |  |  |  |  |  |  |
|  | Metropolitan cities | 47.2  (45.6-48.9) | 46.6  (44.9-48.2) | 55.2  (53.6-56.8) | 54.7  (52.9-56.5) | 45.7  (44.0-47.4) | 45.3  (44.1-46.6) | 44.8  (43.4-46.1) | 44.7  (43.2-46.2) | 43.9  (42.5-45.2) | 44.3  (42.8-45.8) | 43.7  (42.3-45.1) | 43.9  (42.4-45.4) | 43.7  (42.2-45.1) | 43.2  (41.8-44.6) | 42.9  (41.4-44.4) |
|  | Other cities | 45.2  (43.1-47.2) | 47.1  (45.3-48.9) | 39.6  (37.9-41.3) | 40.2  (38.3-42.0) | 48.1  (46.1-50.0) | 48.7  (47.2-50.3) | 49.3  (47.7-50.9) | 48.6  (46.9-50.4) | 49.9  (48.3-51.6) | 49.8  (48.1-51.5) | 50.8  (49.3-52.4) | 50.3  (48.6-52.0) | 50.7  (49.1-52.3) | 51.5  (49.9-53.1) | 51.5  (49.9-53.2) |
|  | Rural areas | 7.6  (6.3-8.9) | 6.3  (5.7-7.0) | 5.2  (4.6-5.8) | 5.1  (4.6-5.7) | 6.2  (5.3-7.2) | 5.9  (5.1-6.8) | 6.0  (5.2-6.8) | 6.7  (5.6-7.7) | 6.2  (5.3-7.1) | 5.9  (4.9-6.8) | 5.4  (4.6-6.3) | 5.8  (4.7-6.9) | 5.6  (4.6-6.7) | 5.3  (4.5-6.1) | 5.5  (4.7-6.4) |
| Age was presented as weighted mean (year) with standard deviation, and others were as weighted percentage (%) with 95% confidence interval. | | | | | | | | | | | | | | | | |
