# Supplementary material for: Trends in socioeconomic inequalities in obesity among Korean adolescents: the Korea Youth Risk Behavior Web-based Survey (KYRBS) 2006 to 2020
Source: Epidemiol Health. 2023 Mar 7;45:e2023033. doi: 10.4178/epih.e2023033 (PMC10586920; doi:10.4178/epih.e2023033)
Supplement: Supplementary Material 2. — Self-reported anthropometric measures and overall prevalence of obesity with standard deviation for weighted mean and 95% confidence intervals for weighted percentage [file epih-45-e2023033-Supplementary-2.docx]

| **Supplementary Material 2. Self-reported anthropometric measures and overall prevalence of obesity with standard deviation for weighted mean and 95% confidence intervals for weighted percentage** | | | | | | | | | | | | | | | | | |
| --- | --- | --- | --- | --- | --- | --- | --- | --- | --- | --- | --- | --- | --- | --- | --- | --- | --- |
|  |  |  |  |  |  |  |  |  |  | **Year** |  |  |  |  |  |  |  |
|  |  |  | **2006** | **2007** | **2008** | **2009** | **2010** | **2011** | **2012** | **2013** | **2014** | **2015** | **2016** | **2017** | **2018** | **2019** | **2020** |
| **Height (cm)** | | |  |  |  |  |  |  |  |  |  |  |  |  |  |  |  |
|  | Total |  | 165.11 ±0.16 | 165.38 ±0.16 | 165.42 ±0.16 | 165.66 ±0.18 | 165.63 ±0.19 | 165.66 ±0.15 | 165.12 ±0.16 | 165.25 ±0.17 | 165.33 ±0.15 | 165.61 ±0.17 | 165.75 ±0.17 | 165.88 ±0.17 | 166.06 ±0.17 | 166.03 ±0.16 | 166.58 ±0.15 |
|  | Sex | Boys | 169.77 ±0.12 | 170.09 ±0.11 | 170.13 ±0.1 | 170.57 ±0.11 | 170.59 ±0.11 | 170.76 ±0.09 | 169.96 ±0.11 | 170.19 ±0.11 | 170.44 ±0.1 | 170.74 ±0.1 | 170.92 ±0.1 | 171.13 ±0.1 | 171.33 ±0.1 | 171.09 ±0.1 | 171.8 ±0.09 |
|  |  | Girls | 160.22 ±0.05 | 160.38 ±0.05 | 160.32 ±0.05 | 160.35 ±0.05 | 160.25 ±0.05 | 160.24 ±0.04 | 159.96 ±0.04 | 160.01 ±0.05 | 160.03 ±0.04 | 160.26 ±0.04 | 160.31 ±0.04 | 160.38 ±0.04 | 160.55 ±0.04 | 160.54 ±0.04 | 161.01 ±0.04 |
|  | Stage | High school | 167.61 ±0.29 | 167.84 ±0.29 | 167.79 ±0.28 | 168.04 ±0.3 | 167.99 ±0.33 | 167.85 ±0.25 | 167.48 ±0.27 | 167.53 ±0.27 | 167.51 ±0.25 | 167.47 ±0.28 | 167.61 ±0.28 | 167.76 ±0.28 | 167.95 ±0.27 | 168.12 ±0.26 | 168.46 ±0.26 |
|  |  | Middle school | 162.69 ±0.12 | 162.82 ±0.12 | 162.92 ±0.12 | 163.02 ±0.13 | 162.93 ±0.14 | 163.13 ±0.11 | 162.29 ±0.12 | 162.41 ±0.12 | 162.6 ±0.12 | 163.18 ±0.11 | 163.15 ±0.12 | 163.28 ±0.11 | 163.57 ±0.12 | 163.52 ±0.12 | 164.41 ±0.12 |
| **Weight (kg)** | | |  |  |  |  |  |  |  |  |  |  |  |  |  |  |  |
|  | Total |  | 56.43 ±0.16 | 56.45 ±0.16 | 56.42 ±0.16 | 56.56 ±0.18 | 56.63 ±0.19 | 56.78 ±0.15 | 56.57 ±0.16 | 57.02 ±0.17 | 57.14 ±0.15 | 57.69 ±0.16 | 58.40 ±0.18 | 58.84 ±0.19 | 59.25 ±0.19 | 59.32 ±0.18 | 60.07 ±0.19 |
|  | Sex | Boys | 60.79 ±0.15 | 60.83 ±0.13 | 60.81 ±0.12 | 61.17 ±0.14 | 61.35 ±0.13 | 61.30 ±0.12 | 60.86 ±0.14 | 61.32 ±0.15 | 61.69 ±0.13 | 62.15 ±0.14 | 63.27 ±0.16 | 64.05 ±0.16 | 64.66 ±0.15 | 64.68 ±0.15 | 66.12 ±0.14 |
|  |  | Girls | 51.86 ±0.09 | 51.80 ±0.08 | 51.67 ±0.07 | 51.57 ±0.09 | 51.52 ±0.09 | 51.97 ±0.07 | 52.00 ±0.08 | 52.46 ±0.07 | 52.41 ±0.08 | 53.03 ±0.08 | 53.27 ±0.08 | 53.38 ±0.09 | 53.59 ±0.09 | 53.50 ±0.09 | 53.60 ±0.09 |
|  | Stage | High school | 59.75 ±0.28 | 59.63 ±0.28 | 59.38 ±0.27 | 59.50 ±0.28 | 59.53 ±0.31 | 59.57 ±0.24 | 59.48 ±0.25 | 59.93 ±0.26 | 60.05 ±0.23 | 60.49 ±0.26 | 61.20 ±0.28 | 61.72 ±0.29 | 62.17 ±0.28 | 62.54 ±0.28 | 62.85 ±0.29 |
|  |  | Middle school | 53.23 ±0.12 | 53.14 ±0.12 | 53.29 ±0.12 | 53.28 ±0.14 | 53.32 ±0.15 | 53.54 ±0.12 | 53.08 ±0.12 | 53.39 ±0.12 | 53.49 ±0.12 | 54.04 ±0.12 | 54.48 ±0.13 | 54.85 ±0.14 | 55.40 ±0.15 | 55.44 ±0.15 | 56.86 ±0.16 |
| **BMI (kg/m2)** | | |  |  |  |  |  |  |  |  |  |  |  |  |  |  |  |
|  | Total |  | 20.58 ±0.02 | 20.53 ±0.02 | 20.50 ±0.02 | 20.49 ±0.02 | 20.52 ±0.03 | 20.58 ±0.02 | 20.63 ±0.02 | 20.77 ±0.02 | 20.79 ±0.02 | 20.92 ±0.02 | 21.13 ±0.03 | 21.25 ±0.03 | 21.35 ±0.03 | 21.37 ±0.03 | 21.50 ±0.03 |
|  | Sex | Boys | 20.98 ±0.03 | 20.92 ±0.03 | 20.91 ±0.02 | 20.93 ±0.03 | 20.99 ±0.03 | 20.93 ±0.03 | 20.96 ±0.03 | 21.06 ±0.03 | 21.13 ±0.03 | 21.22 ±0.03 | 21.55 ±0.04 | 21.76 ±0.04 | 21.92 ±0.03 | 21.98 ±0.03 | 22.31 ±0.03 |
|  |  | Girls | 20.16 ±0.03 | 20.11 ±0.02 | 20.07 ±0.02 | 20.02 ±0.03 | 20.03 ±0.03 | 20.21 ±0.02 | 20.29 ±0.03 | 20.45 ±0.02 | 20.43 ±0.03 | 20.61 ±0.03 | 20.69 ±0.03 | 20.72 ±0.03 | 20.75 ±0.03 | 20.72 ±0.03 | 20.64 ±0.03 |
|  | Stage | High school | 21.17 ±0.03 | 21.08 ±0.03 | 21.00 ±0.03 | 20.98 ±0.03 | 21.00 ±0.03 | 21.06 ±0.03 | 21.12 ±0.03 | 21.26 ±0.03 | 21.31 ±0.03 | 21.48 ±0.03 | 21.68 ±0.03 | 21.82 ±0.04 | 21.93 ±0.04 | 22.01 ±0.04 | 22.02 ±0.04 |
|  |  | Middle school | 20.01 ±0.02 | 19.95 ±0.03 | 19.98 ±0.02 | 19.94 ±0.03 | 19.98 ±0.03 | 20.03 ±0.03 | 20.05 ±0.03 | 20.14 ±0.03 | 20.13 ±0.03 | 20.20 ±0.03 | 20.36 ±0.03 | 20.46 ±0.03 | 20.59 ±0.03 | 20.61 ±0.03 | 20.90 ±0.04 |
| **Overall Obesity (%)** | | |  |  |  |  |  |  |  |  |  |  |  |  |  |  |  |
| **BMI ≥95th percentile**  **(2017 KNGC)** | | |  |  |  |  |  |  |  |  |  |  |  |  |  |  |  |
|  | Total |  | 5.9  (5.6-6.1) | 5.2  (5.0-5.5) | 5.3  (5.0-5.5) | 5.1  (4.9-5.4) | 5.3  (5.1-5.6) | 5.5  (5.3-5.7) | 6.1  (5.9-6.3) | 6.5  (6.3-6.7) | 6.8  (6.5-7.0) | 7.3  (7.0-7.6) | 8.8  (8.5-9.2) | 9.7  (9.4-10.0) | 10.6  (10.2-10.9) | 10.9  (10.5-11.2) | 11.7  (11.3-12.1) |
|  | Sex | Boys | 7.5  (7.1-7.8) | 6.7  (6.3-7.1) | 6.7  (6.4-7.1) | 6.6  (6.2-6.9) | 7.0  (6.7-7.4) | 6.7  (6.4-7.0) | 7.4  (7.1-7.8) | 7.9  (7.6-8.3) | 8.4  (8.1-8.8) | 8.6  (8.2-9.0) | 10.9  (10.5-11.4) | 12.0  (11.5-12.4) | 13.1  (12.6-13.6) | 13.5  (13.0-13.9) | 15.1  (14.5-15.6) |
|  |  | Girls | 4.2  (3.8-4.6) | 3.7  (3.4-4.0) | 3.7  (3.4-4.0) | 3.5  (3.2-3.8) | 3.4  (3.2-3.7) | 4.2  (3.9-4.4) | 4.7  (4.4-5.0) | 5.0  (4.7-5.3) | 5.0  (4.7-5.3) | 5.9  (5.6-6.3) | 6.6  (6.3-7.0) | 7.3  (6.9-7.7) | 7.9  (7.4-8.3) | 8.1  (7.7-8.5) | 8.0  (7.6-8.5) |
|  | Stage | High school | 6.6  (6.2-7.0) | 6.0  (5.6-6.3) | 5.8  (5.4-6.2) | 5.4  (5.0-5.7) | 5.6  (5.2-5.9) | 5.8  (5.5-6.2) | 6.6  (6.2-6.9) | 7.3  (7.0-7.7) | 7.4  (7.1-7.8) | 8.5  (8.1-8.9) | 10.0  (9.6-10.5) | 11.0  (10.6-11.5) | 12.1  (11.6-12.6) | 12.6  (12.1-13.2) | 12.9  (12.4-13.5) |
|  |  | Middle school | 5.2  (4.8-5.5) | 4.5  (4.1-4.8) | 4.7  (4.4-5.0) | 4.9  (4.5-5.2) | 5.1  (4.7-5.4) | 5.1  (4.8-5.4) | 5.5  (5.2-5.8) | 5.5  (5.2-5.8) | 5.9  (5.6-6.3) | 5.7  (5.4-6.1) | 7.2  (6.8-7.6) | 7.8  (7.4-8.3) | 8.5  (8.0-9.0) | 8.8  (8.3-9.2) | 10.2  (9.7-10.8) |
| **BMI ≥95th percentile**  **(WHO-GR)** | | |  |  |  |  |  |  |  |  |  |  |  |  |  |  |  |
|  | Total |  | 6.3  (6.0-6.6) | 5.8  (5.4-6.1) | 5.9  (5.6-6.2) | 5.6  (5.3-6.0) | 5.9  (5.5-6.2) | 5.8  (5.5-6.1) | 6.5  (6.2-6.8) | 7.0  (6.7-7.3) | 7.1  (6.8-7.4) | 7.5  (7.2-7.8) | 8.9  (8.5-9.3) | 9.6  (9.2-10.0) | 10.6  (10.2-11.0) | 11.2  (10.8-11.6) | 12.2  (11.7-12.7) |
|  | Sex | Boys | 10.3  (9.9-10.7) | 9.6  (9.2-10.1) | 9.6  (9.2-10.1) | 9.3  (8.9-9.7) | 9.8  (9.4-10.3) | 9.3  (8.9-9.7) | 10.4  (9.9-10.8) | 11.1  (10.7-11.5) | 11.4  (10.9-11.8) | 11.5  (11.1-11.9) | 13.8  (13.3-14.2) | 14.7  (14.2-15.3) | 16.4  (15.8-16.9) | 17.0  (16.5-17.6) | 18.9  (18.3-19.5) |
|  |  | Girls | 2.1  (1.8-2.3) | 1.7  (1.5-1.9) | 1.9  (1.7-2.0) | 1.7  (1.5-1.9) | 1.6  (1.4-1.8) | 2.1  (1.9-2.3) | 2.5  (2.3-2.7) | 2.7  (2.5-2.9) | 2.6  (2.4-2.8) | 3.3  (3.1-3.5) | 3.7  (3.4-4.0) | 4.2  (3.9-4.5) | 4.5  (4.2-4.8) | 4.9  (4.6-5.2) | 5.1  (4.8-5.4) |
|  | Stage | High school | 4.5  (4.1-4.9) | 4.2  (3.8-4.6) | 4.0  (3.6-4.4) | 3.8  (3.4-4.1) | 3.9  (3.5-4.3) | 4.0  (3.6-4.3) | 4.6  (4.2-5.0) | 5.3  (4.9-5.7) | 5.3  (4.9-5.7) | 6.1  (5.7-6.6) | 7.6  (7.1-8.1) | 8.3  (7.8-8.8) | 9.1  (8.5-9.6) | 9.8  (9.2-10.3) | 9.9  (9.3-10.5) |
|  |  | Middle school | 8.0  (7.6-8.5) | 7.4  (6.9-8.0) | 7.9  (7.4-8.3) | 7.7  (7.2-8.3) | 8.1  (7.5-8.7) | 7.9  (7.5-8.4) | 8.9  (8.4-9.4) | 9.2  (8.7-9.7) | 9.3  (8.7-9.8) | 9.2  (8.7-9.8) | 10.7  (10.1-11.3) | 11.4  (10.8-12) | 12.6  (11.9-13.2) | 12.9  (12.2-13.6) | 14.9  (14.1-15.6) |
| **BMI >median+2SD**  **(WHO-GR)** | | |  |  |  |  |  |  |  |  |  |  |  |  |  |  |  |
|  | Total |  | 2.7  (2.5-2.9) | 2.4  (2.2-2.6) | 2.6  (2.4-2.7) | 2.4  (2.2-2.6) | 2.6  (2.4-2.8) | 2.4  (2.2-2.5) | 2.9  (2.7-3.0) | 3.2  (3.0-3.3) | 3.4  (3.2-3.6) | 3.5  (3.3-3.7) | 4.5  (4.2-4.8) | 4.9  (4.6-5.2) | 5.6  (5.3-5.9) | 6.1  (5.8-6.4) | 6.7  (6.3-7.0) |
|  | Sex | Boys | 5.1  (4.7-5.4) | 4.5  (4.2-4.8) | 4.7  (4.4-5.0) | 4.4  (4.1-4.8) | 4.8  (4.5-5.1) | 4.2  (4.0-4.5) | 5.1  (4.8-5.4) | 5.5  (5.3-5.8) | 6.1  (5.8-6.4) | 6.0  (5.7-6.4) | 7.8  (7.4-8.1) | 8.3  (8.0-8.7) | 9.5  (9.1-9.9) | 10.0  (9.6-10.4) | 11.1  (10.6-11.6) |
|  |  | Girls | 0.3  (0.2-0.3) | 0.2  (0.2-0.3) | 0.2  (0.2-0.3) | 0.2  (0.1-0.2) | 0.2  (0.2-0.3) | 0.3  (0.3-0.4) | 0.4  (0.4-0.5) | 0.6  (0.5-0.7) | 0.6  (0.5-0.7) | 0.8  (0.7-0.9) | 1.1  (1.0-1.2) | 1.3  (1.2-1.4) | 1.5  (1.4-1.7) | 1.8  (1.7-2.0) | 2.0  (1.8-2.2) |
|  | Stage | High school | 1.7  (1.5-2.0) | 1.7  (1.5-1.9) | 1.6  (1.4-1.8) | 1.3  (1.1-1.5) | 1.5  (1.3-1.7) | 1.3  (1.1-1.4) | 1.7  (1.5-1.9) | 2.2  (2.0-2.4) | 2.4  (2.2-2.7) | 2.7  (2.4-2.9) | 3.7  (3.3-4.1) | 4.0  (3.6-4.3) | 4.7  (4.4-5.1) | 5.3  (4.9-5.8) | 5.1  (4.7-5.5) |
|  |  | Middle school | 3.7  (3.4-4.0) | 3.2  (2.8-3.5) | 3.6  (3.3-3.9) | 3.6  (3.2-4.0) | 3.9  (3.5-4.3) | 3.6  (3.3-3.9) | 4.2  (3.9-4.6) | 4.3  (4.0-4.7) | 4.7  (4.3-5.0) | 4.5  (4.2-4.9) | 5.7  (5.2-6.1) | 6.2  (5.7-6.6) | 6.7  (6.3-7.2) | 7.0  (6.6-7.5) | 8.5  (7.9-9.0) |
| BMI, body mass index; 2017 KNGC, the 2017 Korean National Growth Charts; WHOGS, the World Health Organization Growth Standards; SD, standard deviations. | | | | | | | | | | | | | | | | | |
