# Supplementary material for: Trends in socioeconomic inequalities in obesity among Korean adolescents: the Korea Youth Risk Behavior Web-based Survey (KYRBS) 2006 to 2020
Source: Epidemiol Health. 2023 Mar 7;45:e2023033. doi: 10.4178/epih.e2023033 (PMC10586920; doi:10.4178/epih.e2023033)
Supplement: Supplementary Material 3. — Obesity prevalence by socioeconomic status from 2006 to 2020 (among the total study group) [file epih-45-e2023033-Supplementary-3.docx]

| **Supplementary Material 3. Obesity prevalence by socioeconomic status from 2006 to 2020 (among the total study group)** | | | | | | | | | | | | | | | |  |
| --- | --- | --- | --- | --- | --- | --- | --- | --- | --- | --- | --- | --- | --- | --- | --- | --- |
|  |  |  |  |  |  |  |  | **Year** |  |  |  |  |  |  |  |  |
|  | **2006** | **2007** | **2008** | **2009** | **2010** | **2011** | **2012** | **2013** | **2014** | **2015** | **2016** | **2017** | **2018** | **2019** | **2020** | ***P for trend*** |
| **Total** |  |  |  |  |  |  |  |  |  |  |  |  |  |  |  |  |
| **Household income (%)** |  |  |  |  |  |  |  |  |  |  |  |  |  |  |  |  |
| High | 1038  (5.9) | 894  (5.0) | 908  (5.1) | 907  (5.1) | 980  (5.3) | 1057  (5.3) | 1151  (6.1) | 1151  (6.0) | 1220  (6.1) | 1342  (6.6) | 1656  (8.3) | 1870  (9.2) | 1989  (10.1) | 2038  (10.4) | 1986  (10.7) | *<0.001* |
| Middle | 1488  (5.4) | 1503  (5.0) | 1446  (4.7) | 1371  (4.7) | 1351  (4.9) | 1501  (5.1) | 1512  (5.4) | 1704  (6.3) | 1712  (6.5) | 1731  (7.1) | 1990  (8.4) | 1948  (9.2) | 2052  (10.3) | 2332  (10.3) | 2426  (11.5) | *<0.001* |
| Low | 823  (7.0) | 936  (6.0) | 974  (6.8) | 903  (6.0) | 840  (6.3) | 871  (6.5) | 977  (7.7) | 919  (8.0) | 843  (9.1) | 793  (9.5) | 852  (11.7) | 821  (13.2) | 742  (13.4) | 849  (14.9) | 861  (15.9) | *<0.001* |
| **Father’s education (%)** |  |  |  |  |  |  |  |  |  |  |  |  |  |  |  |  |
| Tertiary or above | 1309  (5.4) | 1214  (4.7) | 1273  (4.6) | 1333  (4.8) | 1386  (4.8) | 1512  (4.9) | 1655  (5.4) | 1670  (5.6) | 1858  (6.0) | 1943  (6.3) | 2360  (7.8) | 2488  (8.4) | 2763  (9.5) | 1841  (9.7) | 2244  (10.2) | *<0.001* |
| Upper secondary | 1645  (6.2) | 1502  (5.5) | 1507  (5.8) | 1398  (5.2) | 1390  (5.8) | 1510  (6.0) | 1581  (6.7) | 1686  (7.4) | 1558  (7.7) | 1569  (8.8) | 1754  (10.4) | 1753  (11.7) | 1661  (12.6) | 1118  (13.5) | 1327  (14.9) | *<0.001* |
| Basic or less | 395  (6.7) | 347  (6.5) | 291  (5.9) | 288  (6.3) | 255  (6.5) | 244  (7.4) | 241  (8.4) | 227  (9.0) | 183  (9.8) | 149  (9.1) | 168  (12.9) | 160  (15.6) | 115  (13.8) | 90  (17.2) | 98  (17.4) | *<0.001* |
| **Mother’s education (%)** |  |  |  |  |  |  |  |  |  |  |  |  |  |  |  |  |
| Tertiary or above | 819  (5.0) | 806  (4.6) | 896  (4.8) | 985  (5.0) | 1022  (4.9) | 1193  (5.0) | 1294  (5.4) | 1380  (5.7) | 1561  (5.9) | 1651  (6.1) | 2123  (7.8) | 2329  (8.5) | 2610  (9.6) | 1781  (9.7) | 2206  (10.5) | *<0.001* |
| Upper secondary | 2069  (6.1) | 1892  (5.3) | 1895  (5.3) | 1807  (5.1) | 1773  (5.4) | 1847  (5.6) | 1982  (6.5) | 2031  (7.1) | 1896  (7.5) | 1887  (8.5) | 2059  (10.0) | 1976  (11.0) | 1884  (12.0) | 1212  (13.0) | 1445  (13.9) | *<0.001* |
| Basic or less | 461  (6.8) | 413  (6.6) | 309  (6.3) | 267  (6.3) | 250  (6.8) | 246  (7.3) | 229  (8.4) | 191  (8.3) | 142  (8.7) | 152  (11.1) | 139  (13.4) | 129  (15.4) | 88  (12.6) | 75  (17.2) | 65  (15.4) | *<0.001* |
| **Urbanicity (%)** |  |  |  |  |  |  |  |  |  |  |  |  |  |  |  |  |
| Metropolitan cities | 1662  (6.0) | 1623  (5.6) | 1689  (5.3) | 1644  (5.1) | 1422  (5.1) | 1572  (5.5) | 1621  (5.9) | 1615  (6.2) | 1635  (6.6) | 1650  (7.2) | 1944  (8.7) | 2020  (9.6) | 2085  (10.3) | 2318  (10.9) | 2312  (11.9) | *<0.001* |
| Other cities | 1224  (5.7) | 1247  (4.9) | 1236  (5.2) | 1158  (5.1) | 1255  (5.3) | 1425  (5.4) | 1580  (6.2) | 1685  (6.7) | 1793  (6.7) | 1838  (7.2) | 2153  (8.7) | 2209  (9.5) | 2286  (10.4) | 2440  (10.6) | 2511  (11.3) | *<0.001* |
| Rural areas | 463  (5.8) | 463  (5.5) | 403  (5.3) | 379  (5.3) | 494  (6.5) | 432  (5.6) | 439  (7.0) | 474  (7.0) | 347  (8.1) | 378  (9.1) | 401  (11.4) | 410  (12.0) | 412  (13.2) | 461  (13.8) | 450  (13.3) | *<0.001* |
|  |  |  |  |  |  |  |  |  |  |  |  |  |  |  |  |  |

| **Supplementary Material 3 *(continued)*. Obesity prevalence by socioeconomic status from 2006 to 2020 (according to sex)** | | | | | | | | | | | | | | | |  |
| --- | --- | --- | --- | --- | --- | --- | --- | --- | --- | --- | --- | --- | --- | --- | --- | --- |
|  |  |  |  |  |  |  |  | **Year** |  |  |  |  |  |  |  |  |
|  | **2006** | **2007** | **2008** | **2009** | **2010** | **2011** | **2012** | **2013** | **2014** | **2015** | **2016** | **2017** | **2018** | **2019** | **2020** | ***P for trend*** |
| **Boys** |  |  |  |  |  |  |  |  |  |  |  |  |  |  |  |  |
| **Household income (%)** |  |  |  |  |  |  |  |  |  |  |  |  |  |  |  |  |
| High | 756  (7.8) | 643  (6.5) | 659  (6.5) | 657  (6.5) | 748  (7.1) | 741  (6.8) | 802  (7.7) | 805  (7.6) | 853  (7.9) | 919  (8.4) | 1150  (10.6) | 1281  (11.5) | 1357  (12.8) | 1432  (13.2) | 1436  (14.4) | *<0.001* |
| Middle | 924  (7.1) | 977  (6.5) | 928  (6.2) | 876  (6.2) | 891  (6.8) | 867  (6.5) | 887  (6.7) | 967  (7.7) | 1005  (8.2) | 958  (8.2) | 1177  (10.5) | 1122  (11.4) | 1233  (13.0) | 1422  (12.9) | 1536  (14.9) | *<0.001* |
| Low | 449  (7.7) | 564  (7.2) | 572  (8.3) | 544  (7.4) | 503  (7.4) | 441  (7.1) | 519  (8.5) | 475  (9.1) | 471  (10.4) | 414  (10.1) | 487  (13.3) | 451  (15.3) | 393  (14.6) | 496  (16.8) | 515  (18.2) | *<0.001* |
| **Father’s education (%)** |  |  |  |  |  |  |  |  |  |  |  |  |  |  |  |  |
| Tertiary or above | 912  (7.4) | 822  (6.3) | 874  (6.1) | 927  (6.5) | 973  (6.7) | 987  (6.3) | 1057  (6.9) | 1082  (7.3) | 1219  (7.8) | 1227  (7.7) | 1560  (10.1) | 1600  (10.7) | 1822  (12.4) | 1132  (13.0) | 1470  (14.2) | *<0.001* |
| Upper secondary | 995  (7.6) | 952  (6.9) | 939  (7.3) | 872  (6.5) | 930  (7.7) | 836  (7.1) | 928  (8.0) | 944  (8.7) | 901  (9.1) | 881  (10.1) | 1024  (12.2) | 1031  (14.1) | 955  (14.6) | 625  (16.9) | 760  (18.9) | *<0.001* |
| Basic or less | 222  (7.0) | 227  (8.4) | 180  (7.2) | 162  (6.8) | 148  (6.5) | 130  (7.9) | 128  (8.9) | 120  (9.8) | 107  (11.3) | 75  (8.9) | 107  (15.3) | 85  (16.2) | 61  (14.4) | 52  (19.3) | 53  (20.7) | *<0.001* |
| **Mother’s education (%)** |  |  |  |  |  |  |  |  |  |  |  |  |  |  |  |  |
| Tertiary or above | 575  (6.9) | 557  (6.1) | 635  (6.4) | 699  (6.7) | 733  (6.7) | 795  (6.6) | 841  (6.8) | 911  (7.3) | 1054  (7.8) | 1070  (7.6) | 1402  (10.0) | 1499  (10.7) | 1735  (12.5) | 1101  (13.1) | 1456  (14.6) | *<0.001* |
| Upper secondary | 1300  (7.8) | 1212  (6.9) | 1178  (6.7) | 1125  (6.5) | 1168  (7.2) | 1042  (6.8) | 1161  (7.9) | 1125  (8.4) | 1096  (9.1) | 1037  (9.7) | 1211  (12.1) | 1168  (13.6) | 1065  (14.0) | 667  (16.4) | 813  (17.6) | *<0.001* |
| Basic or less | 254  (7.2) | 256  (7.8) | 185  (7.6) | 169  (7.9) | 148  (7.6) | 132  (7.8) | 121  (8.8) | 110  (9.7) | 66  (8.6) | 75  (11.9) | 84  (15.6) | 58  (16.2) | 45  (14.2) | 44  (21.4) | 37  (18.4) | *<0.001* |
| **Urbanicity (%)** |  |  |  |  |  |  |  |  |  |  |  |  |  |  |  |  |
| Metropolitan cities | 1101  (7.9) | 1083  (7.3) | 1145  (7.0) | 1121  (6.7) | 1018  (7.0) | 972  (6.9) | 1032  (7.3) | 979  (7.6) | 1027  (8.5) | 1028  (8.9) | 1249  (11.1) | 1225  (11.9) | 1340  (13.3) | 1524  (13.6) | 1597  (15.7) | *<0.001* |
| Other cities | 770  (7.2) | 798  (6.1) | 768  (6.4) | 738  (6.5) | 805  (7.0) | 830  (6.6) | 928  (7.5) | 988  (8.3) | 1084  (8.2) | 1043  (8.1) | 1333  (10.6) | 1388  (11.8) | 1395  (12.8) | 1559  (13.2) | 1609  (14.4) | *<0.001* |
| Rural areas | 258  (6.5) | 303  (6.8) | 246  (6.2) | 218  (6.0) | 319  (8.0) | 247  (6.3) | 248  (7.5) | 280  (7.7) | 218  (9.9) | 220  (10.0) | 232  (12.8) | 241  (14.1) | 248  (15.2) | 267  (15.2) | 281  (15.9) | *<0.001* |
| **Girls** |  |  |  |  |  |  |  |  |  |  |  |  |  |  |  |  |
| **Household income (%)** |  |  |  |  |  |  |  |  |  |  |  |  |  |  |  |  |
| High | 282  (3.5) | 251  (3.1) | 249  (3.2) | 250  (3.3) | 232  (2.9) | 316  (3.4) | 349  (3.9) | 346  (3.8) | 367  (3.8) | 423  (4.4) | 506  (5.5) | 589  (6.3) | 632  (6.8) | 606  (7.0) | 550  (6.3) | *<0.001* |
| Middle | 564  (3.7) | 526  (3.5) | 518  (3.3) | 495  (3.2) | 460  (3.0) | 634  (3.8) | 625  (4.1) | 737  (4.9) | 707  (4.8) | 773  (6.1) | 813  (6.6) | 826  (7.1) | 819  (7.7) | 910  (7.7) | 890  (8.2) | *<0.001* |
| Low | 374  (6.3) | 372  (4.7) | 402  (5.2) | 359  (4.6) | 337  (5.1) | 430  (5.9) | 458  (7.0) | 444  (7.0) | 372  (7.8) | 379  (8.9) | 365  (10.0) | 370  (11.1) | 349  (12.3) | 353  (12.9) | 346  (13.3) | *<0.001* |
| **Father’s education (%)** |  |  |  |  |  |  |  |  |  |  |  |  |  |  |  |  |
| Tertiary or above | 397  (3.2) | 392  (3.0) | 399  (2.9) | 406  (2.9) | 413  (2.8) | 525  (3.3) | 598  (3.9) | 588  (3.9) | 639  (4.0) | 716  (4.7) | 800  (5.3) | 888  (6.0) | 941  (6.4) | 709  (6.9) | 774  (6.6) | *<0.001* |
| Upper secondary | 650  (4.8) | 550  (4.1) | 568  (4.3) | 526  (3.9) | 460  (3.8) | 674  (4.9) | 653  (5.4) | 742  (6.1) | 657  (6.2) | 688  (7.5) | 730  (8.6) | 722  (9.3) | 706  (10.5) | 493  (10.7) | 567  (11.6) | *<0.001* |
| Basic or less | 173  (6.4) | 120  (4.4) | 111  (4.3) | 126  (5.8) | 107  (6.5) | 114  (6.7) | 113  (7.9) | 107  (8.1) | 76  (8.1) | 74  (9.4) | 61  (10.1) | 75  (15.1) | 54  (13.0) | 38  (15.2) | 45  (14.6) | *<0.001* |
| **Mother’s education (%)** |  |  |  |  |  |  |  |  |  |  |  |  |  |  |  |  |
| Tertiary or above | 244  (3.0) | 249  (3.1) | 261  (3.0) | 286  (3.0) | 289  (2.8) | 398  (3.3) | 453  (3.9) | 469  (3.8) | 507  (3.8) | 581  (4.4) | 721  (5.3) | 830  (6.0) | 875  (6.4) | 680  (6.8) | 750  (6.8) | *<0.001* |
| Upper secondary | 769  (4.4) | 680  (3.7) | 717  (3.9) | 682  (3.7) | 605  (3.6) | 805  (4.5) | 821  (5.0) | 906  (5.8) | 800  (5.9) | 850  (7.4) | 848  (8.0) | 808  (8.5) | 819  (10.1) | 545  (10.2) | 632  (10.9) | *<0.001* |
| Basic or less | 207  (6.3) | 157  (5.4) | 124  (4.9) | 98  (4.8) | 102  (5.9) | 114  (6.9) | 108  (8.0) | 81  (6.6) | 76  (8.8) | 77  (10.3) | 55  (11.0) | 71  (14.8) | 43  (11.0) | 31  (13.8) | 28  (12.7) | *<0.001* |
| **Urbanicity (%)** |  |  |  |  |  |  |  |  |  |  |  |  |  |  |  |  |
| Metropolitan cities | 561  (4.1) | 540  (3.8) | 544  (3.5) | 523  (3.4) | 404  (3.1) | 600  (4.0) | 589  (4.4) | 636  (4.7) | 608  (4.7) | 622  (5.3) | 695  (6.2) | 795  (7.1) | 745  (7.3) | 794  (8.0) | 715  (7.8) | *<0.001* |
| Other cities | 454  (4.2) | 449  (3.6) | 468  (4.0) | 420  (3.6) | 450  (3.6) | 595  (4.2) | 652  (4.7) | 697  (5.1) | 709  (5.1) | 795  (6.3) | 820  (6.7) | 821  (7.2) | 891  (8.0) | 881  (7.8) | 902  (8.0) | *<0.001* |
| Rural areas | 205  (5.1) | 160  (4.0) | 157  (4.2) | 161  (4.5) | 175  (4.6) | 185  (4.9) | 191  (6.4) | 194  (6.1) | 129  (6.2) | 158  (8.0) | 169  (9.9) | 169  (10.0) | 164  (11) | 194  (12.2) | 169  (10.4) | *<0.001* |
|  |  |  |  |  |  |  |  |  |  |  |  |  |  |  |  |  |

| **Supplementary Material 3 *(continued)*. Obesity prevalence by socioeconomic status from 2006 to 2020 (according to school stage)** | | | | | | | | | | | | | | | |  |
| --- | --- | --- | --- | --- | --- | --- | --- | --- | --- | --- | --- | --- | --- | --- | --- | --- |
|  |  |  |  |  |  |  |  | **Year** |  |  |  |  |  |  |  |  |
|  | **2006** | **2007** | **2008** | **2009** | **2010** | **2011** | **2012** | **2013** | **2014** | **2015** | **2016** | **2017** | **2018** | **2019** | **2020** | ***P for trend*** |
| **High school** |  |  |  |  |  |  |  |  |  |  |  |  |  |  |  |  |
| **Household income (%)** |  |  |  |  |  |  |  |  |  |  |  |  |  |  |  |  |
| High | 479  (7.0) | 446  (6.0) | 418  (5.7) | 412  (5.4) | 422  (5.2) | 492  (5.9) | 547  (6.7) | 591  (7.3) | 610  (7.0) | 738  (8.3) | 890  (9.7) | 1032  (11.2) | 1126  (12.4) | 1075  (12.4) | 972  (12.1) | *<0.001* |
| Middle | 856  (5.9) | 882  (5.6) | 809  (5.0) | 747  (4.9) | 770  (5.2) | 826  (5.2) | 855  (5.7) | 1012  (6.9) | 1021  (6.9) | 1068  (7.9) | 1281  (9.5) | 1207  (9.9) | 1292  (11.3) | 1464  (11.8) | 1419  (12.5) | *<0.001* |
| Low | 571  (7.6) | 627  (6.6) | 651  (7.4) | 570  (6.2) | 545  (6.5) | 602  (6.8) | 648  (8.0) | 632  (8.3) | 585  (9.1) | 578  (10.2) | 608  (12.1) | 602  (14.0) | 518  (14.1) | 592  (16.2) | 561  (16.7) | *<0.001* |
| **Father’s education (%)** |  |  |  |  |  |  |  |  |  |  |  |  |  |  |  |  |
| Tertiary or above | 695  (6.1) | 671  (5.4) | 685  (5.1) | 702  (5.2) | 738  (5.0) | 803  (5.5) | 926  (6.2) | 966  (6.6) | 1058  (6.8) | 1156  (7.5) | 1424  (9.2) | 1462  (9.9) | 1649  (11.2) | 1030  (12.0) | 1126  (11.6) | *<0.001* |
| Upper secondary | 940  (6.8) | 892  (6.2) | 862  (6.3) | 753  (5.1) | 769  (6.0) | 858  (6.0) | 873  (6.8) | 998  (8.0) | 936  (8.0) | 1002  (9.6) | 1113  (11.1) | 1123  (12.3) | 1067  (13.7) | 688  (14.7) | 776  (15.9) | *<0.001* |
| Basic or less | 271  (7.5) | 223  (6.6) | 189  (6.3) | 177  (6.6) | 166  (7.0) | 156  (7.1) | 156  (9.0) | 147  (9.1) | 121  (9.8) | 94  (8.8) | 108  (12.7) | 108  (15.8) | 80  (14.3) | 60  (18.3) | 60  (19.1) | *<0.001* |
| **Mother’s education (%)** |  |  |  |  |  |  |  |  |  |  |  |  |  |  |  |  |
| Tertiary or above | 406  (5.6) | 411  (5.5) | 456  (5.4) | 504  (5.5) | 523  (5.2) | 617  (5.9) | 665  (6.0) | 787  (6.8) | 855  (6.7) | 942  (7.4) | 1237  (9.2) | 1323  (10.0) | 1532  (11.5) | 980  (12.0) | 1088  (11.9) | *<0.001* |
| Upper secondary | 1184  (6.9) | 1135  (6.0) | 1076  (5.6) | 995  (5.2) | 991  (5.6) | 1061  (5.6) | 1172  (6.8) | 1218  (7.6) | 1156  (7.7) | 1221  (9.3) | 1337  (10.8) | 1312  (11.9) | 1220  (12.8) | 761  (14.4) | 854  (15.0) | *<0.001* |
| Basic or less | 316  (7.1) | 290  (6.9) | 221  (6.9) | 170  (6.6) | 166  (7.0) | 170  (7.7) | 136  (8.3) | 133  (8.8) | 100  (9.1) | 114  (12.1) | 94  (13.6) | 84  (15.0) | 63  (13.6) | 50  (19.5) | 36  (14.3) | *<0.001* |
| **Urbanicity (%)** |  |  |  |  |  |  |  |  |  |  |  |  |  |  |  |  |
| Metropolitan cities | 960  (6.9) | 966  (6.5) | 962  (6.0) | 915  (5.6) | 780  (5.5) | 935  (6.3) | 964  (6.6) | 991  (7.3) | 1009  (7.5) | 1037  (8.5) | 1236  (10.0) | 1295  (11.2) | 1329  (12.2) | 1425  (12.8) | 1305  (13.2) | *<0.001* |
| Other cities | 695  (6.4) | 713  (5.4) | 705  (5.5) | 616  (5.1) | 677  (5.4) | 771  (5.4) | 877  (6.6) | 994  (7.4) | 1017  (7.2) | 1146  (8.5) | 1313  (9.8) | 1304  (10.8) | 1393  (11.9) | 1420  (12.1) | 1392  (12.7) | *<0.001* |
| Rural areas | 251  (6.1) | 276  (5.9) | 211  (5.9) | 198  (5.0) | 280  (7.0) | 214  (5.6) | 209  (6.6) | 250  (7.4) | 190  (8.2) | 201  (8.5) | 230  (12.4) | 242  (11.7) | 214  (13.4) | 286  (15.5) | 255  (13.0) | *<0.001* |
| **Middle school** |  |  |  |  |  |  |  |  |  |  |  |  |  |  |  |  |
| **Household income (%)** |  |  |  |  |  |  |  |  |  |  |  |  |  |  |  |  |
| High | 559  (5.2) | 448  (4.3) | 490  (4.6) | 495  (4.9) | 558  (5.4) | 565  (4.9) | 604  (5.5) | 560  (4.9) | 610  (5.3) | 604  (5.1) | 766  (6.9) | 838  (7.2) | 863  (7.8) | 963  (8.7) | 1014  (9.6) | *<0.001* |
| Middle | 632  (4.9) | 621  (4.4) | 637  (4.4) | 624  (4.4) | 581  (4.5) | 675  (5.0) | 657  (4.9) | 692  (5.5) | 691  (5.8) | 663  (6.0) | 709  (6.7) | 741  (8.0) | 760  (8.7) | 868  (8.2) | 1007  (10.1) | *<0.001* |
| Low | 252  (6.0) | 309  (5.0) | 323  (5.8) | 333  (5.8) | 295  (5.9) | 269  (5.9) | 329  (7.1) | 287  (7.5) | 258  (9.1) | 215  (7.8) | 244  (10.7) | 219  (10.9) | 224  (11.9) | 257  (12.2) | 300  (14.4) | *<0.001* |
| **Father’s education (%)** |  |  |  |  |  |  |  |  |  |  |  |  |  |  |  |  |
| Tertiary or above | 614  (4.8) | 543  (4.0) | 588  (4.2) | 631  (4.4) | 648  (4.6) | 709  (4.3) | 729  (4.7) | 704  (4.6) | 800  (5.1) | 787  (4.9) | 936  (6.1) | 1026  (6.6) | 1114  (7.5) | 811  (7.6) | 1118  (9.0) | *<0.001* |
| Upper secondary | 705  (5.5) | 610  (4.7) | 645  (5.2) | 645  (5.3) | 621  (5.5) | 652  (6.1) | 708  (6.6) | 688  (6.6) | 622  (7.1) | 567  (7.3) | 641  (9.1) | 630  (10.5) | 594  (10.6) | 430  (11.7) | 551  (13.6) | *<0.001* |
| Basic or less | 124  (5.4) | 124  (6.4) | 102  (5.1) | 111  (5.8) | 89  (5.6) | 88  (8.0) | 85  (7.3) | 80  (8.8) | 62  (10.0) | 55  (9.9) | 60  (13.3) | 52  (15.2) | 35  (12.6) | 30  (14.8) | 38  (15.0) | *<0.001* |
| **Mother’s education (%)** |  |  |  |  |  |  |  |  |  |  |  |  |  |  |  |  |
| Tertiary or above | 413  (4.6) | 395  (4.0) | 440  (4.3) | 481  (4.5) | 499  (4.6) | 576  (4.4) | 629  (4.9) | 593  (4.6) | 706  (5.1) | 709  (4.8) | 886  (6.2) | 1006  (6.8) | 1078  (7.5) | 801  (7.7) | 1118  (9.4) | *<0.001* |
| Upper secondary | 885  (5.4) | 757  (4.5) | 819  (5.0) | 812  (5.1) | 782  (5.2) | 786  (5.7) | 810  (6.0) | 813  (6.3) | 740  (7.0) | 666  (7.1) | 722  (8.6) | 664  (9.3) | 664  (10.6) | 451  (10.9) | 591  (12.3) | *<0.001* |
| Basic or less | 145  (6.1) | 123  (6.1) | 88  (5.0) | 97  (5.9) | 84  (6.5) | 76  (6.6) | 93  (8.8) | 58  (7.0) | 42  (7.8) | 38  (8.5) | 45  (12.9) | 45  (16.5) | 25  (10.2) | 25  (13.0) | 29  (16.8) | *<0.001* |
| **Urbanicity (%)** |  |  |  |  |  |  |  |  |  |  |  |  |  |  |  |  |
| Metropolitan cities | 702  (5.2) | 657  (4.5) | 727  (4.6) | 729  (4.5) | 642  (4.7) | 637  (4.6) | 657  (5.1) | 624  (4.9) | 626  (5.5) | 613  (5.4) | 708  (6.8) | 725  (7.2) | 756  (7.9) | 893  (8.5) | 1007  (10.4) | *<0.001* |
| Other cities | 529  (5.1) | 534  (4.4) | 531  (4.9) | 542  (5.2) | 578  (5.3) | 654  (5.4) | 703  (5.7) | 691  (5.8) | 776  (6.1) | 692  (5.6) | 840  (7.2) | 905  (7.9) | 893  (8.5) | 1020  (8.7) | 1119  (9.8) | *<0.001* |
| Rural areas | 212  (5.4) | 187  (4.9) | 192  (4.6) | 181  (5.5) | 214  (5.7) | 218  (5.6) | 230  (7.5) | 224  (6.4) | 157  (8.1) | 177  (10.0) | 171  (10.2) | 168  (12.7) | 198  (12.9) | 175  (11.4) | 195  (13.6) | *<0.001* |
|  |  |  |  |  |  |  |  |  |  |  |  |  |  |  |  |  |
