# Supplementary material for: Trends in socioeconomic inequalities in obesity among Korean adolescents: the Korea Youth Risk Behavior Web-based Survey (KYRBS) 2006 to 2020
Source: Epidemiol Health. 2023 Mar 7;45:e2023033. doi: 10.4178/epih.e2023033 (PMC10586920; doi:10.4178/epih.e2023033)
Supplement: Supplementary Material 4. — Prevalence ratio and 95% confidence intervals by socioeconomic status from 2006 to 2020 (among the total study group) [file epih-45-e2023033-Supplementary-4.docx]

| **Supplementary Material 4*.* Prevalence ratio and 95% confidence intervals by socioeconomic status from 2006 to 2020 (among the total study group)** | | | | | | | | | | | | | | | |  |
| --- | --- | --- | --- | --- | --- | --- | --- | --- | --- | --- | --- | --- | --- | --- | --- | --- |
|  |  |  |  |  |  |  |  | **Year** |  |  |  |  |  |  |  |  |
|  | **2006** | **2007** | **2008** | **2009** | **2010** | **2011** | **2012** | **2013** | **2014** | **2015** | **2016** | **2017** | **2018** | **2019** | **2020** | **P for trend** |
| **Total** |  |  |  |  |  |  |  |  |  |  |  |  |  |  |  |  |
| **Household income** |  |  |  |  |  |  |  |  |  |  |  |  |  |  |  |  |
| High | 1.00 |  |  |  |  |  |  |  |  |  |  |  |  |  |  |  |
| Middle | 0.92  (0.99-0.85) | 1  (1.08-0.92) | 0.97  (1.05-0.9) | 0.91  (0.99-0.84) | 0.91  (0.98-0.84) | 0.94  (1.02-0.88) | 0.89  (0.96-0.83) | 1.06  (1.14-0.98) | 1.05  (1.13-0.98) | 1.09  (1.17-1.02) | 1.04  (1.1-0.97) | 1  (1.07-0.95) | 1.02  (1.08-0.96) | 0.98  (1.04-0.93) | 1.08  (1.14-1.02) | 0.001 |
| Low | 1.15  (1.26-1.05) | 1.21  (1.33-1.11) | 1.33  (1.45-1.22) | 1.18  (1.3-1.08) | 1.16  (1.26-1.06) | 1.18  (1.28-1.08) | 1.24  (1.35-1.15) | 1.33  (1.45-1.22) | 1.45  (1.58-1.34) | 1.45  (1.57-1.33) | 1.43  (1.54-1.32) | 1.42  (1.53-1.31) | 1.36  (1.47-1.26) | 1.41  (1.52-1.31) | 1.44  (1.55-1.34) | <0.001 |
| **Father’s education** |  |  |  |  |  |  |  |  |  |  |  |  |  |  |  |  |
| Tertiary or above | 1.00 |  |  |  |  |  |  |  |  |  |  |  |  |  |  |  |
| Upper secondary | 1.08  (1.16-1.01) | 1.13  (1.21-1.05) | 1.18  (1.27-1.1) | 1.11  (1.19-1.03) | 1.16  (1.25-1.08) | 1.23  (1.32-1.15) | 1.22  (1.3-1.14) | 1.33  (1.42-1.25) | 1.33  (1.42-1.25) | 1.39  (1.49-1.31) | 1.33  (1.41-1.25) | 1.42  (1.5-1.34) | 1.34  (1.42-1.26) | 1.42  (1.52-1.32) | 1.46  (1.55-1.37) | <0.001 |
| Basic or less | 1.18  (1.32-1.06) | 1.37  (1.53-1.22) | 1.23  (1.4-1.09) | 1.3  (1.47-1.15) | 1.33  (1.51-1.17) | 1.46  (1.67-1.28) | 1.54  (1.75-1.35) | 1.65  (1.88-1.44) | 1.67  (1.93-1.45) | 1.58  (1.85-1.35) | 1.7  (1.97-1.47) | 1.85  (2.14-1.6) | 1.53  (1.81-1.28) | 1.75  (2.12-1.44) | 1.67  (2.01-1.39) | 0.001 |
| **Mother’s education** |  |  |  |  |  |  |  |  |  |  |  |  |  |  |  |  |
| Tertiary or above | 1.00 |  |  |  |  |  |  |  |  |  |  |  |  |  |  |  |
| Upper secondary | 1.11  (1.2-1.03) | 1.08  (1.17-1) | 1.13  (1.22-1.05) | 1.08  (1.17-1) | 1.13  (1.21-1.04) | 1.11  (1.2-1.04) | 1.2  (1.29-1.12) | 1.27  (1.35-1.18) | 1.28  (1.37-1.2) | 1.39  (1.49-1.31) | 1.28  (1.35-1.2) | 1.29  (1.37-1.22) | 1.28  (1.35-1.21) | 1.33  (1.42-1.24) | 1.34  (1.42-1.26) | <0.001 |
| Basic or less | 1.19  (1.33-1.06) | 1.36  (1.53-1.21) | 1.23  (1.39-1.09) | 1.19  (1.36-1.04) | 1.3  (1.49-1.14) | 1.4  (1.6-1.23) | 1.5  (1.71-1.31) | 1.45  (1.68-1.25) | 1.5  (1.77-1.27) | 1.92  (2.24-1.64) | 1.66  (1.94-1.41) | 1.72  (2.03-1.46) | 1.39  (1.69-1.14) | 1.76  (2.18-1.43) | 1.46  (1.83-1.17) | 0.216 |
| **Urbanicity** |  |  |  |  |  |  |  |  |  |  |  |  |  |  |  |  |
| Metropolitan cities | 1.00 |  |  |  |  |  |  |  |  |  |  |  |  |  |  |  |
| Other cities | 0.95  (1.02-0.88) | 0.96  (1.03-0.89) | 1.09  (1.17-1.02) | 1.05  (1.13-0.98) | 1.08  (1.16-1.01) | 1.02  (1.1-0.95) | 1.03  (1.1-0.96) | 1.12  (1.2-1.05) | 1.04  (1.11-0.98) | 1.06  (1.13-1) | 1.04  (1.1-0.98) | 1.05  (1.11-0.99) | 1.05  (1.11-0.99) | 1  (1.05-0.94) | 0.97  (1.02-0.92) | 0.479 |
| Rural areas | 0.95  (1.05-0.86) | 1.02  (1.13-0.92) | 1.03  (1.14-0.92) | 1.01  (1.12-0.9) | 1.23  (1.36-1.11) | 1.02  (1.13-0.92) | 1.14  (1.26-1.03) | 1.14  (1.26-1.03) | 1.23  (1.38-1.1) | 1.33  (1.48-1.2) | 1.36  (1.5-1.23) | 1.27  (1.4-1.15) | 1.3  (1.43-1.17) | 1.22  (1.34-1.11) | 1.13  (1.24-1.03) | <0.001 |
|  |  |  |  |  |  |  |  |  |  |  |  |  |  |  |  |  |

| **Supplementary Material 4 *(continued)*. Prevalence ratio and 95% confidence intervals by socioeconomic status from 2006 to 2020 (according to sex)** | | | | | | | | | | | | | | | | |
| --- | --- | --- | --- | --- | --- | --- | --- | --- | --- | --- | --- | --- | --- | --- | --- | --- |
|  |  |  |  |  |  |  |  | **Year** |  |  |  |  |  |  |  |  |
|  | **2006** | **2007** | **2008** | **2009** | **2010** | **2011** | **2012** | **2013** | **2014** | **2015** | **2016** | **2017** | **2018** | **2019** | **2020** | ***P for trend*** |
| **Boys** |  |  |  |  |  |  |  |  |  |  |  |  |  |  |  |  |
| **Household income** |  |  |  |  |  |  |  |  |  |  |  |  |  |  |  |  |
| High | 1.00 |  |  |  |  |  |  |  |  |  |  |  |  |  |  |  |
| Middle | 0.91  (1-0.83) | 1.01  (1.12-0.92) | 0.99  (1.09-0.9) | 0.96  (1.06-0.87) | 0.92  (1.01-0.83) | 0.93  (1.02-0.85) | 0.87  (0.95-0.79) | 1.03  (1.12-0.94) | 1.02  (1.11-0.93) | 0.99  (1.08-0.91) | 1.01  (1.09-0.93) | 0.98  (1.06-0.91) | 1.02  (1.1-0.95) | 0.96  (1.03-0.9) | 1.04  (1.11-0.98) | *0.192* |
| Low | 0.99  (1.11-0.89) | 1.13  (1.26-1.01) | 1.23  (1.37-1.1) | 1.13  (1.26-1.01) | 1.04  (1.16-0.94) | 1  (1.13-0.9) | 1.07  (1.19-0.97) | 1.13  (1.26-1.01) | 1.3  (1.45-1.17) | 1.21  (1.35-1.08) | 1.24  (1.37-1.12) | 1.28  (1.42-1.16) | 1.19  (1.32-1.08) | 1.29  (1.41-1.17) | 1.25  (1.37-1.14) | *0.005* |
| **Father’s education** |  |  |  |  |  |  |  |  |  |  |  |  |  |  |  |  |
| Tertiary or above | 1.00 |  |  |  |  |  |  |  |  |  |  |  |  |  |  |  |
| Upper secondary | 0.98  (1.07-0.9) | 1.09  (1.19-0.99) | 1.12  (1.23-1.03) | 1.04  (1.14-0.95) | 1.14  (1.24-1.04) | 1.12  (1.23-1.03) | 1.16  (1.26-1.06) | 1.19  (1.3-1.1) | 1.23  (1.33-1.13) | 1.3  (1.41-1.2) | 1.21  (1.3-1.12) | 1.32  (1.42-1.23) | 1.2  (1.29-1.11) | 1.28  (1.4-1.17) | 1.31  (1.42-1.21) | *<0.001* |
| Basic or less | 0.96  (1.1-0.83) | 1.31  (1.5-1.13) | 1.14  (1.33-0.98) | 1.07  (1.26-0.91) | 1.07  (1.26-0.91) | 1.26  (1.5-1.06) | 1.27  (1.51-1.06) | 1.34  (1.6-1.12) | 1.4  (1.69-1.16) | 1.25  (1.57-1) | 1.61  (1.92-1.34) | 1.55  (1.9-1.27) | 1.26  (1.59-0.99) | 1.52  (1.95-1.18) | 1.46  (1.86-1.15) | *0.076* |
| **Mother’s education** |  |  |  |  |  |  |  |  |  |  |  |  |  |  |  |  |
| Tertiary or above | 1.00 |  |  |  |  |  |  |  |  |  |  |  |  |  |  |  |
| Upper secondary | 1.07  (1.17-0.97) | 1.07  (1.18-0.97) | 1.06  (1.16-0.97) | 1.03  (1.13-0.94) | 1.1  (1.2-1.01) | 1.03  (1.13-0.94) | 1.14  (1.24-1.05) | 1.16  (1.26-1.07) | 1.19  (1.29-1.09) | 1.28  (1.39-1.18) | 1.21  (1.31-1.13) | 1.26  (1.35-1.17) | 1.16  (1.24-1.08) | 1.23  (1.35-1.13) | 1.23  (1.33-1.14) | *0.313* |
| Basic or less | 0.98  (1.14-0.85) | 1.28  (1.48-1.11) | 1.14  (1.33-0.97) | 1.14  (1.34-0.97) | 1.12  (1.33-0.94) | 1.24  (1.48-1.04) | 1.25  (1.5-1.04) | 1.33  (1.6-1.1) | 1.08  (1.37-0.85) | 1.57  (1.96-1.26) | 1.51  (1.86-1.24) | 1.38  (1.76-1.08) | 1.19  (1.57-0.91) | 1.79  (2.33-1.37) | 1.31  (1.75-0.98) | *0.910* |
| **Urbanicity** |  |  |  |  |  |  |  |  |  |  |  |  |  |  |  |  |
| Metropolitan cities | 1.00 |  |  |  |  |  |  |  |  |  |  |  |  |  |  |  |
| Other cities | 0.95  (1.03-0.87) | 0.92  (1-0.84) | 1.01  (1.1-0.92) | 1  (1.09-0.91) | 1.02  (1.12-0.93) | 0.99  (1.09-0.91) | 0.99  (1.08-0.91) | 1.13  (1.23-1.04) | 1.01  (1.1-0.94) | 0.94  (1.02-0.87) | 0.98  (1.05-0.91) | 1.04  (1.12-0.97) | 0.99  (1.06-0.92) | 0.99  (1.06-0.93) | 0.94  (1.01-0.89) | *0.275* |
| Rural areas | 0.82  (0.94-0.72) | 0.98  (1.11-0.86) | 0.93  (1.07-0.82) | 0.89  (1.03-0.78) | 1.11  (1.25-0.98) | 0.93  (1.06-0.81) | 1.01  (1.15-0.88) | 1.04  (1.18-0.92) | 1.12  (1.28-0.97) | 1.18  (1.36-1.03) | 1.22  (1.39-1.07) | 1.17  (1.33-1.03) | 1.21  (1.37-1.07) | 1.08  (1.22-0.96) | 1.04  (1.17-0.93) | *<0.001* |
| **Girls** |  |  |  |  |  |  |  |  |  |  |  |  |  |  |  |  |
| **Household income** |  |  |  |  |  |  |  |  |  |  |  |  |  |  |  |  |
| High | 1.00 |  |  |  |  |  |  |  |  |  |  |  |  |  |  |  |
| Middle | 1.09  (1.25-0.95) | 1.09  (1.26-0.94) | 1.08  (1.26-0.93) | 0.98  (1.14-0.85) | 1.09  (1.28-0.94) | 1.11  (1.27-0.97) | 1.05  (1.19-0.92) | 1.26  (1.43-1.12) | 1.25  (1.42-1.11) | 1.36  (1.53-1.21) | 1.21  (1.35-1.08) | 1.16  (1.29-1.05) | 1.12  (1.24-1.01) | 1.14  (1.25-1.03) | 1.28  (1.42-1.15) | *0.018* |
| Low | 1.66  (1.93-1.42) | 1.53  (1.79-1.31) | 1.72  (2.01-1.47) | 1.45  (1.7-1.24) | 1.67  (1.97-1.42) | 1.64  (1.88-1.42) | 1.68  (1.92-1.46) | 1.84  (2.11-1.61) | 1.9  (2.18-1.65) | 1.99  (2.28-1.74) | 1.88  (2.14-1.65) | 1.8  (2.03-1.59) | 1.78  (2.02-1.57) | 1.77  (2.01-1.57) | 1.98  (2.25-1.75) | *0.002* |
| **Father’s education** |  |  |  |  |  |  |  |  |  |  |  |  |  |  |  |  |
| Tertiary or above | 1.00 |  |  |  |  |  |  |  |  |  |  |  |  |  |  |  |
| Upper secondary | 1.35  (1.52-1.19) | 1.24  (1.41-1.1) | 1.36  (1.54-1.2) | 1.31  (1.49-1.15) | 1.26  (1.43-1.1) | 1.47  (1.65-1.32) | 1.34  (1.49-1.2) | 1.6  (1.78-1.44) | 1.57  (1.74-1.41) | 1.58  (1.75-1.43) | 1.57  (1.73-1.43) | 1.6  (1.76-1.45) | 1.63  (1.79-1.48) | 1.63  (1.81-1.46) | 1.76  (1.95-1.59) | *<0.001* |
| Basic or less | 1.71  (2.03-1.43) | 1.48  (1.8-1.21) | 1.46  (1.8-1.19) | 1.83  (2.22-1.5) | 1.92  (2.37-1.56) | 1.87  (2.28-1.54) | 2.02  (2.45-1.67) | 2.22  (2.71-1.82) | 2.15  (2.7-1.71) | 2.13  (2.67-1.7) | 1.86  (2.38-1.45) | 2.39  (2.97-1.92) | 2.05  (2.65-1.59) | 2.05  (2.78-1.52) | 2.11  (2.79-1.6) | *0.015* |
| **Mother’s education** |  |  |  |  |  |  |  |  |  |  |  |  |  |  |  |  |
| Tertiary or above | 1.00 |  |  |  |  |  |  |  |  |  |  |  |  |  |  |  |
| Upper secondary | 1.29  (1.49-1.12) | 1.17  (1.35-1.02) | 1.37  (1.57-1.19) | 1.29  (1.48-1.13) | 1.27  (1.46-1.11) | 1.34  (1.5-1.19) | 1.35  (1.51-1.21) | 1.52  (1.69-1.36) | 1.55  (1.73-1.39) | 1.65  (1.82-1.49) | 1.45  (1.59-1.31) | 1.4  (1.53-1.27) | 1.56  (1.71-1.42) | 1.51  (1.68-1.36) | 1.62  (1.79-1.46) | *0.002* |
| Basic or less | 1.69  (2.02-1.41) | 1.59  (1.94-1.31) | 1.55  (1.91-1.26) | 1.39  (1.75-1.11) | 1.8  (2.24-1.44) | 1.79  (2.19-1.46) | 1.95  (2.39-1.59) | 1.72  (2.16-1.37) | 2.37  (2.99-1.88) | 2.56  (3.21-2.05) | 1.93  (2.5-1.49) | 2.35  (2.94-1.88) | 1.82  (2.43-1.37) | 1.81  (2.53-1.29) | 1.79  (2.55-1.26) | *0.239* |
| **Urbanicity** |  |  |  |  |  |  |  |  |  |  |  |  |  |  |  |  |
| Metropolitan cities | 1.00 |  |  |  |  |  |  |  |  |  |  |  |  |  |  |  |
| Other cities | 0.99  (1.11-0.87) | 1.04  (1.17-0.92) | 1.27  (1.43-1.12) | 1.18  (1.34-1.04) | 1.29  (1.48-1.13) | 1.09  (1.22-0.98) | 1.12  (1.24-1) | 1.14  (1.26-1.02) | 1.09  (1.21-0.98) | 1.25  (1.38-1.13) | 1.13  (1.24-1.02) | 1.04  (1.15-0.95) | 1.15  (1.26-1.04) | 1.02  (1.12-0.93) | 1.07  (1.18-0.98) | *0.445* |
| Rural areas | 1.21  (1.41-1.03) | 1.08  (1.29-0.91) | 1.23  (1.47-1.04) | 1.28  (1.52-1.08) | 1.53  (1.82-1.29) | 1.17  (1.37-0.99) | 1.37  (1.61-1.17) | 1.26  (1.48-1.08) | 1.37  (1.65-1.14) | 1.56  (1.85-1.32) | 1.61  (1.89-1.38) | 1.4  (1.64-1.19) | 1.45  (1.7-1.23) | 1.48  (1.72-1.28) | 1.34  (1.57-1.14) | *0.006* |
|  |  |  |  |  |  |  |  |  |  |  |  |  |  |  |  |  |

| **Supplementary Material 4 *(continued)*. Prevalence ratio and 95% confidence intervals by socioeconomic status from 2006 to 2020 (according to school stage)** | | | | | | | | | | | | | | | | |
| --- | --- | --- | --- | --- | --- | --- | --- | --- | --- | --- | --- | --- | --- | --- | --- | --- |
|  |  |  |  |  |  |  |  | **Year** |  |  |  |  |  |  |  |  |
|  | **2006** | **2007** | **2008** | **2009** | **2010** | **2011** | **2012** | **2013** | **2014** | **2015** | **2016** | **2017** | **2018** | **2019** | **2020** | ***P for trend*** |
| **High school** |  |  |  |  |  |  |  |  |  |  |  |  |  |  |  |  |
| **Household income** |  |  |  |  |  |  |  |  |  |  |  |  |  |  |  |  |
| High | 1.00 |  |  |  |  |  |  |  |  |  |  |  |  |  |  |  |
| Middle | 0.86  (0.95-0.77) | 0.91  (1.02-0.82) | 0.94  (1.05-0.84) | 0.88  (0.98-0.78) | 0.94  (1.06-0.84) | 0.87  (0.97-0.78) | 0.84  (0.93-0.76) | 0.95  (1.05-0.87) | 1  (1.1-0.91) | 0.94  (1.03-0.86) | 0.99  (1.08-0.91) | 0.91  (0.99-0.84) | 0.92  (1-0.86) | 0.97  (1.05-0.9) | 1.04  (1.12-0.97) | *0.051* |
| Low | 1.07  (1.2-0.95) | 1.09  (1.22-0.97) | 1.3  (1.46-1.15) | 1.14  (1.29-1.01) | 1.17  (1.33-1.04) | 1.14  (1.28-1.02) | 1.16  (1.29-1.04) | 1.16  (1.29-1.04) | 1.31  (1.46-1.18) | 1.23  (1.36-1.11) | 1.29  (1.42-1.17) | 1.26  (1.39-1.15) | 1.16  (1.28-1.05) | 1.32  (1.44-1.2) | 1.36  (1.5-1.24) | *0.040* |
| **Father’s education** |  |  |  |  |  |  |  |  |  |  |  |  |  |  |  |  |
| Tertiary or above | 1.00 |  |  |  |  |  |  |  |  |  |  |  |  |  |  |  |
| Upper secondary | 1.02  (1.12-0.93) | 1.09  (1.2-0.99) | 1.15  (1.27-1.04) | 1.03  (1.14-0.93) | 1.1  (1.22-1) | 1.12  (1.23-1.02) | 1.08  (1.18-0.99) | 1.22  (1.32-1.12) | 1.22  (1.33-1.12) | 1.27  (1.38-1.17) | 1.21  (1.3-1.12) | 1.27  (1.37-1.18) | 1.25  (1.34-1.16) | 1.26  (1.38-1.16) | 1.39  (1.51-1.28) | *<0.001* |
| Basic or less | 1.1  (1.26-0.96) | 1.19  (1.38-1.03) | 1.15  (1.34-0.98) | 1.19  (1.4-1.02) | 1.32  (1.55-1.12) | 1.29  (1.52-1.09) | 1.42  (1.67-1.21) | 1.45  (1.72-1.23) | 1.51  (1.81-1.27) | 1.25  (1.53-1.02) | 1.42  (1.71-1.19) | 1.63  (1.95-1.36) | 1.41  (1.74-1.15) | 1.45  (1.84-1.14) | 1.64  (2.07-1.3) | *0.114* |
| **Mother’s education** |  |  |  |  |  |  |  |  |  |  |  |  |  |  |  |  |
| Tertiary or above | 1.00 |  |  |  |  |  |  |  |  |  |  |  |  |  |  |  |
| Upper secondary | 1.05  (1.18-0.95) | 1.06  (1.18-0.95) | 1.06  (1.18-0.95) | 0.99  (1.1-0.9) | 1.05  (1.16-0.95) | 0.99  (1.09-0.89) | 1.15  (1.26-1.05) | 1.1  (1.2-1.01) | 1.17  (1.27-1.07) | 1.25  (1.36-1.16) | 1.17  (1.26-1.09) | 1.19  (1.28-1.11) | 1.14  (1.23-1.06) | 1.2  (1.31-1.1) | 1.3  (1.41-1.19) | *0.094* |
| Basic or less | 1.04  (1.19-0.9) | 1.24  (1.44-1.08) | 1.16  (1.36-0.99) | 1.06  (1.26-0.9) | 1.22  (1.45-1.03) | 1.28  (1.51-1.09) | 1.28  (1.53-1.07) | 1.29  (1.54-1.09) | 1.44  (1.75-1.18) | 1.77  (2.13-1.48) | 1.47  (1.78-1.21) | 1.44  (1.77-1.17) | 1.3  (1.64-1.03) | 1.61  (2.08-1.25) | 1.38  (1.87-1.02) | *0.595* |
| **Urbanicity** |  |  |  |  |  |  |  |  |  |  |  |  |  |  |  |  |
| Metropolitan cities | 1.00 |  |  |  |  |  |  |  |  |  |  |  |  |  |  |  |
| Other cities | 0.91  (1-0.83) | 0.93  (1.02-0.85) | 1.06  (1.16-0.96) | 0.98  (1.08-0.88) | 1.05  (1.16-0.95) | 0.92  (1.01-0.84) | 0.97  (1.06-0.89) | 1.06  (1.16-0.98) | 0.97  (1.05-0.89) | 1.04  (1.13-0.96) | 1.02  (1.1-0.95) | 1.01  (1.09-0.94) | 1  (1.07-0.93) | 0.96  (1.03-0.9) | 1  (1.07-0.93) | *0.090* |
| Rural areas | 0.88  (1.01-0.77) | 1  (1.14-0.88) | 0.97  (1.12-0.84) | 0.94  (1.09-0.81) | 1.2  (1.37-1.06) | 0.9  (1.04-0.78) | 0.98  (1.13-0.84) | 1.01  (1.15-0.88) | 1.1  (1.28-0.95) | 1.09  (1.26-0.95) | 1.28  (1.46-1.12) | 1.08  (1.22-0.95) | 1.13  (1.29-0.99) | 1.19  (1.33-1.06) | 1.07  (1.21-0.94) | *0.003* |
| **Middle school** |  |  |  |  |  |  |  |  |  |  |  |  |  |  |  |  |
| **Household income** |  |  |  |  |  |  |  |  |  |  |  |  |  |  |  |  |
| High | 1.00 |  |  |  |  |  |  |  |  |  |  |  |  |  |  |  |
| Middle | 0.93  (1.04-0.83) | 1.03  (1.16-0.92) | 0.97  (1.09-0.86) | 0.94  (1.05-0.84) | 0.86  (0.96-0.77) | 1  (1.11-0.89) | 0.93  (1.04-0.84) | 1.13  (1.25-1.01) | 1.07  (1.18-0.96) | 1.2  (1.34-1.08) | 1.02  (1.12-0.92) | 1.07  (1.18-0.97) | 1.08  (1.19-0.98) | 0.93  (1.02-0.86) | 1.07  (1.16-0.98) | *0.036* |
| Low | 1.11  (1.28-0.96) | 1.24  (1.43-1.08) | 1.25  (1.43-1.09) | 1.2  (1.37-1.05) | 1.11  (1.27-0.97) | 1.11  (1.28-0.96) | 1.3  (1.47-1.14) | 1.46  (1.67-1.27) | 1.57  (1.81-1.37) | 1.55  (1.8-1.34) | 1.5  (1.72-1.31) | 1.47  (1.7-1.28) | 1.55  (1.78-1.35) | 1.37  (1.55-1.2) | 1.45  (1.63-1.29) | *<0.001* |
| **Father’s education** |  |  |  |  |  |  |  |  |  |  |  |  |  |  |  |  |
| Tertiary or above | 1.00 |  |  |  |  |  |  |  |  |  |  |  |  |  |  |  |
| Upper secondary | 1.13  (1.25-1.01) | 1.15  (1.29-1.03) | 1.2  (1.34-1.08) | 1.2  (1.33-1.08) | 1.23  (1.36-1.1) | 1.35  (1.5-1.22) | 1.39  (1.54-1.26) | 1.47  (1.62-1.32) | 1.46  (1.62-1.32) | 1.51  (1.68-1.36) | 1.46  (1.61-1.33) | 1.57  (1.73-1.43) | 1.41  (1.55-1.29) | 1.53  (1.71-1.37) | 1.48  (1.62-1.34) | *<0.001* |
| Basic or less | 1.19  (1.43-0.99) | 1.58  (1.91-1.31) | 1.3  (1.59-1.06) | 1.44  (1.75-1.18) | 1.29  (1.59-1.04) | 1.69  (2.1-1.37) | 1.67  (2.07-1.35) | 1.9  (2.37-1.53) | 1.85  (2.36-1.44) | 2.16  (2.79-1.66) | 2.16  (2.75-1.69) | 2.1  (2.72-1.63) | 1.57  (2.15-1.14) | 2.06  (2.88-1.48) | 1.61  (2.17-1.19) | *0.002* |
| **Mother’s education** |  |  |  |  |  |  |  |  |  |  |  |  |  |  |  |  |
| Tertiary or above | 1.00 |  |  |  |  |  |  |  |  |  |  |  |  |  |  |  |
| Upper secondary | 1.12  (1.26-1) | 1.05  (1.18-0.93) | 1.18  (1.32-1.05) | 1.17  (1.31-1.05) | 1.2  (1.34-1.08) | 1.24  (1.38-1.12) | 1.24  (1.37-1.12) | 1.46  (1.62-1.32) | 1.41  (1.56-1.27) | 1.5  (1.67-1.36) | 1.36  (1.5-1.24) | 1.35  (1.48-1.22) | 1.41  (1.55-1.29) | 1.39  (1.56-1.25) | 1.33  (1.46-1.21) | *0.003* |
| Basic or less | 1.3  (1.56-1.08) | 1.39  (1.7-1.14) | 1.18  (1.47-0.94) | 1.35  (1.67-1.09) | 1.35  (1.69-1.08) | 1.45  (1.83-1.15) | 1.84  (2.27-1.5) | 1.55  (2.01-1.2) | 1.44  (1.95-1.07) | 1.82  (2.48-1.33) | 1.87  (2.47-1.41) | 2.15  (2.83-1.63) | 1.35  (1.96-0.93) | 1.77  (2.55-1.22) | 1.52  (2.13-1.08) | *0.286* |
| **Urbanicity** |  |  |  |  |  |  |  |  |  |  |  |  |  |  |  |  |
| Metropolitan cities | 1.00 |  |  |  |  |  |  |  |  |  |  |  |  |  |  |  |
| Other cities | 0.99  (1.1-0.88) | 1.01  (1.13-0.9) | 1.12  (1.25-1.01) | 1.14  (1.27-1.03) | 1.12  (1.24-1) | 1.18  (1.31-1.06) | 1.11  (1.23-1) | 1.21  (1.34-1.09) | 1.16  (1.28-1.04) | 1.09  (1.21-0.98) | 1.08  (1.19-0.98) | 1.14  (1.26-1.04) | 1.12  (1.23-1.02) | 1.06  (1.15-0.97) | 0.95  (1.03-0.88) | *0.849* |
| Rural areas | 1.04  (1.21-0.89) | 1.04  (1.22-0.89) | 1.11  (1.29-0.95) | 1.1  (1.29-0.94) | 1.25  (1.46-1.08) | 1.2  (1.39-1.03) | 1.37  (1.58-1.18) | 1.35  (1.56-1.16) | 1.45  (1.72-1.22) | 1.74  (2.04-1.48) | 1.51  (1.77-1.29) | 1.63  (1.9-1.39) | 1.6  (1.85-1.38) | 1.25  (1.45-1.07) | 1.2  (1.39-1.04) | *<0.001* |
|  | | | | | | | | | | | | | | | | |
