# Supplementary material for: Trends in socioeconomic inequalities in obesity among Korean adolescents: the Korea Youth Risk Behavior Web-based Survey (KYRBS) 2006 to 2020
Source: Epidemiol Health. 2023 Mar 7;45:e2023033. doi: 10.4178/epih.e2023033 (PMC10586920; doi:10.4178/epih.e2023033)
Supplement: Supplementary Material 5. — Relative Index of Inequality (RII) based on the prevalence ratio from 2006 to 2020 [file epih-45-e2023033-Supplementary-5.docx]

| **Supplementary Material 5. Relative Index of Inequality (RII) based on the prevalence ratio from 2006 to 2020** | | | | | | | | | | | | | | | |  |
| --- | --- | --- | --- | --- | --- | --- | --- | --- | --- | --- | --- | --- | --- | --- | --- | --- |
|  |  |  |  |  |  |  |  | **Year** |  |  |  |  |  |  |  |  |
|  | **2006** | **2007** | **2008** | **2009** | **2010** | **2011** | **2012** | **2013** | **2014** | **2015** | **2016** | **2017** | **2018** | **2019** | **2020** | ***P for trend*** |
| **Household income** |  |  |  |  |  |  |  |  |  |  |  |  |  |  |  |  |
| Total | 1.21 (1.19-1.23) | 1.27 (1.25-1.3) | 1.47 (1.44-1.49) | 1.23 (1.2-1.25) | 1.22 (1.2-1.24) | 1.27 (1.25-1.29) | 1.33 (1.31-1.36) | 1.45 (1.43-1.48) | 1.62 (1.6-1.65) | 1.55 (1.52-1.57) | 1.43 (1.41-1.46) | 1.40 (1.38-1.43) | 1.30 (1.28-1.32) | 1.35 (1.33-1.36) | 1.50 (1.48-1.52) | *<0.001* |
| Boys | 0.96 (0.94-0.98) | 1.13 (1.11-1.16) | 1.36 (1.34-1.39) | 1.16 (1.13-1.18) | 1.04 (1.02-1.07) | 1.04 (1.02-1.06) | 1.06 (1.04-1.08) | 1.22 (1.2-1.25) | 1.38 (1.35-1.41) | 1.17 (1.15-1.2) | 1.23 (1.21-1.25) | 1.27 (1.25-1.29) | 1.12 (1.1-1.14) | 1.19 (1.17-1.21) | 1.25 (1.23-1.27) | *<0.001* |
| Girls | 2.25 (2.19-2.32) | 1.80 (1.74-1.85) | 1.99 (1.93-2.05) | 1.64 (1.59-1.69) | 2.22 (2.15-2.3) | 2.11 (2.05-2.17) | 2.24 (2.18-2.31) | 2.29 (2.22-2.35) | 2.53 (2.46-2.6) | 2.58 (2.51-2.65) | 2.10 (2.04-2.15) | 1.95 (1.9-2) | 1.95 (1.9-2) | 1.94 (1.89-1.99) | 2.49 (2.42-2.55) | *<0.001* |
| High school | 1.15 (1.12-1.17) | 1.16 (1.14-1.19) | 1.52 (1.49-1.56) | 1.24 (1.21-1.27) | 1.36 (1.33-1.39) | 1.23 (1.21-1.26) | 1.32 (1.29-1.35) | 1.18 (1.16-1.2) | 1.41 (1.38-1.44) | 1.26 (1.23-1.28) | 1.27 (1.25-1.3) | 1.19 (1.17-1.21) | 1.06 (1.04-1.08) | 1.27 (1.25-1.29) | 1.41 (1.39-1.44) | *<0.001* |
| Middle school | 1.10 (1.07-1.13) | 1.21 (1.18-1.24) | 1.26 (1.23-1.3) | 1.15 (1.12-1.18) | 1.00 (0.97-1.02) | 1.23 (1.19-1.26) | 1.20 (1.17-1.23) | 1.65 (1.61-1.7) | 1.73 (1.69-1.78) | 1.65 (1.6-1.7) | 1.38 (1.34-1.42) | 1.50 (1.46-1.54) | 1.50 (1.46-1.54) | 1.19 (1.16-1.22) | 1.43 (1.4-1.46) | *<0.001* |
|  |  |  |  |  |  |  |  |  |  |  |  |  |  |  |  |  |
| **Father’s education** |  |  |  |  |  |  |  |  |  |  |  |  |  |  |  |  |
| Total | 1.37 (1.35-1.4) | 1.50 (1.48-1.53) | 1.56 (1.53-1.59) | 1.30 (1.28-1.33) | 1.51 (1.48-1.54) | 1.64 (1.61-1.67) | 1.63 (1.6-1.65) | 1.81 (1.78-1.85) | 1.75 (1.72-1.79) | 1.95 (1.92-1.99) | 1.85 (1.82-1.89) | 2.05 (2.02-2.08) | 1.77 (1.74-1.79) | 2.03 (1.99-2.07) | 2.18 (2.14-2.22) | *<0.001* |
| Boys | 1.00 (0.98-1.02) | 1.34 (1.31-1.37) | 1.37 (1.34-1.4) | 1.01 (0.98-1.03) | 1.22 (1.2-1.25) | 1.33 (1.3-1.36) | 1.39 (1.36-1.42) | 1.46 (1.43-1.5) | 1.44 (1.41-1.48) | 1.65 (1.62-1.69) | 1.53 (1.5-1.56) | 1.77 (1.74-1.81) | 1.37 (1.35-1.4) | 1.71 (1.66-1.75) | 1.79 (1.75-1.83) | *<0.001* |
| Girls | 2.54 (2.47-2.62) | 1.88 (1.82-1.94) | 2.03 (1.96-2.09) | 2.26 (2.19-2.33) | 2.46 (2.38-2.54) | 2.45 (2.38-2.53) | 2.20 (2.14-2.27) | 2.69 (2.61-2.77) | 2.56 (2.49-2.64) | 2.62 (2.54-2.69) | 2.67 (2.6-2.74) | 2.72 (2.65-2.79) | 2.76 (2.69-2.83) | 2.57 (2.49-2.65) | 3.17 (3.08-3.26) | *<0.001* |
| High school | 1.32 (1.29-1.35) | 1.32 (1.29-1.35) | 1.48 (1.44-1.52) | 1.15 (1.12-1.18) | 1.53 (1.5-1.57) | 1.28 (1.25-1.32) | 1.37 (1.34-1.4) | 1.51 (1.48-1.55) | 1.49 (1.45-1.52) | 1.57 (1.54-1.6) | 1.50 (1.47-1.53) | 1.62 (1.59-1.65) | 1.49 (1.46-1.52) | 1.58 (1.54-1.62) | 1.94 (1.89-1.99) | *<0.001* |
| Middle school | 1.28 (1.25-1.32) | 1.59 (1.54-1.64) | 1.54 (1.5-1.59) | 1.49 (1.45-1.54) | 1.43 (1.39-1.47) | 2.16 (2.1-2.22) | 1.95 (1.9-2) | 2.19 (2.13-2.25) | 2.07 (2.01-2.13) | 2.38 (2.3-2.45) | 2.37 (2.3-2.43) | 2.69 (2.62-2.77) | 2.01 (1.96-2.07) | 2.44 (2.36-2.53) | 2.28 (2.21-2.35) | *<0.001* |
|  |  |  |  |  |  |  |  |  |  |  |  |  |  |  |  |  |
| **Mother’s education** |  |  |  |  |  |  |  |  |  |  |  |  |  |  |  |  |
| Total | 1.49 (1.46-1.51) | 1.47 (1.44-1.5) | 1.34 (1.32-1.37) | 1.19 (1.17-1.21) | 1.35 (1.33-1.38) | 1.38 (1.35-1.4) | 1.55 (1.53-1.58) | 1.60 (1.57-1.63) | 1.64 (1.61-1.67) | 2.02 (1.99-2.06) | 1.73 (1.7-1.75) | 1.77 (1.74-1.8) | 1.59 (1.56-1.62) | 1.86 (1.83-1.9) | 1.75 (1.72-1.78) | *<0.001* |
| Boys | 1.16 (1.13-1.18) | 1.35 (1.32-1.38) | 1.18 (1.15-1.21) | 1.06 (1.03-1.08) | 1.16 (1.13-1.18) | 1.13 (1.1-1.15) | 1.38 (1.35-1.41) | 1.39 (1.36-1.42) | 1.32 (1.29-1.35) | 1.68 (1.64-1.72) | 1.52 (1.49-1.55) | 1.63 (1.6-1.67) | 1.25 (1.23-1.28) | 1.63 (1.59-1.67) | 1.45 (1.41-1.48) | *<0.001* |
| Girls | 2.50 (2.42-2.58) | 1.86 (1.79-1.92) | 1.86 (1.8-1.92) | 1.73 (1.68-1.79) | 2.17 (2.1-2.25) | 2.12 (2.06-2.19) | 2.02 (1.96-2.08) | 2.24 (2.17-2.3) | 2.65 (2.57-2.73) | 2.90 (2.82-2.98) | 2.33 (2.26-2.39) | 2.23 (2.18-2.29) | 2.53 (2.46-2.59) | 2.32 (2.25-2.39) | 2.62 (2.55-2.7) | *<0.001* |
| High school | 1.36 (1.33-1.4) | 1.30 (1.26-1.33) | 1.27 (1.24-1.3) | 1.07 (1.04-1.09) | 1.30 (1.26-1.33) | 1.10 (1.08-1.13) | 1.38 (1.35-1.41) | 1.29 (1.26-1.32) | 1.38 (1.35-1.41) | 1.67 (1.64-1.71) | 1.43 (1.4-1.46) | 1.46 (1.44-1.49) | 1.26 (1.23-1.28) | 1.51 (1.47-1.55) | 1.57 (1.54-1.61) | *<0.001* |
| Middle school | 1.41 (1.37-1.45) | 1.46 (1.42-1.5) | 1.32 (1.28-1.36) | 1.31 (1.27-1.34) | 1.36 (1.32-1.4) | 1.72 (1.67-1.77) | 1.67 (1.63-1.72) | 1.93 (1.87-1.98) | 1.90 (1.84-1.95) | 2.21 (2.14-2.28) | 2.01 (1.95-2.07) | 2.00 (1.94-2.05) | 1.96 (1.91-2.02) | 2.05 (1.98-2.12) | 1.78 (1.73-1.83) | *<0.001* |
|  |  |  |  |  |  |  |  |  |  |  |  |  |  |  |  |  |
| **Urbanicity** |  |  |  |  |  |  |  |  |  |  |  |  |  |  |  |  |
| Total | 0.91 (0.89-0.93) | 0.81 (0.79-0.83) | 0.96 (0.94-0.98) | 1.02 (1-1.04) | 1.21 (1.18-1.23) | 0.99 (0.97-1.01) | 1.15 (1.13-1.17) | 1.17 (1.15-1.2) | 1.13 (1.11-1.15) | 1.13 (1.11-1.15) | 1.13 (1.11-1.15) | 1.11 (1.1-1.13) | 1.13 (1.12-1.15) | 1.07 (1.05-1.09) | 0.97 (0.96-0.99) | *<0.001* |
| Boys | 0.79 (0.77-0.81) | 0.74 (0.73-0.76) | 0.80 (0.78-0.82) | 0.93 (0.91-0.95) | 1.07 (1.04-1.09) | 0.90 (0.88-0.92) | 1.06 (1.03-1.08) | 1.13 (1.1-1.15) | 1.04 (1.02-1.07) | 0.91 (0.89-0.93) | 1.00 (0.98-1.02) | 1.07 (1.05-1.09) | 1.00 (0.98-1.02) | 1.01 (0.99-1.03) | 0.88 (0.87-0.9) | *0.003* |
| Girls | 1.18 (1.15-1.22) | 0.96 (0.93-0.99) | 1.34 (1.3-1.39) | 1.21 (1.17-1.25) | 1.58 (1.52-1.64) | 1.18 (1.14-1.21) | 1.31 (1.27-1.35) | 1.26 (1.22-1.29) | 1.28 (1.24-1.32) | 1.53 (1.49-1.58) | 1.41 (1.37-1.45) | 1.20 (1.17-1.23) | 1.40 (1.36-1.44) | 1.18 (1.15-1.21) | 1.17 (1.14-1.2) | *<0.001* |
| High school | 0.85 (0.83-0.87) | 0.72 (0.7-0.73) | 0.87 (0.85-0.9) | 0.81 (0.79-0.83) | 1.12 (1.09-1.15) | 0.77 (0.75-0.79) | 1.00 (0.97-1.02) | 1.04 (1.02-1.06) | 0.98 (0.96-1) | 0.99 (0.97-1.01) | 1.06 (1.04-1.08) | 0.96 (0.94-0.98) | 0.99 (0.97-1.01) | 1.01 (0.99-1.02) | 0.94 (0.92-0.96) | *0.003* |
| Middle school | 1.02 (0.99-1.05) | 0.99 (0.96-1.02) | 1.09 (1.06-1.13) | 1.35 (1.31-1.39) | 1.33 (1.29-1.37) | 1.40 (1.36-1.45) | 1.42 (1.38-1.47) | 1.46 (1.42-1.5) | 1.40 (1.37-1.44) | 1.47 (1.43-1.51) | 1.29 (1.25-1.33) | 1.47 (1.43-1.51) | 1.45 (1.41-1.49) | 1.17 (1.14-1.2) | 1.03 (1.01-1.06) | *<0.001* |
