# Supplementary material for: Trends in socioeconomic inequalities in obesity among Korean adolescents: the Korea Youth Risk Behavior Web-based Survey (KYRBS) 2006 to 2020
Source: Epidemiol Health. 2023 Mar 7;45:e2023033. doi: 10.4178/epih.e2023033 (PMC10586920; doi:10.4178/epih.e2023033)
Supplement: Supplementary Material 6. — Odds ratio and 95% confidence intervals by socioeconomic status from 2006 to 2020 (among the total study group) [file epih-45-e2023033-Supplementary-6.docx]

| **Supplementary Material 6. Odds ratio and 95% confidence intervals by socioeconomic status from 2006 to 2020 (among the total study group)** | | | | | | | | | | | | | | | |  |
| --- | --- | --- | --- | --- | --- | --- | --- | --- | --- | --- | --- | --- | --- | --- | --- | --- |
|  |  |  |  |  |  |  |  | **Year** |  |  |  |  |  |  |  |  |
|  | **2006** | **2007** | **2008** | **2009** | **2010** | **2011** | **2012** | **2013** | **2014** | **2015** | **2016** | **2017** | **2018** | **2019** | **2020** | ***P for trend*** |
| **Total** |  |  |  |  |  |  |  |  |  |  |  |  |  |  |  |  |
| **Household income** |  |  |  |  |  |  |  |  |  |  |  |  |  |  |  |  |
| High | 1.00 |  |  |  |  |  |  |  |  |  |  |  |  |  |  |  |
| Middle | 0.91  (0.82-1.02) | 1.01  (0.91-1.12) | 0.93  (0.84-1.02) | 0.91  (0.82-1.00) | 0.91  (0.82-1.00) | 0.96  (0.88-1.04) | 0.88  (0.82-0.95) | 1.05  (0.97-1.14) | 1.07  (0.99-1.15) | 1.09  (1.01-1.17) | 1.02  (0.95-1.10) | 1.00  (0.93-1.07) | 1.02  (0.96-1.09) | 0.98  (0.92-1.05) | 1.08  (1.01-1.15) | *0.004* |
| Low | 1.21  (1.07-1.36) | 1.21  (1.08-1.36) | 1.36  (1.23-1.51) | 1.19  (1.07-1.32) | 1.19  (1.07-1.32) | 1.23  (1.12-1.36) | 1.30  (1.19-1.42) | 1.38  (1.26-1.52) | 1.55  (1.41-1.69) | 1.49  (1.35-1.64) | 1.47  (1.34-1.61) | 1.50  (1.37-1.65) | 1.39  (1.26-1.53) | 1.50  (1.38-1.63) | 1.57  (1.44-1.71) | *<0.001* |
| **Father’s education** |  |  |  |  |  |  |  |  |  |  |  |  |  |  |  |  |
| Tertiary or above | 1.00 |  |  |  |  |  |  |  |  |  |  |  |  |  |  |  |
| Upper secondary | 1.17  (1.05-1.29) | 1.20  (1.09-1.31) | 1.28  (1.18-1.39) | 1.10  (0.99-1.21) | 1.22  (1.12-1.33) | 1.25  (1.15-1.36) | 1.24  (1.15-1.34) | 1.34  (1.24-1.45) | 1.31  (1.21-1.41) | 1.44  (1.34-1.55) | 1.37  (1.29-1.46) | 1.44  (1.34-1.55) | 1.37  (1.28-1.47) | 1.46  (1.34-1.59) | 1.54  (1.43-1.66) | *<0.001* |
| Basic or less | 1.28  (1.11-1.46) | 1.42  (1.22-1.67) | 1.30  (1.11-1.53) | 1.34  (1.15-1.56) | 1.38  (1.17-1.62) | 1.55  (1.34-1.78) | 1.60  (1.37-1.87) | 1.65  (1.41-1.94) | 1.72  (1.46-2.02) | 1.50  (1.25-1.81) | 1.75  (1.48-2.08) | 2.02  (1.71-2.39) | 1.52  (1.25-1.86) | 1.95  (1.54-2.46) | 1.85  (1.46-2.35) | *0.010* |
| **Mother’s education** |  |  |  |  |  |  |  |  |  |  |  |  |  |  |  |  |
| Tertiary or above | 1.00 |  |  |  |  |  |  |  |  |  |  |  |  |  |  |  |
| Upper secondary | 1.23  (1.1-1.38) | 1.15  (1.04-1.26) | 1.12  (1.03-1.23) | 1.03  (0.93-1.15) | 1.11  (1.01-1.22) | 1.12  (1.04-1.21) | 1.21  (1.12-1.30) | 1.27  (1.18-1.36) | 1.29  (1.20-1.38) | 1.43  (1.33-1.53) | 1.31  (1.23-1.40) | 1.33  (1.25-1.43) | 1.30  (1.22-1.38) | 1.39  (1.28-1.51) | 1.37  (1.27-1.47) | *0.005* |
| Basic or less | 1.37  (1.19-1.59) | 1.46  (1.26-1.69) | 1.34  (1.14-1.58) | 1.30  (1.08-1.55) | 1.43  (1.20-1.69) | 1.49  (1.28-1.73) | 1.61  (1.38-1.89) | 1.50  (1.27-1.77) | 1.52  (1.27-1.82) | 1.92  (1.60-2.31) | 1.83  (1.52-2.21) | 1.97  (1.65-2.35) | 1.36  (1.08-1.71) | 1.93  (1.50-2.48) | 1.54  (1.16-2.04) | *0.730* |
| **Urbanicity** |  |  |  |  |  |  |  |  |  |  |  |  |  |  |  |  |
| Metropolitan cities | 1.00 |  |  |  |  |  |  |  |  |  |  |  |  |  |  |  |
| Other cities | 0.95  (0.86-1.05) | 0.87  (0.78-0.97) | 0.98  (0.88-1.08) | 1.00  (0.89-1.12) | 1.04  (0.94-1.17) | 0.99  (0.90-1.08) | 1.04  (0.96-1.14) | 1.08  (1.00-1.17) | 1.01  (0.93-1.10) | 1.00  (0.92-1.09) | 1.00  (0.92-1.09) | 1.00  (0.93-1.08) | 1.01  (0.93-1.10) | 0.97  (0.90-1.05) | 0.95  (0.87-1.02) | *0.274* |
| Rural areas | 0.96  (0.84-1.10) | 0.98  (0.87-1.11) | 0.99  (0.87-1.12) | 1.03  (0.90-1.19) | 1.29  (1.13-1.47) | 1.02  (0.91-1.16) | 1.19  (1.05-1.35) | 1.13  (0.98-1.31) | 1.25  (1.08-1.45) | 1.30  (1.12-1.50) | 1.36  (1.17-1.58) | 1.30  (1.12-1.50) | 1.31  (1.15-1.51) | 1.31  (1.17-1.48) | 1.14  (0.97-1.32) | *<0.001* |
|  |  |  |  |  |  |  |  |  |  |  |  |  |  |  |  |  |

| **Supplementary Material 6 *(continued)*. Odds ratio and 95% confidence intervals by socioeconomic status from 2006 to 2020 (according to sex)** | | | | | | | | | | | | | | | | |
| --- | --- | --- | --- | --- | --- | --- | --- | --- | --- | --- | --- | --- | --- | --- | --- | --- |
|  |  |  |  |  |  |  |  | **Year** |  |  |  |  |  |  |  |  |
|  | **2006** | **2007** | **2008** | **2009** | **2010** | **2011** | **2012** | **2013** | **2014** | **2015** | **2016** | **2017** | **2018** | **2019** | **2020** | ***P for trend*** |
| **Boys** |  |  |  |  |  |  |  |  |  |  |  |  |  |  |  |  |
| **Household income** |  |  |  |  |  |  |  |  |  |  |  |  |  |  |  |  |
| High | 1.00 |  |  |  |  |  |  |  |  |  |  |  |  |  |  |  |
| Middle | 0.91  (0.80-1.04) | 1.00  (0.88-1.14) | 0.96  (0.86-1.07) | 0.95  (0.85-1.07) | 0.96  (0.86-1.08) | 0.95  (0.86-1.06) | 0.86  (0.78-0.94) | 1.02  (0.92-1.12) | 1.05  (0.95-1.15) | 0.97  (0.88-1.06) | 0.99  (0.90-1.08) | 0.99  (0.91-1.08) | 1.02  (0.94-1.10) | 0.97  (0.90-1.05) | 1.04  (0.96-1.13) | *0.286* |
| Low | 1.00  (0.86-1.16) | 1.11  (0.98-1.27) | 1.31  (1.16-1.48) | 1.14  (1.01-1.29) | 1.05  (0.92-1.20) | 1.05  (0.93-1.19) | 1.11  (1.00-1.24) | 1.21  (1.06-1.37) | 1.36  (1.21-1.53) | 1.22  (1.08-1.39) | 1.29  (1.14-1.45) | 1.38  (1.23-1.56) | 1.17  (1.03-1.32) | 1.33  (1.18-1.49) | 1.32  (1.18-1.47) | *0.009* |
| **Father’s education** |  |  |  |  |  |  |  |  |  |  |  |  |  |  |  |  |
| Tertiary or above | 1.00 |  |  |  |  |  |  |  |  |  |  |  |  |  |  |  |
| Upper secondary | 1.03  (0.91-1.16) | 1.11  (0.99-1.23) | 1.20  (1.09-1.32) | 0.99  (0.88-1.12) | 1.17  (1.05-1.31) | 1.14  (1.03-1.27) | 1.17  (1.07-1.29) | 1.21  (1.10-1.34) | 1.18  (1.07-1.30) | 1.35  (1.23-1.48) | 1.24  (1.14-1.34) | 1.37  (1.25-1.50) | 1.21  (1.11-1.31) | 1.35  (1.21-1.52) | 1.41  (1.28-1.55) | *0.001* |
| Basic or less | 0.95  (0.78-1.15) | 1.37  (1.12-1.66) | 1.19  (1.00-1.43) | 1.04  (0.85-1.27) | 0.97  (0.80-1.18) | 1.28  (1.06-1.56) | 1.32  (1.03-1.69) | 1.37  (1.09-1.73) | 1.49  (1.19-1.88) | 1.17  (0.91-1.51) | 1.61  (1.32-1.96) | 1.61  (1.28-2.04) | 1.19  (0.90-1.59) | 1.59  (1.16-2.19) | 1.57  (1.11-2.22) | *0.148* |
| **Mother’s education** |  |  |  |  |  |  |  |  |  |  |  |  |  |  |  |  |
| Tertiary or above | 1.00 |  |  |  |  |  |  |  |  |  |  |  |  |  |  |  |
| Upper secondary | 1.15  (1.01-1.32) | 1.15  (1.02-1.29) | 1.06  (0.95-1.18) | 0.97  (0.86-1.10) | 1.07  (0.95-1.20) | 1.04  (0.94-1.14) | 1.17  (1.07-1.28) | 1.18  (1.08-1.29) | 1.18  (1.07-1.29) | 1.30  (1.20-1.42) | 1.24  (1.15-1.33) | 1.31  (1.20-1.42) | 1.14  (1.05-1.24) | 1.31  (1.17-1.46) | 1.24  (1.13-1.37) | *0.656* |
| Basic or less | 1.05  (0.88-1.26) | 1.31  (1.09-1.57) | 1.21  (1.02-1.44) | 1.20  (0.96-1.48) | 1.14  (0.92-1.42) | 1.20  (0.96-1.49) | 1.33  (1.05-1.69) | 1.37  (1.10-1.71) | 1.11  (0.85-1.44) | 1.65  (1.29-2.11) | 1.65  (1.32-2.07) | 1.61  (1.22-2.11) | 1.16  (0.84-1.59) | 1.81  (1.29-2.55) | 1.32  (0.89-1.94) | *0.921* |
| **Urbanicity** |  |  |  |  |  |  |  |  |  |  |  |  |  |  |  |  |
| Metropolitan cities | 1.00 |  |  |  |  |  |  |  |  |  |  |  |  |  |  |  |
| Other cities | 0.91  (0.82-1.01) | 0.83  (0.74-0.94) | 0.90  (0.79-1.01) | 0.98  (0.86-1.12) | 1.00  (0.89-1.12) | 0.96  (0.86-1.07) | 1.03  (0.93-1.15) | 1.09  (0.99-1.20) | 0.97  (0.88-1.07) | 0.89  (0.81-0.99) | 0.95  (0.86-1.05) | 0.99  (0.91-1.08) | 0.96  (0.88-1.04) | 0.97  (0.89-1.06) | 0.90  (0.83-0.99) | *0.174* |
| Rural areas | 0.81  (0.65-0.99) | 0.93  (0.81-1.07) | 0.88  (0.76-1.02) | 0.89  (0.76-1.05) | 1.15  (1.01-1.32) | 0.90  (0.77-1.06) | 1.03  (0.86-1.23) | 1.01  (0.84-1.22) | 1.19  (1.02-1.38) | 1.14  (0.92-1.41) | 1.18  (1.00-1.39) | 1.21  (0.98-1.50) | 1.17  (0.97-1.40) | 1.15  (1.00-1.32) | 1.01  (0.83-1.23) | *<0.001* |
| **Girls** |  |  |  |  |  |  |  |  |  |  |  |  |  |  |  |  |
| **Household income** |  |  |  |  |  |  |  |  |  |  |  |  |  |  |  |  |
| High | 1.00 |  |  |  |  |  |  |  |  |  |  |  |  |  |  |  |
| Middle | 1.07  (0.84-1.35) | 1.15  (0.97-1.38) | 1.01  (0.84-1.22) | 0.97  (0.82-1.15) | 1.04  (0.86-1.25) | 1.13  (0.99-1.30) | 1.05  (0.91-1.22) | 1.30  (1.14-1.50) | 1.28  (1.12-1.45) | 1.43  (1.27-1.61) | 1.22  (1.09-1.36) | 1.15  (1.03-1.29) | 1.15  (1.03-1.28) | 1.11  (1.00-1.24) | 1.33  (1.19-1.48) | *0.024* |
| Low | 1.85  (1.45-2.37) | 1.56  (1.28-1.90) | 1.65  (1.37-1.99) | 1.44  (1.19-1.74) | 1.79  (1.44-2.22) | 1.76  (1.50-2.06) | 1.83  (1.57-2.14) | 1.92  (1.65-2.23) | 2.11  (1.82-2.44) | 2.14  (1.86-2.46) | 1.92  (1.68-2.21) | 1.87  (1.62-2.16) | 1.93  (1.66-2.24) | 1.96  (1.71-2.25) | 2.29  (1.99-2.63) | *0.005* |
| **Father’s education** |  |  |  |  |  |  |  |  |  |  |  |  |  |  |  |  |
| Tertiary or above | 1.00 |  |  |  |  |  |  |  |  |  |  |  |  |  |  |  |
| Upper secondary | 1.54  (1.28-1.85) | 1.41  (1.21-1.64) | 1.49  (1.29-1.72) | 1.39  (1.19-1.62) | 1.39  (1.19-1.63) | 1.52  (1.34-1.72) | 1.41  (1.24-1.60) | 1.63  (1.45-1.83) | 1.60  (1.43-1.78) | 1.63  (1.46-1.83) | 1.68  (1.51-1.87) | 1.62  (1.46-1.80) | 1.71  (1.52-1.93) | 1.62  (1.42-1.85) | 1.85  (1.65-2.08) | *0.013* |
| Basic or less | 2.08  (1.68-2.58) | 1.50  (1.17-1.92) | 1.50  (1.14-1.96) | 2.09  (1.64-2.66) | 2.45  (1.90-3.17) | 2.10  (1.71-2.58) | 2.14  (1.73-2.64) | 2.20  (1.77-2.73) | 2.12  (1.70-2.65) | 2.09  (1.61-2.71) | 2.00  (1.57-2.55) | 2.81  (2.20-3.60) | 2.18  (1.60-2.97) | 2.43  (1.68-3.52) | 2.41  (1.73-3.36) | *0.121* |
| **Mother’s education** |  |  |  |  |  |  |  |  |  |  |  |  |  |  |  |  |
| Tertiary or above | 1.00 |  |  |  |  |  |  |  |  |  |  |  |  |  |  |  |
| Upper secondary | 1.49  (1.23-1.80) | 1.22  (1.04-1.43) | 1.33  (1.12-1.59) | 1.27  (1.08-1.49) | 1.33  (1.12-1.57) | 1.36  (1.19-1.55) | 1.32  (1.16-1.50) | 1.53  (1.36-1.71) | 1.60  (1.43-1.79) | 1.71  (1.53-1.91) | 1.53  (1.39-1.70) | 1.45  (1.31-1.62) | 1.66  (1.50-1.83) | 1.55  (1.37-1.76) | 1.69  (1.50-1.90) | *0.007* |
| Basic or less | 2.18  (1.75-2.73) | 1.81  (1.45-2.26) | 1.69  (1.30-2.21) | 1.64  (1.24-2.18) | 2.21  (1.69-2.88) | 2.14  (1.72-2.65) | 2.16  (1.72-2.72) | 1.78  (1.37-2.33) | 2.45  (1.89-3.16) | 2.47  (1.87-3.25) | 2.18  (1.64-2.90) | 2.71  (2.10-3.50) | 1.82  (1.29-2.56) | 2.19  (1.47-3.26) | 2.00  (1.30-3.08) | *0.926* |
| **Urbanicity** |  |  |  |  |  |  |  |  |  |  |  |  |  |  |  |  |
| Metropolitan cities | 1.00 |  |  |  |  |  |  |  |  |  |  |  |  |  |  |  |
| Other cities | 1.02  (0.83-1.26) | 0.95  (0.79-1.13) | 1.15  (0.98-1.34) | 1.05  (0.87-1.26) | 1.19  (0.99-1.43) | 1.05  (0.92-1.19) | 1.07  (0.94-1.22) | 1.07  (0.95-1.21) | 1.09  (0.96-1.23) | 1.19  (1.05-1.36) | 1.09  (0.97-1.23) | 1.01  (0.90-1.14) | 1.11  (0.98-1.26) | 0.97  (0.87-1.09) | 1.02  (0.91-1.15) | *0.938* |
| Rural areas | 1.26  (1.07-1.49) | 1.08  (0.88-1.32) | 1.22  (1.00-1.50) | 1.31  (1.08-1.60) | 1.52  (1.24-1.87) | 1.23  (1.04-1.45) | 1.46  (1.20-1.77) | 1.30  (1.09-1.56) | 1.34  (1.09-1.64) | 1.55  (1.28-1.87) | 1.68  (1.33-2.11) | 1.45  (1.18-1.78) | 1.57  (1.32-1.88) | 1.60  (1.32-1.94) | 1.37  (1.16-1.62) | *0.006* |

| **Supplementary Material 6 *(continued)*. Odds ratio and 95% confidence intervals by socioeconomic status from 2006 to 2020 (according to school stage)** | | | | | | | | | | | | | | | | |
| --- | --- | --- | --- | --- | --- | --- | --- | --- | --- | --- | --- | --- | --- | --- | --- | --- |
|  |  |  |  |  |  |  |  | **Year** |  |  |  |  |  |  |  |  |
|  | **2006** | **2007** | **2008** | **2009** | **2010** | **2011** | **2012** | **2013** | **2014** | **2015** | **2016** | **2017** | **2018** | **2019** | **2020** | ***P for trend*** |
| **High school** |  |  |  |  |  |  |  |  |  |  |  |  |  |  |  |  |
| **Household income** |  |  |  |  |  |  |  |  |  |  |  |  |  |  |  |  |
| High | 1.00 |  |  |  |  |  |  |  |  |  |  |  |  |  |  |  |
| Middle | 0.84  (0.71-0.99) | 0.93  (0.83-1.06) | 0.88  (0.77-1.00) | 0.90  (0.79-1.01) | 1.01  (0.87-1.17) | 0.88  (0.78-0.99) | 0.84  (0.76-0.94) | 0.93  (0.84-1.03) | 0.99  (0.91-1.09) | 0.95  (0.86-1.04) | 0.98  (0.90-1.08) | 0.87  (0.80-0.95) | 0.9  (0.83-0.98) | 0.95  (0.87-1.03) | 1.04  (0.95-1.13) | *0.019* |
| Low | 1.10  (0.92-1.31) | 1.11  (0.96-1.28) | 1.32  (1.16-1.51) | 1.16  (1.01-1.32) | 1.26  (1.08-1.46) | 1.16  (1.02-1.32) | 1.22  (1.08-1.37) | 1.15  (1.01-1.29) | 1.33  (1.18-1.50) | 1.25  (1.11-1.41) | 1.28  (1.15-1.43) | 1.29  (1.16-1.44) | 1.16  (1.03-1.30) | 1.37  (1.23-1.52) | 1.46  (1.30-1.63) | *<0.001* |
| **Father’s education** |  |  |  |  |  |  |  |  |  |  |  |  |  |  |  |  |
| Tertiary or above | 1.00 |  |  |  |  |  |  |  |  |  |  |  |  |  |  |  |
| Upper secondary | 1.14  (0.99-1.3) | 1.15  (1.03-1.29) | 1.26  (1.14-1.40) | 0.99  (0.87-1.13) | 1.22  (1.09-1.36) | 1.10  (0.98-1.23) | 1.11  (1.00-1.23) | 1.23  (1.11-1.36) | 1.20  (1.09-1.33) | 1.31  (1.201.43) | 1.24  (1.14-1.34) | 1.27  (1.16-1.39) | 1.25  (1.15-1.36) | 1.27  (1.14-1.41) | 1.44  (1.31-1.60) | *<0.001* |
| Basic or less | 1.25  (1.06-1.49) | 1.22  (0.99-1.51) | 1.26  (1.04-1.53) | 1.29  (1.07-1.55) | 1.43  (1.18-1.73) | 1.32  (1.10-1.57) | 1.50  (1.24-1.82) | 1.42  (1.16-1.73) | 1.49  (1.21-1.84) | 1.18  (0.95-1.48) | 1.44  (1.16-1.79) | 1.71  (1.40-2.08) | 1.32  (1.04-1.68) | 1.64  (1.25-2.16) | 1.80  (1.31-2.47) | *<0.001* |
| **Mother’s education** |  |  |  |  |  |  |  |  |  |  |  |  |  |  |  |  |
| Tertiary or above | 1.00 |  |  |  |  |  |  |  |  |  |  |  |  |  |  |  |
| Upper secondary | 1.23  (1.05-1.45) | 1.09  (0.97-1.23) | 1.05  (0.93-1.18) | 0.94  (0.83-1.07) | 1.07  (0.95-1.22) | 0.96  (0.86-1.06) | 1.14  (1.03-1.27) | 1.14  (1.03-1.27) | 1.17  (1.07-1.28) | 1.28  (1.18-1.40 | 1.28  (1.18-1.40) | 1.21  (1.11-1.32) | 1.14  (1.05-1.23) | 1.23  (1.11-1.37) | 1.31  (1.18-1.44) | *0.014* |
| Basic or less | 1.28  (1.07-1.55) | 1.28  (1.07-1.52) | 1.31  (1.08-1.59) | 1.21  (0.98-1.50) | 1.36  (1.10-1.69) | 1.34  (1.11-1.60) | 1.42  (1.16-1.73) | 1.42  (1.16-1.73) | 1.39  (1.12-1.73) | 1.73  (1.39-2.14) | 1.73  (1.39-2.14) | 1.59  (1.28-1.98) | 1.21  (0.93-1.59) | 1.77  (1.30-2.41) | 1.23  (0.85-1.78) | *0.096* |
| **Urbanicity** |  |  |  |  |  |  |  |  |  |  |  |  |  |  |  |  |
| Metropolitan cities | 1.00 |  |  |  |  |  |  |  |  |  |  |  |  |  |  |  |
| Other cities | 0.92  (0.80-1.07) | 0.82  (0.71-0.95) | 0.92  (0.79-1.07) | 0.90  (0.79-1.02) | 0.98  (0.84-1.13) | 0.86  (0.76-0.98) | 1.00  (0.89-1.12) | 1.02  (0.92-1.13) | 0.96  (0.86-1.06) | 0.99  (0.89-1.10 | 0.98  (0.88-1.08) | 0.96  (0.87-1.05) | 0.97  (0.87-1.07) | 0.94  (0.85-1.04) | 0.96  (0.86-1.06) | *0.717* |
| Rural areas | 0.89  (0.76-1.04) | 0.91  (0.78-1.06) | 0.98  (0.85-1.13) | 0.89  (0.74-1.08) | 1.30  (1.10-1.54) | 0.89  (0.77-1.04) | 1.00  (0.85-1.18) | 1.03  (0.86-1.22) | 1.10  (0.89-1.36) | 0.99  (0.86-1.15) | 1.27  (1.06-1.52) | 1.05  (0.92-1.19) | 1.11  (0.93-1.32) | 1.24  (1.08-1.44) | 0.99  (0.82-1.19) | *<0.001* |
| **Middle school** |  |  |  |  |  |  |  |  |  |  |  |  |  |  |  |  |
| **Household income** |  |  |  |  |  |  |  |  |  |  |  |  |  |  |  |  |
| High | 1.00 |  |  |  |  |  |  |  |  |  |  |  |  |  |  |  |
| Middle | 0.93  (0.79-1.09) | 1.03  (0.87-1.21) | 0.95  (0.83-1.08) | 0.89  (0.77-1.04) | 0.83  (0.73-0.93) | 1.03  (0.91-1.15) | 0.89  (0.79-0.99) | 1.13  (1.00-1.29) | 1.10  (0.98-1.24) | 1.19  (1.05-1.35) | 0.98  (0.88-1.09) | 1.12  (1.01-1.25) | 1.12  (1.02-1.24) | 0.94  (0.85-1.04) | 1.07  (0.96-1.18) | *0.215* |
| Low | 1.17  (0.97-1.43) | 1.19  (0.98-1.43) | 1.27  (1.07-1.52) | 1.19  (1.03-1.37) | 1.12  (0.94-1.33) | 1.22  (1.03-1.44) | 1.3  (1.13-1.5) | 1.58  (1.36-1.84) | 1.78  (1.54-2.05) | 1.59  (1.35-1.87) | 1.62  (1.39-1.9) | 1.59  (1.36-1.85) | 1.60  (1.37-1.87) | 1.47  (1.28-1.69) | 1.59  (1.39-1.82) | *0.143* |
| **Father’s education** |  |  |  |  |  |  |  |  |  |  |  |  |  |  |  |  |
| Tertiary or above | 1.00 |  |  |  |  |  |  |  |  |  |  |  |  |  |  |  |
| Upper secondary | 1.16  (0.99-1.35) | 1.20  (1.04-1.38) | 1.28  (1.13-1.44) | 1.22  (1.05-1.42) | 1.21  (1.06-1.38) | 1.45  (1.27-1.65) | 1.41  (1.26-1.59) | 1.46  (1.31-1.64) | 1.42  (1.27-1.57) | 1.54  (1.35-1.75) | 1.54  (1.39-1.70) | 1.66  (1.48-1.87) | 1.45  (1.29-1.63) | 1.61  (1.41-1.84) | 1.58  (1.41-1.76) | *0.051* |
| Basic or less | 1.12  (0.89-1.41) | 1.64  (1.31-2.05) | 1.25  (0.96-1.62) | 1.34  (1.02-1.75) | 1.24  (0.93-1.64) | 1.92  (1.50-2.45) | 1.59  (1.21-2.08) | 1.99  (1.52-2.59) | 2.05  (1.61-2.61) | 2.15  (1.55-3.00) | 2.35  (1.79-3.09) | 2.53  (1.86-3.44) | 1.76  (1.24-2.50) | 2.12  (1.35-3.35) | 1.78  (1.26-2.52) | *0.855* |
| **Mother’s education** |  |  |  |  |  |  |  |  |  |  |  |  |  |  |  |  |
| Tertiary or above | 1.00 |  |  |  |  |  |  |  |  |  |  |  |  |  |  |  |
| Upper secondary | 1.18  (1.01-1.38) | 1.13  (0.97-1.33) | 1.17  (1.02-1.33) | 1.12  (0.95-1.33) | 1.13  (0.99-1.30) | 1.31  (1.17-1.47) | 1.25  (1.12-1.38) | 1.41  (1.27-1.57) | 1.40  (1.25-1.57) | 1.51  (1.35-1.70) | 1.41  (1.27-1.57) | 1.39  (1.25-1.55) | 1.45  (1.30-1.63) | 1.47  (1.29-1.68) | 1.35  (1.21-1.51) | *0.410* |
| Basic or less | 1.34  (1.05-1.70) | 1.55  (1.21-1.98) | 1.18  (0.88-1.59) | 1.33  (0.96-1.83) | 1.45  (1.08-1.93) | 1.55  (1.16-2.06) | 1.88  (1.44-2.44) | 1.56  (1.19-2.06) | 1.57  (1.14-2.17) | 1.82  (1.27-2.62) | 2.23  (1.58-3.14) | 2.69  (2.00-3.63) | 1.40  (0.92-2.14) | 1.79  (1.15-2.78) | 1.96  (1.28-3.01) | *0.309* |
| **Urbanicity** |  |  |  |  |  |  |  |  |  |  |  |  |  |  |  |  |
| Metropolitan cities | 1.00 |  |  |  |  |  |  |  |  |  |  |  |  |  |  |  |
| Other cities | 0.99  (0.86-1.14) | 0.96  (0.82-1.14) | 1.06  (0.91-1.23) | 1.15  (0.96-1.38) | 1.14  (0.97-1.34) | 1.19  (1.05-1.34) | 1.12  (0.99-1.27) | 1.19  (1.05-1.36) | 1.11  (0.98-1.27) | 1.04  (0.90-1.19) | 1.05  (0.92-1.20) | 1.10  (0.97-1.24) | 1.09  (0.97-1.23) | 1.03  (0.92-1.15) | 0.94  (0.83-1.06) | *0.044* |
| Rural areas | 1.06  (0.84-1.33) | 1.10  (0.91-1.32) | 1.01  (0.82-1.24) | 1.23  (1.00-1.51) | 1.24  (1.01-1.53) | 1.23  (1.01-1.5) | 1.51  (1.25-1.83) | 1.32  (1.04-1.68) | 1.51  (1.24-1.85) | 1.95  (1.49-2.54) | 1.54  (1.19-1.98) | 1.86  (1.34-2.60 | 1.73  (1.39-2.15) | 1.38  (1.12-1.71) | 1.37  (1.06-1.76) | *0.012* |
