# Supplementary material for: Trends in socioeconomic inequalities in obesity among Korean adolescents: the Korea Youth Risk Behavior Web-based Survey (KYRBS) 2006 to 2020
Source: Epidemiol Health. 2023 Mar 7;45:e2023033. doi: 10.4178/epih.e2023033 (PMC10586920; doi:10.4178/epih.e2023033)
Supplement: Supplementary Material 7. — Relative Index of Inequality (RII) based on the odds ratio from 2006 to 2020 [file epih-45-e2023033-Supplementary-7.docx]

| **Supplementary Material 7. Relative Index of Inequality (RII) based on the odds ratio from 2006 to 2020** | | | | | | | | | | | | | | | | |
| --- | --- | --- | --- | --- | --- | --- | --- | --- | --- | --- | --- | --- | --- | --- | --- | --- |
|  |  |  |  |  |  |  |  | **Year** |  |  |  |  |  |  |  |  |
|  | **2006** | **2007** | **2008** | **2009** | **2010** | **2011** | **2012** | **2013** | **2014** | **2015** | **2016** | **2017** | **2018** | **2019** | **2020** | ***P for trend*** |
| **Household income** |  |  |  |  |  |  |  |  |  |  |  |  |  |  |  |  |
| Total | 1.22 (1.03-1.46) | 1.29 (1.09-1.52) | 1.50 (1.28-1.75) | 1.24 (1.06-1.44) | 1.23 (1.05-1.45) | 1.29 (1.12-1.48) | 1.36 (1.19-1.55) | 1.49 (1.31-1.70) | 1.68 (1.48-1.91) | 1.60 (1.40-1.82) | 1.48 (1.31-1.67) | 1.45 (1.29-1.64) | 1.34 (1.19-1.51) | 1.39 (1.25-1.55) | 1.57 (1.40-1.76) | *0.004* |
| Boys | 0.96 (0.77-1.18) | 1.14 (0.95-1.38) | 1.39 (1.16-1.67) | 1.17 (0.98-1.39) | 1.05 (0.88-1.25) | 1.04 (0.87-1.24) | 1.07 (0.91-1.25) | 1.24 (1.04-1.48) | 1.42 (1.20-1.67) | 1.19 (1.01-1.41) | 1.26 (1.08-1.47) | 1.31 (1.13-1.52) | 1.14 (0.99-1.32) | 1.22 (1.06-1.40) | 1.30 (1.13-1.50) | *0.024* |
| Girls | 2.32 (1.62-3.32) | 1.84 (1.40-2.41) | 2.04 (1.55-2.67) | 1.67 (1.27-2.19) | 2.28 (1.65-3.16) | 2.18 (1.74-2.73) | 2.33 (1.84-2.93) | 2.39 (1.94-2.93) | 2.64 (2.14-3.26) | 2.74 (2.27-3.31) | 2.20 (1.81-2.68) | 2.04 (1.67-2.5) | 2.05 (1.66-2.54) | 2.04 (1.68-2.47) | 2.67 (2.21-3.23) | *0.225* |
| High school | 1.16 (0.90-1.49) | 1.17 (0.96-1.43) | 1.56 (1.29-1.88) | 1.26 (1.03-1.53) | 1.38 (1.12-1.71) | 1.25 (1.04-1.50) | 1.35 (1.13-1.60) | 1.19 (1.00-1.42) | 1.45 (1.22-1.71) | 1.28 (1.09-1.52) | 1.31 (1.12-1.52) | 1.21 (1.04-1.41) | 1.07 (0.91-1.24) | 1.31 (1.14-1.50) | 1.48 (1.27-1.72) | *0.805* |
| Middle school | 1.11 (0.85-1.44) | 1.22 (0.93-1.60) | 1.28 (0.99-1.64) | 1.15 (0.93-1.44) | 1.00 (0.79-1.25) | 1.24 (0.99-1.55) | 1.21 (0.99-1.49) | 1.70 (1.38-2.09) | 1.79 (1.46-2.18) | 1.70 (1.37-2.11) | 1.41 (1.15-1.73) | 1.55 (1.29-1.87) | 1.56 (1.29-1.88) | 1.21 (1.01-1.44) | 1.48 (1.25-1.76) | *0.002* |
|  |  |  |  |  |  |  |  |  |  |  |  |  |  |  |  |  |
| **Father’s education** |  |  |  |  |  |  |  |  |  |  |  |  |  |  |  |  |
| Total | 1.40 (1.18-1.67) | 1.54 (1.29-1.83) | 1.60 (1.37-1.86) | 1.32 (1.10-1.59) | 1.55 (1.32-1.82) | 1.68 (1.46-1.95) | 1.68 (1.45-1.94) | 1.89 (1.64-2.19) | 1.83 (1.58-2.12) | 2.06 (1.79-2.38) | 1.97 (1.74-2.24) | 2.22 (1.94-2.55) | 1.89 (1.66-2.16) | 2.23 (1.89-2.63) | 2.44 (2.11-2.82) | *<0.001* |
| Boys | 1.00 (0.80-1.24) | 1.37 (1.10-1.70) | 1.40 (1.17-1.68) | 1.01 (0.80-1.26) | 1.24 (1.02-1.51) | 1.36 (1.13-1.63) | 1.42 (1.18-1.73) | 1.51 (1.25-1.84) | 1.49 (1.23-1.81) | 1.74 (1.45-2.09) | 1.62 (1.39-1.88) | 1.92 (1.61-2.29) | 1.44 (1.23-1.70) | 1.88 (1.51-2.34) | 2.00 (1.65-2.43) | *<0.001* |
| Girls | 2.65 (1.98-3.56) | 1.93 (1.46-2.54) | 2.09 (1.59-2.73) | 2.33 (1.75-3.10) | 2.54 (1.87-3.44) | 2.55 (2.05-3.17) | 2.29 (1.81-2.89) | 2.84 (2.29-3.52) | 2.70 (2.18-3.33) | 2.78 (2.25-3.45) | 2.88 (2.35-3.51) | 2.95 (2.41-3.62) | 3.03 (2.42-3.79) | 2.81 (2.19-3.59) | 3.55 (2.84-4.45) | *<0.001* |
| High school | 1.35 (1.08-1.69) | 1.34 (1.07-1.69) | 1.52 (1.26-1.83) | 1.16 (0.92-1.46) | 1.57 (1.30-1.91) | 1.30 (1.08-1.58) | 1.40 (1.16-1.69) | 1.56 (1.29-1.89) | 1.54 (1.26-1.88) | 1.64 (1.38-1.95) | 1.58 (1.34-1.85) | 1.72 (1.46-2.03) | 1.58 (1.34-1.86) | 1.69 (1.38-2.09) | 2.16 (1.77-2.62) | *<.0001* |
| Middle school | 1.30 (0.99-1.71) | 1.62 (1.26-2.09) | 1.58 (1.24-2.01) | 1.52 (1.13-2.05) | 1.46 (1.12-1.90) | 2.26 (1.79-2.84) | 2.03 (1.62-2.54) | 2.29 (1.85-2.85) | 2.17 (1.77-2.66) | 2.51 (1.96-3.23) | 2.54 (2.07-3.11) | 2.95 (2.35-3.70) | 2.16 (1.73-2.70) | 2.69 (2.07-3.48) | 2.53 (2.05-3.13) | *<.0001* |
|  |  |  |  |  |  |  |  |  |  |  |  |  |  |  |  |  |
| **Mother’s education** |  |  |  |  |  |  |  |  |  |  |  |  |  |  |  |  |
| Total | 1.52 (1.28-1.81) | 1.50 (1.26-1.79) | 1.37 (1.15-1.62) | 1.20 (0.98-1.47) | 1.38 (1.15-1.64) | 1.40 (1.21-1.62) | 1.60 (1.38-1.85) | 1.66 (1.44-1.90) | 1.70 (1.48-1.94) | 2.14 (1.87-2.46) | 1.82 (1.61-2.06) | 1.89 (1.65-2.15) | 1.68 (1.48-1.92) | 2.02 (1.72-2.37) | 1.89 (1.63-2.18) | *<0.001* |
| Boys | 1.17 (0.95-1.45) | 1.38 (1.11-1.71) | 1.19 (0.99-1.44) | 1.06 (0.83-1.36) | 1.17 (0.95-1.44) | 1.14 (0.94-1.37) | 1.42 (1.18-1.70) | 1.43 (1.20-1.70) | 1.35 (1.13-1.62) | 1.76 (1.49-2.09) | 1.60 (1.39-1.86) | 1.75 (1.48-2.06) | 1.30 (1.10-1.53) | 1.78 (1.44-2.20) | 1.55 (1.28-1.88) | *<0.001* |
| Girls | 2.60 (1.97-3.44) | 1.90 (1.43-2.52) | 1.90 (1.39-2.60) | 1.77 (1.32-2.37) | 2.23 (1.62-3.07) | 2.19 (1.72-2.79) | 2.08 (1.63-2.66) | 2.33 (1.87-2.91) | 2.78 (2.24-3.46) | 3.10 (2.50-3.84) | 2.47 (2.02-3.01) | 2.38 (1.94-2.92) | 2.75 (2.24-3.36) | 2.51 (1.96-3.22) | 2.88 (2.28-3.64) | *0.001* |
| High school | 1.40 (1.11-1.75) | 1.32 (1.06-1.64) | 1.29 (1.03-1.61) | 1.07 (0.83-1.38) | 1.32 (1.04-1.66) | 1.11 (0.91-1.35) | 1.41 (1.16-1.72) | 1.32 (1.09-1.59) | 1.41 (1.19-1.68) | 1.75 (1.48-2.08) | 1.49 (1.28-1.73) | 1.54 (1.30-1.81) | 1.30 (1.11-1.52) | 1.61 (1.32-1.96) | 1.69 (1.39-2.05) | *0.001* |
| Middle school | 1.44 (1.09-1.90) | 1.48 (1.11-1.99) | 1.34 (1.04-1.71) | 1.32 (0.95-1.85) | 1.39 (1.07-1.80) | 1.77 (1.42-2.21) | 1.73 (1.40-2.13) | 2.00 (1.64-2.45) | 1.98 (1.59-2.45) | 2.32 (1.85-2.91) | 2.12 (1.72-2.62) | 2.12 (1.72-2.61) | 2.10 (1.68-2.62) | 2.20 (1.70-2.86) | 1.91 (1.53-2.38) | *<0.001* |
|  |  |  |  |  |  |  |  |  |  |  |  |  |  |  |  |  |
| **Urbanicity** |  |  |  |  |  |  |  |  |  |  |  |  |  |  |  |  |
| Total | 0.91 (0.75-1.09) | 0.80 (0.66-0.98) | 0.96 (0.79-1.16) | 1.02 (0.82-1.25) | 1.22 (1.00-1.49) | 0.99 (0.84-1.17) | 1.16 (0.99-1.36) | 1.19 (1.02-1.39) | 1.14 (0.97-1.33) | 1.14 (0.97-1.34) | 1.14 (0.98-1.34) | 1.13 (0.97-1.31) | 1.15 (0.99-1.33) | 1.08 (0.94-1.24) | 0.97 (0.84-1.13) | *0.015* |
| Boys | 0.78 (0.63-0.96) | 0.73 (0.58-0.91) | 0.79 (0.63-0.99) | 0.93 (0.73-1.18) | 1.08 (0.88-1.32) | 0.89 (0.73-1.09) | 1.06 (0.87-1.29) | 1.14 (0.95-1.37) | 1.05 (0.87-1.25) | 0.91 (0.74-1.11) | 1 (0.83-1.20) | 1.08 (0.89-1.29) | 1 (0.85-1.19) | 1.01 (0.86-1.18) | 0.86 (0.73-1.02) | *0.012* |
| Girls | 1.19 (0.85-1.67) | 0.96 (0.70-1.31) | 1.36 (1.02-1.81) | 1.22 (0.86-1.72) | 1.61 (1.17-2.21) | 1.18 (0.93-1.50) | 1.33 (1.04-1.70) | 1.27 (1.02-1.59) | 1.3 (1.03-1.63) | 1.58 (1.25-1.99) | 1.44 (1.14-1.82) | 1.22 (0.98-1.52) | 1.44 (1.14-1.82) | 1.2 (0.95-1.50) | 1.19 (0.96-1.47) | *0.435* |
| High school | 0.84 (0.65-1.07) | 0.70 (0.54-0.91) | 0.87 (0.66-1.14) | 0.8 (0.62-1.03) | 1.13 (0.86-1.47) | 0.75 (0.60-0.95) | 1 (0.81-1.23) | 1.04 (0.86-1.26) | 0.98 (0.80-1.20) | 0.99 (0.82-1.19) | 1.07 (0.87-1.30) | 0.96 (0.81-1.14) | 0.99 (0.82-1.20) | 1.01 (0.84-1.21) | 0.93 (0.77-1.12) | *0.008* |
| Middle school | 1.02 (0.78-1.34) | 0.99 (0.74-1.33) | 1.1 (0.83-1.45) | 1.37 (0.99-1.90) | 1.35 (1.00-1.82) | 1.43 (1.14-1.80) | 1.45 (1.15-1.84) | 1.49 (1.17-1.91) | 1.43 (1.13-1.82) | 1.5 (1.12-2.00) | 1.31 (1.02-1.69) | 1.52 (1.16-2.00) | 1.49 (1.18-1.89) | 1.19 (0.96-1.48) | 1.04 (0.82-1.31) | *0.261* |
|  |  |  |  |  |  |  |  |  |  |  |  |  |  |  |  |  |
