# Supplementary material for: Trends in socioeconomic inequalities in obesity among Korean adolescents: the Korea Youth Risk Behavior Web-based Survey (KYRBS) 2006 to 2020
Source: Epidemiol Health. 2023 Mar 7;45:e2023033. doi: 10.4178/epih.e2023033 (PMC10586920; doi:10.4178/epih.e2023033)
Supplement: Supplementary Material 8. — Slope Index of Inequality (SII) based on the prevalence difference from 2006 to 2020 [file epih-45-e2023033-Supplementary-8.docx]

| **Supplementary Material 8. Slope Index of Inequality (SII) based on the prevalence difference from 2006 to 2020** | | | | | | | | | | | | | | | | |
| --- | --- | --- | --- | --- | --- | --- | --- | --- | --- | --- | --- | --- | --- | --- | --- | --- |
|  |  |  |  |  |  |  |  | **Year** |  |  |  |  |  |  |  |  |
|  | **2006** | **2007** | **2008** | **2009** | **2010** | **2011** | **2012** | **2013** | **2014** | **2015** | **2016** | **2017** | **2018** | **2019** | **2020** | ***P for trend*** |
| **Household income** |  |  |  |  |  |  |  |  |  |  |  |  |  |  |  |  |
| Total | 0.84  (0.14-1.53) | 1.38  (0.71-2.04) | 1.91  (1.27-2.54) | 1.08  (0.44-1.73) | 0.86  (0.19-1.53) | 1.01  (0.35-1.66) | 1.41  (0.72-2.09) | 2.3  (1.55-3.05) | 2.79  (2.03-3.55) | 3.09  (2.26-3.92) | 3.16  (2.24-4.08) | 3.05  (2.06-4.04) | 2.82  (1.76-3.87) | 2.98  (1.93-4.03) | 4.45  (3.31-5.59) | *0.007* |
| Boys | -0.3  (-1.4-0.79) | 1.09  (0.05-2.13) | 1.71  (0.71-2.71) | 0.97  (-0.02-1.96) | 0.17  (-0.89-1.22) | -0.15  (-1.17-0.88) | 0.24  (-0.81-1.29) | 1.17  (0.02-2.33) | 2.32  (1.12-3.52) | 1.49  (0.24-2.73) | 2.26  (0.84-3.67) | 2.36  (0.81-3.9) | 2.08  (0.41-3.74) | 2.25  (0.67-3.83) | 3.45  (1.68-5.22) | *0.044* |
| Girls | 2.68  (1.84-3.52) | 2.18  (1.38-2.97) | 2.71  (1.94-3.49) | 1.85  (1.06-2.65) | 2.42  (1.63-3.21) | 2.75  (1.93-3.57) | 3.12  (2.25-4) | 4.13  (3.19-5.08) | 3.96  (3.03-4.89) | 5.28  (4.21-6.36) | 4.84  (3.69-5.99) | 4.83  (3.59-6.07) | 4.65  (3.36-5.95) | 4.87  (3.53-6.2) | 6.54  (5.15-7.93) | *<0.001* |
| High school | 0.56  (-0.47-1.58) | 0.8  (-0.17-1.77) | 2.14  (1.21-3.07) | 1.05  (0.15-1.95) | 1.25  (0.29-2.2) | 1.04  (0.12-1.96) | 1.17  (0.22-2.13) | 1.31  (0.24-2.38) | 2.29  (1.23-3.36) | 1.71  (0.52-2.91) | 2.55  (1.25-3.85) | 1.91  (0.51-3.31) | 0.87  (-0.64-2.39) | 2.98  (1.43-4.52) | 4.17  (2.51-5.83) | *0.056* |
| Middle school | 0.31  (-0.66-1.27) | 1.25  (0.34-2.15) | 1.16  (0.26-2.05) | 0.96  (0.03-1.89) | 0.21  (-0.74-1.17) | 0.55  (-0.4-1.51) | 1.36  (0.35-2.37) | 2.67  (1.59-3.75) | 2.72  (1.61-3.83) | 3.31  (2.13-4.49) | 2.54  (1.22-3.85) | 2.92  (1.49-4.35) | 3.41  (1.93-4.9) | 1.34  (-0.07-2.75) | 3.68  (2.09-5.26) | *0.003* |
|  |  |  |  |  |  |  |  |  |  |  |  |  |  |  |  |  |
| **Father’s education** |  |  |  |  |  |  |  |  |  |  |  |  |  |  |  |  |
| Total | 1.18  (0.44-1.91) | 1.84  (1.13-2.55) | 1.8  (1.08-2.51) | 1.48  (0.78-2.18) | 1.92  (1.19-2.66) | 2.62  (1.89-3.34) | 2.87  (2.09-3.64) | 4.17  (3.34-5.01) | 4.3  (3.42-5.17) | 5.18  (4.22-6.15) | 5.61  (4.54-6.69) | 7.58  (6.39-8.78) | 6.59  (5.31-7.88) | 8.61  (6.91-10.3) | 9.89  (8.21-11.58) | *<0.001* |
| Boys | -0.38  (-1.53-0.77) | 1.88  (0.76-3.01) | 1.53  (0.4-2.65) | 0.59  (-0.51-1.69) | 1.55  (0.37-2.72) | 1.84  (0.7-2.99) | 2.33  (1.14-3.51) | 3.07  (1.78-4.36) | 3.78  (2.4-5.16) | 4.5  (3.04-5.96) | 5.06  (3.41-6.72) | 7.38  (5.53-9.23) | 4.92  (2.94-6.9) | 7.89  (5.17-10.62) | 9.39  (6.65-12.13) | *0.001* |
| Girls | 2.91  (2.03-3.8) | 1.9  (1.04-2.76) | 2.31  (1.45-3.18) | 2.59  (1.74-3.44) | 2.36  (1.5-3.23) | 3.66  (2.75-4.57) | 3.53  (2.54-4.52) | 5.4  (4.34-6.47) | 4.99  (3.91-6.08) | 6.07  (4.81-7.33) | 6.44  (5.08-7.8) | 7.96  (6.45-9.47) | 8.48  (6.84-10.11) | 9.12  (7.03-11.2) | 10.74  (8.7-12.78) | *<0.001* |
| High school | 0.6  (-0.45-1.66) | 1.27  (0.23-2.3) | 1.46  (0.44-2.49) | 0.81  (-0.16-1.77) | 1.62  (0.6-2.64) | 1.64  (0.63-2.65) | 1.73  (0.68-2.79) | 3.22  (2.06-4.39) | 3.38  (2.17-4.58) | 3.98  (2.62-5.33) | 4.15  (2.68-5.63) | 5.97  (4.34-7.6) | 5.71  (3.91-7.5) | 6.74  (4.29-9.19) | 9.67  (7.24-12.09) | *0.001* |
| Middle school | 1.3  (0.28-2.31) | 2.03  (1.05-3.01) | 1.87  (0.87-2.86) | 2.19  (1.16-3.22) | 2.14  (1.07-3.21) | 3.52  (2.46-4.59) | 4.1  (2.95-5.24) | 4.97  (3.76-6.17) | 5.05  (3.76-6.34) | 5.75  (4.37-7.13) | 6.66  (5.08-8.24) | 8.5  (6.72-10.28) | 6.5  (4.67-8.33) | 8.86  (6.52-11.21) | 9.15  (6.8-11.49) | *<0.001* |
|  |  |  |  |  |  |  |  |  |  |  |  |  |  |  |  |  |
| **Mother’s education** |  |  |  |  |  |  |  |  |  |  |  |  |  |  |  |  |
| Total | 1.29  (0.53-2.05) | 1.65  (0.93-2.37) | 1.4  (0.68-2.13) | 1.02  (0.31-1.74) | 1.56  (0.82-2.29) | 1.66  (0.95-2.37) | 2.58  (1.83-3.34) | 3.21  (2.4-4.03) | 3.5  (2.66-4.33) | 5.31  (4.4-6.23) | 4.74  (3.71-5.76) | 5.48  (4.36-6.61) | 5.37  (4.15-6.58) | 7.02  (5.41-8.63) | 7.4  (5.81-8.99) | *<0.001* |
| Boys | 0.24  (-0.97-1.45) | 1.75  (0.6-2.9) | 0.98  (-0.16-2.12) | 0.75  (-0.37-1.86) | 1.29  (0.12-2.46) | 0.9  (-0.22-2.02) | 2.06  (0.89-3.23) | 2.64  (1.37-3.91) | 2.62  (1.29-3.96) | 4.62  (3.23-6.02) | 4.75  (3.16-6.35) | 5.81  (4.04-7.58) | 3.92  (2.02-5.83) | 7.05  (4.4-9.7) | 6.88  (4.26-9.51) | *0.001* |
| Girls | 2.62  (1.71-3.52) | 1.86  (1-2.72) | 2.28  (1.41-3.15) | 1.81  (0.93-2.69) | 2.19  (1.34-3.04) | 2.79  (1.91-3.67) | 3.33  (2.37-4.28) | 4.25  (3.23-5.27) | 4.75  (3.73-5.76) | 6.41  (5.24-7.59) | 5.25  (3.98-6.53) | 5.74  (4.35-7.13) | 7.39  (5.87-8.92) | 7.44  (5.5-9.39) | 8.75  (6.85-10.64) | *<0.001* |
| High school | 0.36  (-0.77-1.48) | 1.44  (0.37-2.5) | 0.96  (-0.09-2.01) | 0.19  (-0.82-1.2) | 0.99  (-0.04-2.02) | 0.7  (-0.32-1.71) | 1.93  (0.87-3) | 1.75  (0.59-2.91) | 2.53  (1.37-3.7) | 4.41  (3.12-5.71) | 3.56  (2.13-4.98) | 4.19  (2.63-5.75) | 3.43  (1.74-5.13) | 5.56  (3.18-7.93) | 7.22  (4.91-9.53) | *0.003* |
| Middle school | 1.54  (0.5-2.58) | 1.2  (0.21-2.19) | 1.42  (0.41-2.43) | 1.81  (0.78-2.83) | 2  (0.95-3.06) | 2.38  (1.36-3.4) | 3.08  (1.99-4.17) | 4.36  (3.2-5.52) | 4.14  (2.92-5.35) | 5.24  (3.94-6.54) | 5.15  (3.67-6.64) | 5.65  (4.01-7.28) | 6.23  (4.46-7.99) | 6.61  (4.43-8.79) | 6.59  (4.4-8.79) | *<0.001* |
|  |  |  |  |  |  |  |  |  |  |  |  |  |  |  |  |  |
| **Urbanicity** |  |  |  |  |  |  |  |  |  |  |  |  |  |  |  |  |
| Total | -0.57  (-1.29-0.15) | -0.13  (-0.81-0.55) | 0.62  (-0.07-1.31) | 0.32  (-0.38-1.01) | 1.39  (0.68-2.09) | 0.22  (-0.47-0.91) | 0.76  (0.03-1.49) | 1.45  (0.67-2.24) | 1.13  (0.33-1.93) | 1.76  (0.9-2.62) | 1.85  (0.9-2.8) | 1.91  (0.88-2.94) | 2.04  (0.96-3.13) | 1.13  (0.04-2.21) | 0.37  (-0.81-1.54) | *0.043* |
| Boys | -1.64  (-2.78--0.49) | -0.68  (-1.74-0.39) | -0.31  (-1.38-0.77) | -0.6  (-1.68-0.47) | 0.79  (-0.32-1.9) | -0.47  (-1.54-0.61) | -0.04  (-1.15-1.07) | 1.23  (0.02-2.45) | 0.71  (-0.54-1.95) | 0.2  (-1.08-1.49) | 0.78  (-0.7-2.26) | 1.62  (0.01-3.23) | 1.1  (-0.6-2.79) | 0.44  (-1.2-2.09) | -0.77  (-2.59-1.04) | *0.134* |
| Girls | 0.69  (-0.19-1.56) | 0.4  (-0.44-1.23) | 1.65  (0.79-2.51) | 1.42  (0.57-2.27) | 2.24  (1.39-3.09) | 0.92  (0.06-1.79) | 1.69  (0.75-2.62) | 1.65  (0.65-2.65) | 1.44  (0.45-2.43) | 3.25  (2.12-4.38) | 2.77  (1.59-3.96) | 1.78  (0.5-3.06) | 2.95  (1.6-4.3) | 2.06  (0.69-3.42) | 2.12  (0.69-3.56) | *0.012* |
| High school | -1.25  (-2.31--0.19) | -0.43  (-1.42-0.56) | 0.25  (-0.77-1.26) | -0.41  (-1.37-0.55) | 1.21  (0.23-2.2) | -1  (-1.97--0.02) | -0.32  (-1.35-0.7) | 0.54  (-0.59-1.66) | 0.09  (-1.03-1.22) | 0.88  (-0.38-2.14) | 1.51  (0.15-2.87) | 0.6  (-0.87-2.06) | 0.73  (-0.86-2.32) | 0.65  (-0.95-2.25) | 0.41  (-1.3-2.12) | *0.248* |
| Middle school | 0.11  (-0.87-1.08) | 0.19  (-0.74-1.12) | 0.99  (0.05-1.93) | 1.11  (0.12-2.1) | 1.56  (0.54-2.57) | 1.59  (0.62-2.56) | 2  (0.96-3.03) | 2.5  (1.41-3.58) | 2.38  (1.27-3.5) | 2.63  (1.49-3.77) | 2.41  (1.1-3.72) | 3.54  (2.12-4.95) | 3.6  (2.15-5.05) | 1.7  (0.25-3.15) | 0.37  (-1.22-1.97) | *0.005* |
