# Supplementary material for: Trends in socioeconomic inequalities in obesity among Korean adolescents: the Korea Youth Risk Behavior Web-based Survey (KYRBS) 2006 to 2020
Source: Epidemiol Health. 2023 Mar 7;45:e2023033. doi: 10.4178/epih.e2023033 (PMC10586920; doi:10.4178/epih.e2023033)
Supplement: Supplementary Material 9. — Prevalence of stunting according to socioeconomic status [file epih-45-e2023033-Supplementary-9.docx]

| **Supplementary Material 9. Prevalence of stunting according to socioeconomic status** | | | | | | | | | | | | | | | |
| --- | --- | --- | --- | --- | --- | --- | --- | --- | --- | --- | --- | --- | --- | --- | --- |
|  |  |  |  |  |  |  |  | **Year** |  |  |  |  |  |  |  |
|  | **2006** | **2007** | **2008** | **2009** | **2010** | **2011** | **2012** | **2013** | **2014** | **2015** | **2016** | **2017** | **2018** | **2019** | **2020** |
| **Household income (%)** |  |  |  |  |  |  |  |  |  |  |  |  |  |  |  |
| High | 260  (1.4) | 155  (1.0) | 183  (0.9) | 174  (0.9) | 175  (0.9) | 176  (0.9) | 181  (0.9) | 179  (0.9) | 175  (0.9) | 147  (0.7) | 189  (0.9) | 201  (1.0) | 148  (0.7) | 162  (0.8) | 148  (0.8) |
| Middle | 445  (1.7) | 435  (1.4) | 419  (1.3) | 372  (1.2) | 395  (1.3) | 389  (1.3) | 378  (1.4) | 343  (1.2) | 351  (1.3) | 335  (1.4) | 303  (1.3) | 287  (1.4) | 201  (0.9) | 253  (1.1) | 241  (1.2) |
| Low | 276  (2.5) | 285  (1.8) | 289  (1.9) | 272  (1.7) | 260  (1.8) | 243  (1.8) | 245  (2.0) | 237  (2.0) | 188  (1.9) | 150  (1.8) | 125  (1.7) | 130  (2.3) | 102  (1.9) | 118  (2.0) | 81  (1.6) |
| **Father's education (%)** |  |  |  |  |  |  |  |  |  |  |  |  |  |  |  |
| Tertiary or above | 325  (1.5) | 265  (1.0) | 310  (1.1) | 279  (0.9) | 321  (1.0) | 302  (1.0) | 337  (1.1) | 288  (0.9) | 320  (1.0) | 289  (0.9) | 301  (1.0) | 312  (1.1) | 247  (0.8) | 177  (1.0) | 204  (1.0) |
| Upper secondary | 466  (1.7) | 387  (1.4) | 373  (1.3) | 372  (1.4) | 362  (1.4) | 387  (1.5) | 359  (1.5) | 370  (1.6) | 300  (1.5) | 265  (1.5) | 248  (1.5) | 239  (1.6) | 158  (1.1) | 95  (1.2) | 104  (1.2) |
| Basic or less | 190  (3.2) | 129  (2.3) | 116  (2.6) | 106  (2.3) | 105  (2.9) | 66  (1.7) | 62  (2.2) | 54  (2.4) | 53  (2.6) | 31  (2.4) | 26  (1.9) | 24  (2.1) | 15  (1.5) | 12  (2.0) | 6  (1.7) |
| **Mother's education (%)** |  |  |  |  |  |  |  |  |  |  |  |  |  |  |  |
| Tertiary or above | 207  (1.5) | 177  (1.0) | 212  (1.0) | 201  (0.9) | 231  (1.0) | 231  (1.0) | 257  (1.1) | 249  (0.9) | 267  (1.0) | 235  (0.8) | 265  (1.0) | 281  (1.0) | 224  (0.8) | 152  (0.8) | 183  (0.9) |
| Upper secondary | 568  (1.7) | 448  (1.3) | 473  (1.3) | 467  (1.3) | 469  (1.3) | 460  (1.4) | 437  (1.4) | 411  (1.4) | 352  (1.4) | 330  (1.5) | 283  (1.4) | 278  (1.6) | 192  (1.1) | 129  (1.4) | 128  (1.3) |
| Basic or less | 206  (2.8) | 153  (2.3) | 109  (2.2) | 92  (1.9) | 90  (2.2) | 70  (2.0) | 62  (2.4) | 55  (2.4) | 41  (2.3) | 28  (2.3) | 27  (2.5) | 23  (3.0) | 9  (1.4) | 8  (1.6) | 10  (3.5) |
| **Urbanicity (%)** |  |  |  |  |  |  |  |  |  |  |  |  |  |  |  |
| Metropolitan cities | 400  (1.4) | 356  (1.2) | 407  (1.2) | 372  (1.1) | 329  (1.1) | 323  (1.1) | 340  (1.3) | 324  (1.2) | 290  (1.2) | 254  (1.1) | 254  (1.1) | 254  (1.2) | 194  (0.9) | 215  (1.1) | 193  (1.0) |
| Other cities | 401  (2.0) | 356  (1.4) | 344  (1.4) | 305  (1.3) | 351  (1.4) | 360  (1.4) | 359  (1.4) | 344  (1.3) | 345  (1.2) | 311  (1.2) | 310  (1.2) | 302  (1.3) | 212  (0.9) | 259  (1.1) | 225  (1.1) |
| Rural areas | 180  (2.5) | 163  (2.0) | 140  (2.0) | 141  (2.1) | 150  (1.8) | 125  (1.5) | 105  (1.6) | 91  (1.2) | 79  (1.9) | 67  (1.6) | 53  (1.4) | 62  (1.8) | 45  (1.3) | 59  (1.8) | 52  (1.4) |
| Stunting was defined as height-for-age less than two standard deviations from the median based on the 2017 KNGC.  The prevalence was calculated as the proportion of stunting adolescents in each of three socioeconomic groups. | | | | | | | | | | | | | | | |
