# Supplementary material for: Trends in socioeconomic inequalities in obesity among Korean adolescents: the Korea Youth Risk Behavior Web-based Survey (KYRBS) 2006 to 2020
Source: Epidemiol Health. 2023 Mar 7;45:e2023033. doi: 10.4178/epih.e2023033 (PMC10586920; doi:10.4178/epih.e2023033)
Supplement: Supplementary Material 10. — Prevalence of the coexistence of stunting and obesity according to socioeconomic status [file epih-45-e2023033-Supplementary-10.docx]

| **Supplementary Material 10. Prevalence of the coexistence of stunting and obesity according to socioeconomic status** | | | | | | | | | | | | | | | |
| --- | --- | --- | --- | --- | --- | --- | --- | --- | --- | --- | --- | --- | --- | --- | --- |
|  |  |  |  |  |  |  |  | **Year** |  |  |  |  |  |  |  |
|  | **2006** | **2007** | **2008** | **2009** | **2010** | **2011** | **2012** | **2013** | **2014** | **2015** | **2016** | **2017** | **2018** | **2019** | **2020** |
| **Household income (%)** |  |  |  |  |  |  |  |  |  |  |  |  |  |  |  |
| High | 13  (0.8) | 5  (0.6) | 7  (0.4) | 8  (0.8) | 4  (0.3) | 10  (0.8) | 5  (0.3) | 6  (0.5) | 5  (0.4) | 13  (1.1) | 15  (1) | 18  (1.1) | 11  (0.5) | 13  (0.6) | 21  (0.9) |
| Middle | 27  (1.6) | 21  (1.4) | 18  (1.5) | 13  (1) | 17  (1.5) | 15  (0.9) | 22  (1.3) | 21  (1.2) | 18  (1.1) | 22  (1.3) | 26  (1.2) | 32  (1.8) | 14  (0.7) | 26  (1.2) | 26  (0.9) |
| Low | 24  (2.5) | 16  (1.7) | 21  (2.1) | 18  (1.3) | 16  (1.6) | 14  (1.3) | 12  (1.5) | 19  (2) | 15  (1.7) | 12  (1.6) | 13  (1.7) | 17  (2.3) | 20  (3.1) | 11  (1) | 9  (1.2) |
| **Father's education (%)** |  |  |  |  |  |  |  |  |  |  |  |  |  |  |  |
| Tertiary or above | 16  (0.8) | 11  (0.9) | 18  (1.4) | 13  (0.8) | 13  (0.6) | 11  (0.5) | 14  (0.7) | 12  (0.8) | 16  (0.8) | 20  (1.1) | 27  (1.2) | 30  (1.4) | 26  (1) | 18  (1) | 24  (1) |
| Upper secondary | 35  (2) | 14  (0.8) | 17  (1) | 16  (1) | 14  (1.3) | 21  (1.3) | 19  (1.3) | 26  (1.5) | 15  (1.1) | 21  (1.4) | 23  (1.4) | 26  (1.5) | 13  (1) | 12  (1.1) | 15  (0.8) |
| Basic or less | 13  (2.7) | 8  (2.3) | 5  (1.9) | 5  (1.2) | 8  (3.4) | 1  (0.5) | 4  (1.3) | 6  (2) | 5  (2.4) | 3  (3.4) | 1  (0.6) | 3  (1.9) | 3  (1.8) | 2  (1.3) | 6  (1.7) |
| **Mother's education (%)** |  |  |  |  |  |  |  |  |  |  |  |  |  |  |  |
| Tertiary or above | 11  (1.1) | 8  (1.1) | 12  (0.9) | 11  (1) | 4  (0.2) | 13  (0.9) | 11  (0.8) | 11  (0.8) | 15  (0.9) | 13  (0.9) | 20  (0.9) | 24  (1.2) | 22  (0.9) | 16  (0.9) | 24  (1) |
| Upper secondary | 39  (1.6) | 21  (1.1) | 24  (1.3) | 23  (1.1) | 26  (1.7) | 18  (0.8) | 23  (1.2) | 30  (1.4) | 18  (0.9) | 27  (1.5) | 31  (1.6) | 38  (2) | 18  (1) | 14  (1.1) | 15  (0.8) |
| Basic or less | 14  (2.4) | 8  (1.7) | 5  (1.4) | 3  (0.3) | 5  (1.2) | 6  (2.5) | 4  (1.5) | 5  (2.5) | 3  (2) | 3  (2.3) | 1  (0.9) | 2  (2) | 2  (1.6) | 2  (1.4) | 1  (0.7) |
| **Urbanicity (%)** |  |  |  |  |  |  |  |  |  |  |  |  |  |  |  |
| Metropolitan cities | 23  (1.1) | 19  (1.4) | 25  (1.4) | 18  (1.1) | 17  (0.9) | 18  (1) | 21  (1.5) | 16  (1.1) | 19  (1.2) | 17  (1.1) | 28  (1.5) | 29  (1.5) | 18  (0.9) | 19  (0.9) | 22  (0.9) |
| Other cities | 29  (1.9) | 15  (1) | 16  (1.3) | 15  (0.9) | 15  (1.4) | 16  (0.9) | 14  (0.7) | 24  (1.1) | 15  (0.8) | 28  (1.6) | 25  (1.1) | 30  (1.5) | 23  (1) | 26  (1) | 25  (0.8) |
| Rural areas | 12  (2.7) | 8  (1.6) | 5  (1.6) | 6  (1.4) | 5  (0.6) | 5  (1.7) | 4  (0.9) | 6  (1.9) | 4  (0.9) | 2  (0.7) | 1  (0.4) | 8  (2.6) | 4  (1.3) | 5  (0.8) | 9  (2.1) |
| The prevalence was calculated as the proportion of stunting among obese adolescents in each of three socioeconomic groups. | | | | | | | | | | | | | | | |
