# Supplementary material for: Trends in socioeconomic inequalities in obesity among Korean adolescents: the Korea Youth Risk Behavior Web-based Survey (KYRBS) 2006 to 2020
Source: Epidemiol Health. 2023 Mar 7;45:e2023033. doi: 10.4178/epih.e2023033 (PMC10586920; doi:10.4178/epih.e2023033)
Supplement: Supplementary Material 11. — Prevalence of adolescent obesity based on physical measurement data in KNHANES [file epih-45-e2023033-Supplementary-11.docx]

| **Supplementary Material 11. Prevalence of adolescent obesity based on physical measurement data in KNHANES** | | | | | | | | | | | | | | | | | | |
| --- | --- | --- | --- | --- | --- | --- | --- | --- | --- | --- | --- | --- | --- | --- | --- | --- | --- | --- |
|  | **IV (2007-2009)** | | |  | **V (2010-2012)** | | |  | **VI (2013-2015)** | | |  | **VII (2016-2018)** | | |  | **VIII* (2019-2020)** | |
| **Year** | **2007** | **2008** | **2009** |  | **2010** | **2011** | **2012** |  | **2013** | **2014** | **2015** |  | **2016** | **2017** | **2018** |  | **2019** | **2020** |
| No. of cases (%) | 185  (9.2) | | |  | 198  (10.1) | | |  | 186  (11.4) | | |  | 191  (12.4) | | |  | 145  (16.1) | |
| No. of cases (%) | 35  (11.3) | 74  (8.5) | 76  (7.8) |  | 71  (9.3) | 68  (10.8) | 59  (10.2) |  | 56  (9.2) | 57  (11.4) | 73  (13.9) |  | 68  (13) | 58  (10.9) | 65  (13.3) |  | 70  (15.6) | 75  (16.6) |
| KNHANES, Korea National Health and Nutrition Examination Survey.  * The 8th KNHANES consists of a three-year survey from 2019 to 2021, the data of 2021 was not released yet and not included in this analysis. | | | | | | | | | | | | | | | | | | |
